# Visible-light Promoted Atom Transfer Radical Addition−Elimination (ATRE) Reaction for the Synthesis of Fluoroalkylated Alkenes Using DMA as Electron-donor

**DOI:** 10.3390/molecules25030508

**Published:** 2020-01-24

**Authors:** Wen-Wen Xu, Le Wang, Ting Mao, Jiwei Gu, Xiao-Fei Li, Chun-Yang He

**Affiliations:** 1 Key Laboratory of Biocatalysis & Chiral Drug Synthesis of Guizhou Province, Generic Drug Research Center of Guizhou Province. School of Pharmacy, Zunyi Medical University, Zunyi 563003, China; xuww20191213@163.com (W.-W.X.); 18585310741@163.com (T.M.); 2 Basic Medical School, Zunyi Medical University, Zunyi 563003, China; wangle526@sina.com; 3 School of Medicine, Washington University in St. Louis, St. Louis, MO 63110, United States; jiweigu@wustl.edu; 4 Key Laboratory of Basic Pharmacology of Ministry of Education and Joint International Research Laboratory of Ethnomedicine of Ministry of Education, School of Pharmacy, Zunyi Medical University, Zunyi 563003, China

**Keywords:** fluoroalkyl iodides, EDA, noncovalent interaction, fluoroalkylated alkenes, DMA

## Abstract

Here, we describe a mild, catalyst-free and operationally-simple strategy for the direct fluoroalkylation of olefins driven by the photochemical activity of an electron donor−acceptor (EDA) complex between DMA and fluoroalkyl iodides. The significant advantages of this photochemical transformation are high efficiency, excellent functional group tolerance, and synthetic simplicity, thus providing a facile route for further application in pharmaceuticals and life sciences.

## 1. Introduction

Owing to their tendency to alter the lipophilicity, metabolic stability, and electronic properties of organic molecules, fluorinated organic compounds have been widely used in medicinal chemistry, and material sciences [1,2,3,4,5,6,7,8,9]. Therefore, the development of safer, less toxic and more selective methods to introduce fluorinated functional groups into organic molecules has become an intensive topic of synthetic organic chemistry [10,11,12,13,14,15,16,17,18,19].

Alkenes play a ubiquitous role in the realm of chemical synthesis due to their enriched reactivity and abundance, fluoroalkylation of carbon-carbon double bonds is an attractive method for accessing fluorine containing compounds. Since 1945, atom transfer radical addition (ATRA) has extensively been utilized for fluoroalkylation of alkenes [20,21,22,23,24,25,26,27,28,29]. However, the preparation of alkenes containing fluorinated functional groups via Heck-type reaction were less studied owning to the lack of efficient and general strategies [30,31,32]. In the past 10 years, some powerful strategies have been developed to realize such transformation [20,21,33,34,35,36]. One of the major improvements has been made via photo-excited catalyst such as Ru/Ir complexes and organic dyes [21,33]. Very recently, non-covalent interaction initiated fluoroalkylation reaction has emerged as an attractive strategy [37,38,39,40,41,42,43,44,45,46,47]. Inspired by our previous studies in this field [48,49,50,51], we envision that if the solvent can serve as an electron donor compound, the reactions would be simpler. Here, we demonstrate a mild, catalyst-free and operationally-simple strategy for the direct fluoroalkylation of olefins driven by the photochemical activity of electron donor−acceptor (EDA) complex between DMA and fluoroalkyl iodides. The significant advantages of this photochemical transformation are high efficiency, excellent functional group tolerance, and synthetic simplicity [52]. 

## 2. Results

We initially probed this catalyst-free fluoroalkylation reaction by using readily available *tert*-butyl allylcarbamate **1a** and ethyl iododifluoroacetate **2a** (1.5 equiv) as model substrates. 29% yield of atom transfer radical addition (ATRA) product **4a** was obtained when the reaction was performed with K_3_PO_4_ (2.0 equiv) in MeCN and irradiated by blue LEDs for 16 h. After a series of reaction media were screened (Table 1, entries 2–8), THF, Toluene and dioxane were not suitable for this transformation, and 10% yield of desired product was obtained when DMSO was used as solvent. Both **3a** and **4a** were obtained when DMA and DMF were used. To improve the selectivity of this transformation, a variety of different bases were examined utilizing DMA as solvent for this reaction (Table 1, entries 8–11). Among them, KOAc was the best choice and afforded **3a** in 64% yield (Table 1, entry 11). Finally, different light sources were tested, the yield increased to 95% when the reaction mixtures were irradiated under purple LEDs (Table 1, entry 13), no desired product was observed when control experiments were carried out in the absence of light or base, demonstrating the photochemical nature of this transformation (Table 1, entries 14, 15).

**Scheme 1:**
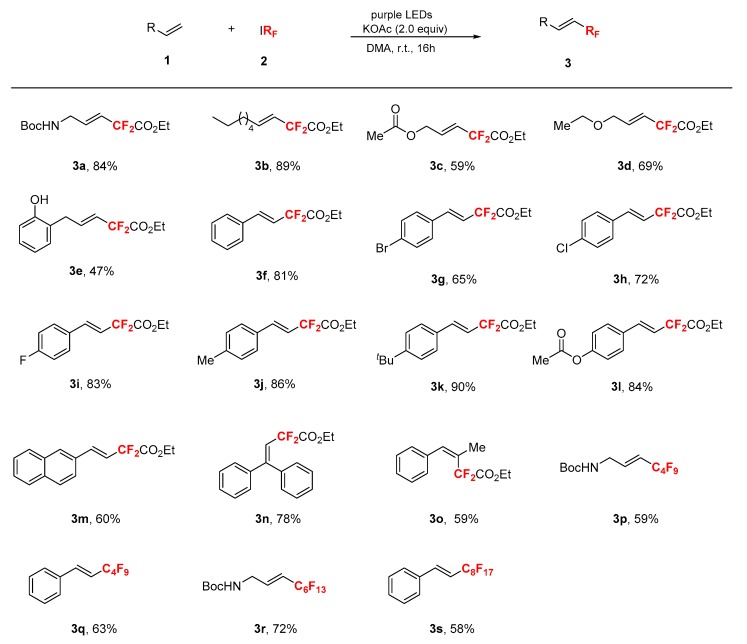
Atom Transfer Radical Addition−Elimination (ATRE) reaction for the synthesis of fluoroalkylated alkenes ^a,b^. ^a^ Reaction conditions (unless otherwise specified): **1** (0.3 mmol, 1.0 equiv), **2** (0.45 mmol, 1.5 equiv), KOAc (0.6 mmol, 2.0 equiv) in anhydrous DMA (2.0 mL), r.t. under Ar, purple LEDs, for 16 h. ^b^ Yield of isolated product.

With the optimum reaction conditions established, various alkenes were explored. As shown in Scheme 1, The reaction exhibited good functional group tolerance. A range of functional groups, such as esters (**3c**), methoxyl (**3d**), and even unprotected hydroxyl group (**3e**) generally were compatible with the reaction and moderate to good yields were obtained. Then, styrenes were also evaluated, and we were pleased to find that styrenes all performed well under the optimized reaction conditions, providing the corresponding products in good to excellent yields (**3f**–**n**). Importantly, substrate with steric hindrance could also undergo this transformation smoothly (**3o**). This reaction system is also amenable to the use of other commercial perfluoroalkyl iodides, such as C_4_F_9_I, C_6_F_13_I and C_8_F_17_I (Scheme 1, **3p**–**s**).

**Scheme 2:**
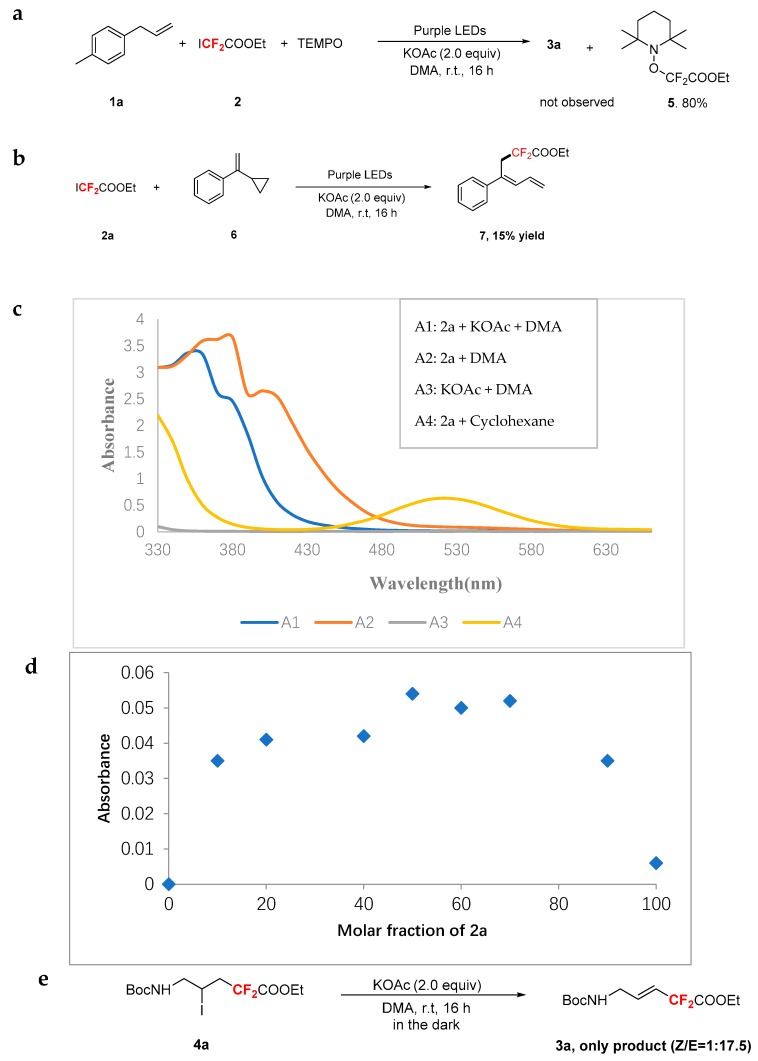
Mechanistic investigation. (**a**) Addition of radical and SET inhibitors. (**b**) Trapping of intermediates. (**c**) Optical absorption spectra study. (**d**) Job’s plot. (**e**) Control experiment.

To gain insight into the mechanism of this reaction, several experiments were performed. The reaction was completely suppressed when a radical scavenger TEMPO (1.0 equiv) was added as an additive under the standard reaction conditions (Scheme 2a). Furthermore, a radical clock experiment was conducted. The ring-expanded product **7** was formed when **2a** was treated with *α*-cyclopropylstyrene (**6**) in the absence of **1a** (Scheme 2b). Optical absorption spectra of the reactants found that the absorption was obvious strengthened when DMA and R_F_I were mixed (Scheme 2c, for details, see Supplementary Materials), which indicated a non-covalent interaction occurred between them. Moreover, this conclusion was further confirmed by a Job’s plot (Scheme 2d, for details, see Supplementary Materials). Finally, compound **4a** was totally converted to **3a** under optimized condition in the dark (Scheme 2e, for details, see Supplementary Materials). This result indicated that the reaction was performed via atom transfer radical addition elimination pathway.

**Scheme 3:**
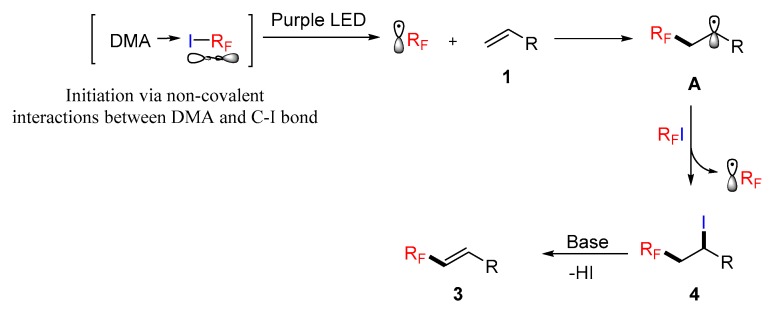
Proposed Reaction Mechanism.

Based on these preliminary results and previous reports [53,54], a plausible mechanism was depicted in Scheme 3. Initially, non-covalent interactions occurred between DMA and C–I bond. Then fluoroalkyl radical was generated under the irradition of purple LEDs. Subsequently, fluoroalkyl radical reacted with alkenes (**1**) and generated a carbon radical **A**, which abstracted an iodine atom from R_F_I to afford ATRA product **4** along with R_F_ radical that sustains the chain. Finally, elimination of **4** with base could afford the corresponding fluoroalkylated-ATRE products (**3**).

## 3. Discussion

In the past 10 years, fluoroalkylation involving visible light promoted reactions has emerged as an attractive and useful strategy to directly introduce fluorinated groups into organic molecules Among those methods, fluoroalkyl iodides were commonly used substrates. Based on the investigations in our group, we believe the noncovalent interaction between solvents (DMF, DMA, MeCN, acetone ect) and fluoroalkyl iodides might play a crucial role in this kind of transformation.

## 4. Materials and Methods

^1^H NMR spectra and ^13^C NMR spectra were recorded on an Agilent AM400 spectrometer. ^19^F NMR was recorded on an Agilent 400 MHz NMR spectrometer (CFCl_3_ as outside standard and low field is positive). Chemical shifts (δ) are reported in ppm, and coupling constants (*J*) are in Hertz (Hz). The following abbreviations were used to explain the multiplicities: s = singlet, d = doublet, t = triplet, q = quartet, m = multiplet, br = broad. NMR yield was determined by ^19^F NMR using fluorobenzene as an internal standard before working up the reaction.

All reagents were used as received from commercial sources, unless specified otherwise, or prepared as described in the literature. All reagents were weighed and handled in air at room temperature. Blue LEDs (430–490 nm, peak wavelength: 455.0 nm) and purple LEDs (380–425 nm, peak wavelength: 395.0 nm) were bought online.

## Figures and Tables

**Table 1:** Representative results for optimization of visible light-mediated reaction of **1a** and **2a**. ^a,b^ 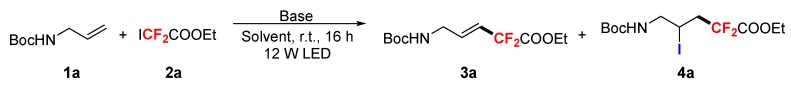

Entry	Light Source	Base (equiv)	Sovent	3a, yield (%)	4a, yield (%)
1	Blue LEDs	K_3_PO_4_ (2)	MeCN	----	29
2	Blue LEDs	K_3_PO_4_ (2)	DCE	----	55
3	Blue LEDs	K_3_PO_4_ (2)	THF	----	----
4	Blue LEDs	K_3_PO_4_ (2)	Toluene	----	7
5	Blue LEDs	K_3_PO_4_ (2)	Dioxane	----	trace
6	Blue LEDs	K_3_PO_4_ (2)	DMSO	10	----
7	Blue LEDs	K_3_PO_4_ (2)	DMF	21	51
8	Blue LEDs	K_3_PO_4_ (2)	DMA	45	36
9	Blue LEDs	K_2_CO_3_ (2)	DMA	43	26
10	Blue LEDs	Cs_2_CO_3_ (2)	DMA	59	5
11	Blue LEDs	KOAc (2)	DMA	64	----
12	Green LEDs	KOAc (2)	DMA	50	----
13	Purple LEDs	KOAc (2)	DMA	95(84)	----
14	Purple LEDs	None	DMA	----	----
15 ^c^	none	KOAc (2)	DMA	----	----

^a^ Reaction conditions: (unless otherwise specified): **1a** (0.3 mmol, 1.0 equiv), **2a** (0.45 mmol, 1.5 equiv), anhydrous solvent (2 mL), r.t. under Ar, irritated under visible light for 16 h. ^b^ Determined by ^19^F NMR spectroscopy using fluorobenzene as an internal standard, and the number within parentheses represents the yield of the isolated product. ^c^ The reaction was performed without light.

## Supplementary Materials

The supplementary materials are available online.
