# Supplementary material for: Visible-light Promoted Atom Transfer Radical Addition−Elimination (ATRE) Reaction for the Synthesis of Fluoroalkylated Alkenes Using DMA as Electron-donor
Source: Molecules. 2020 Jan 24;25(3):508. doi: 10.3390/molecules25030508 (PMC7036948; doi:10.3390/molecules25030508)
Supplement: Supplementary file 1 [file molecules-25-00508-s001.pdf]

Supporting Information for

# Visible-light Promoted Atom Transfer Radical Addition−Elimination (ATRE) Reaction for the Synthesis of Fluoroalkylated Alkenes Using DMA as Eectron-donor

***Wen-Wen Xu,*** [1, †] ***Le Wang,*** [2, †] ***Ting Mao,*** [1] ***Jiwei Gu,*** [4] ***Xiao-Fei Li*** [2, 3]* ***and Chun-Yang He*** [1, 3]*

1 *Key Laboratory of Biocatalysis & Chiral Drug Synthesis of Guizhou Province, Generic Drug Research Center of Guizhou Province. School of Pharmacy, Zunyi Medical University, Zunyi, Guizhou, P.R. China*

2 *Basic Medical School, Zunyi Medical University, Zunyi, Guizhou, China*

3 *Key Laboratory of Basic Pharmacology of Ministry of Education and Joint International Research Laboratory of Ethnomedicine of Ministry of Education. School of Pharmacy, Zunyi Medical University, Zunyi, Guizhou, P.R. China*

4 *School of MedicineWashington, University in St. Louis, St. Louis, Missouri, United States*

# List of Contents


| | |
|---|---|
| 1) General procedure | S3 |
| 2) Data for compounds **3** | S3 |
| 3) Mechanism studies | S10 |
| 4) References | S17 |
| 5) Copies of NMR spectra of **3** | S18 |

## 1. General procedure.

**General procedure for visible-light promoted atom transfer radical addition−elimination (ATRE) reaction for the synthesis of fluoroalkylated alkenes using DMA as electron-donor.** To a 25 mL of Schlenk tube equipped with a Teflon septum were added KOAc (0.6 mmol, 2.0 equiv.) under Ar, followed by DMA (2 mL). Then, alkene (**1**) (0.3 mmol, 1.0 equiv.), and $IR_F$ (**2**) (0.45 mmol, 1.5 equiv.) were added subsequently. After stirring under purple light for 16 hours, the residue was diluted with ethyl acetate, washed with $H_2O$ and brine, dried over $Na_2SO_4$, filtered and concentrated. The residue was purified with silica gel chromatography to provide pure product.

## 2. Data for compounds 3

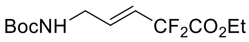


**Ethyl (*E*)-5-((tert-butoxycarbonyl)amino)-2,2-difluoropent-3-enoate (3a).** This compound is known.[1] The product (100 mg, 84% yield, *Z:E* =1:40) was purified with silica gel chromatography (Petroleum ether/Ethyl acetate = 5:1) as colorless liquid. $^1$H NMR (400 MHz, $CDCl_3$) δ 6.25 (d, *J* = 16.0 Hz, 1H), 5.85–5.73 (m, 1H), 4.80 (br, 1H), 4.29 (q, *J* = 7.1 Hz, 2H), 3.84 (s, 2H), 1.42 (s, 9H), 1.31 (t, *J* = 7.1 Hz, 3H). $^{19}$F NMR (376 MHz, $CDCl_3$) δ -103.5 (s, 2F). $^{13}$C NMR (101 MHz, $CDCl_3$) δ 163.7 (t, *J* = 34.6 Hz), 155.5, 136.1 (t, *J* = 8.7 Hz), 121.4 (t, *J* = 25.5 Hz), 112.1 (t, *J* = 249.2 Hz), 79.8, 63.0, 41.0, 28.2, 13.8.

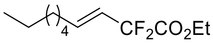


**Ethyl (*E*)-2,2-difluorodec-3-enoate (3b).** This compound is known.[2] The product (62.5 mg, 89% yield, *Z:E* = 1:9) was purified with silica gel chromatography (Petroleum ether/Ethyl acetate = 100:1) as colorless liquid. $^1$H NMR (400 MHz, $CDCl_3$) δ 6.31–6.21 (m, 1H), 5.71–5.60 (m, 1H), 4.31 (q, *J* = 6.8 Hz, 2H), 2.17–2.08

(m, 2H), 1.45–1.20 (m, 11H), 0.87 (t, *J* = 6.8 Hz, 3H). $^{19}$F NMR (376 MHz, $CDCl_3$) δ -103.0 (m, 2F). $^{13}$C NMR (101 MHz, $CDCl_3$) δ 164.1 (t, *J* = 35.0 Hz), 139.9 (t, *J* = 9.1 Hz), 120.9 (t, *J* =25.0 Hz), 112.3 (t, *J* =248.6 Hz), 62.8, 31.8, 31.5, 28.6, 28.0, 22.5, 13.9, 13.8.

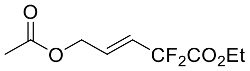


**Ethyl (*E*)-5-acetoxy-2,2-difluoropent-3-enoate (3c).** This compound is known.[1] The product (39.6 mg, 59% yield, *Z:E* = 1:8.7) was purified with silica gel chromatography (Petroleum ether/Ethyl acetate = 5:1) as colorless liquid. $^{1}$H NMR (400 MHz, $CDCl_3$) δ 6.37–6.29 (m, 1H), 6.00–5.89 (m, 1H), 4.70–4.65 (m, 2H), 4.33 (q, *J* = 7.2 Hz, 2H), 2.11 (s, 3H), 1.35 (t, *J* = 7.2 Hz, 3H). $^{19}$F NMR (376 MHz, $CDCl_3$) δ -111.5 (d, *J* = 10.2 Hz, 2F). $^{13}$C NMR (101 MHz, $CDCl_3$) δ 170.2, 163.5 (t, *J* = 34.4 Hz), 132.8 (t, *J* = 9.2 Hz), 122.7 (t, *J* = 25.6 Hz), 111.8 (t, *J* = 249.5 Hz), 63.1, 62.3, 20.7, 13.9.

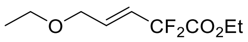


**Ethyl (*E*)-5-ethoxy-2,2-difluoropent-3-enoate (3d).** The product (43.1 mg, 69% yield, *Z:E* = 1:14) was purified with silica gel chromatography (Petroleum ether/Ethyl acetate = 50:1) as colorless liquid. $^{1}$H NMR (400 MHz, $CDCl_3$) δ 6.37–6.28 (m, 1H), 6.01–5.90 (m, 1H), 4.31 (q, *J* = 7.2 Hz, 2H), 4.09–4.04 (m, 2H), 3.51 (q, *J* = 7.2 Hz, 2H), 1.34 (t, *J* = 7.2 Hz, 3H), 1.22 (t, *J* = 7.2 Hz, 3H). $^{19}$F NMR (376 MHz, $CDCl_3$) δ -111.0 (s, 2F). $^{13}$C NMR (101 MHz, $CDCl_3$) δ 163.8 (t, *J* = 34.8 Hz), 135.9 (t, *J* = 8.8 Hz), 121.2 (t, *J* = 25.4 Hz), 112.3 (t, *J* = 248.9 Hz), 68.8, 66.4, 63.0, 15.1, 13.9. MS (EI): m/z (%) 208 (M+, 100), 188, 107. HRMS (EI): Calculated for $C_9H_{14}F_2O_3$ ($M^+$): 208.0911; Found: 208.0909.

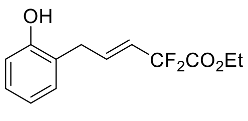

**Ethyl (*E*)-2,2-difluoro-5-(2-hydroxyphenyl)pent-3-enoate (3e).** This compound is known.[3] The product (36.2 mg, 47% yield) was purified with silica gel chromatography (Petroleum ether/Ethyl acetate = 20:1) as colorless liquid. $^1$H NMR (400 MHz, $CDCl_3$) δ 7.16–7.06 (m, 2H), 6.92–6.87 (m, 1H), 6.79–6.75 (m, 1H), 6.52–6.43 (m, 1H), 5.75–5.64 (m, 1H), 5.04 (br, 1H), 4.32 (q, *J* = 7.2 Hz, 2H), 3.51–3.45 (m, 2H), 1.33 (t, *J* = 7.2 Hz, 3H). $^{19}$F NMR (376 MHz, $CDCl_3$) δ -103.0 (s, 2F). $^{13}$C NMR (101 MHz, $CDCl_3$) δ 164.2 (t, *J* = 34.5 Hz), 153.5, 137.7 (t, *J* = 9.1 Hz), 130.5, 128.1, 124.2, 121.9 (t, *J* = 25.0 Hz), 121.0, 115.5, 112.3 (t, *J* = 249.1 Hz), 63.0, 32.5, 13.9.

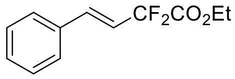


**Ethyl (*E*)-2,2-difluoro-4-phenylbut-3-enoate (3f).** This compound is known.[3] The product (55 mg, 81% yield) was purified with silica gel chromatography (Petroleum ether/Ethyl acetate = 50:1) as colorless liquid. $^1$H NMR (400 MHz, $CDCl_3$) δ 7.46 (d, *J* = 6.4 Hz, 2H), 7.38 (d, *J* = 6.4 Hz, 3H), 7.09 (d, *J* = 16.4 Hz, 1H), 6.37–6.26 (m, 1H), 4.36 (q, *J* = 7.2 Hz, 2H), 1.37 (t, *J* = 7.0 Hz, 3H). $^{19}$F NMR (376 MHz, $CDCl_3$) δ -110.5 (dd, *J* = 10.2 Hz, 1.9 Hz, 2F). $^{13}$C NMR (101 MHz, $CDCl_3$) δ 169.3 (t, *J* = 35.1 Hz), 136.8 (t, *J* = 9.4 Hz), 134.0, 129.6, 128.8, 127.4, 118.8 (t, *J* = 25.1 Hz), 112.7 (t, *J* = 250.5 Hz), 63.1, 13.9.

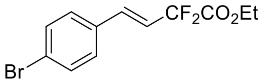


**Ethyl (*E*)-4-(4-bromophenyl)-2,2-difluorobut-3-enoate (3g).** This compound is known.[3] The product (59.3 mg, 65% yield) was purified with silica gel chromatography (Petroleum ether/Ethyl acetate = 20:1) as colorless liquid. $^1$H NMR (400 MHz, $CDCl_3$) δ 7.50 (d, *J* =8.4 Hz, 2H), 7.31 (d, *J* = 8.4 Hz, 2H), 7.02 (dt, *J* = 16.0 Hz, 2.4 Hz, 1H), 6.35–6.25 (m, 1H), 4.35 (q, *J* = 7.2 Hz, 2H), 1.36 (t, *J* = 7.2 Hz, 3H), $^{19}$F NMR (376 MHz, $CDCl_3$) δ -103.4 (d, *J* =11.3 Hz, 2F). $^{13}$C NMR (101 MHz,

$CDCl_3$) δ 163.7 (t, $J$ = 34.9 Hz), 135.6 (t, $J$ =9.6 Hz), 133.0 (t, $J$ =1.2 Hz), 132.0, 128.9, 123.7, 119.5 (t, $J$ = 25.0 Hz), 112.5 (t, $J$ = 250.0 Hz), 63.2, 13.9.

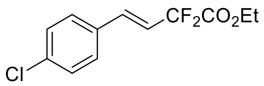


**Ethyl (*E*)-4-(4-chlorophenyl)-2,2-difluorobut-3-enoate (3h).** This compound is known.[3] The product (55.9 mg, 72% yield) was purified with silica gel chromatography (Petroleum ether/Ethyl acetate = 50:1) as colorless liquid. $^1$H NMR (400 MHz, $CDCl_3$) δ 7.40–7.32 (m, 4H), 7.03 (dt, $J$ = 16.0 Hz, 2.4 Hz, 1H), 6.34–6.23 (m, 1H), 4.35 (q, $J$ = 7.2 Hz, 2H), 1.36 (t, $J$ = 7.2 Hz, 3H). $^{19}$F NMR (376 MHz, $CDCl_3$) δ -103.4 (d, $J$ =10.9 Hz, 2F). $^{13}$C NMR (101 MHz, $CDCl_3$) δ 163.7 (t, $J$ = 34.8 Hz), 135.5 (t, $J$ =9.5 Hz), 135.4, 132.5, 129.0, 128.6, 119.4 (t, $J$ = 25.0 Hz), 112.5 (t, $J$ = 250.0 Hz), 63.2, 13.9.

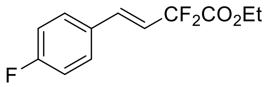


**Ethyl (*E*)-2,2-difluoro-4-(4-fluorophenyl)but-3-enoate (3i).** This compound is known.[3] The product (61.1 mg, 83% yield) was purified with silica gel chromatography (Petroleum ether/Ethyl acetate = 50:1) as colorless liquid. $^1$H NMR (400 MHz, $CDCl_3$) δ 7.46–7.40 (m, 2H), 7.09–7.01 (m, 3H), 6.27–6.17 (m 1H), 4.35 (q, $J$ = 7.2 Hz, 2H), 1.37 (t, $J$ = 7.2 Hz, 3H). $^{19}$F NMR (376 MHz, $CDCl_3$) δ -103.2 (d, $J$ =11.3Hz, 2F), -110.9 (s, 1F). $^{13}$C NMR (101 MHz, $CDCl_3$) δ 163.8 (t, $J$ = 35.0 Hz), 163.4 (d, $J$ = 251.3 Hz), 135.6 (t, $J$ = 9.5 Hz), 130.3–130.2 (m), 129.2 (d, $J$ =8.5 Hz), 118.8–118.2 (m), 115.9 (d, $J$ = 22.0 Hz), 112.6 (t, $J$ = 249.8 Hz), 63.1, 13.9.

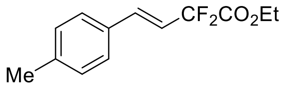


**Ethyl (*E*)-2,2-difluoro-4-(p-tolyl)but-3-enoate (3j).** This compound is known.[3] The product (60.2 mg, 86% yield) was purified with silica gel chromatography (Petroleum

ether/Ethyl acetate = 50:1) as colorless liquid. $^1$H NMR (400 MHz, $CDCl_3$) δ 7.35 (d, *J* =7.6 Hz, 2H), 7.18 (d, *J* = 8.0 Hz, 2H), 7.06 (d, *J* = 16.4 Hz, 1H), 6.32–6.21 (m, 1H), 4.35 (q, *J* = 7.2 Hz, 2H), 2.37 (s, 3H), 1.37 (t, *J* = 7.2 Hz, 3H). $^{19}$F NMR (376 MHz, $CDCl_3$) δ -106.0 (d, *J* =10.9 Hz, 2F). $^{13}$C NMR (101 MHz, $CDCl_3$) δ 163.9 (t, *J* = 35.1 Hz), 139.8, 136.7 (t, *J* = 9.8 Hz), 131.3, 129.5, 127.3, 117.7 (t, *J* = 24.8 Hz), 112.8 (t, *J* = 247.8 Hz), 63.0, 21.2, 13.9.

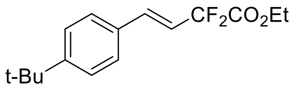


**Ethyl (*E*)-4-(4-(tert-butyl)phenyl)-2,2-difluorobut-3-enoate (3k).** This compound is known.[4] The product (76.6 mg, 90% yield) was purified with silica gel chromatography (Petroleum ether/Ethyl acetate = 50:1) as colorless liquid. $^1$H NMR (400 MHz, $CDCl_3$) δ 7.41 (s, 4H), 7.11–7.05 (m, 1H), 6.34–6.23 (m, 1H), 4.36 (q, *J* = 7.2 Hz, 2H), 1.37 (t, *J* = 7.2 Hz, 3H), 1.34 (s, 9H). $^{19}$F NMR (376 MHz, $CDCl_3$) δ -98.9 (d, *J* =10.5 Hz, 2F). $^{13}$C NMR (101 MHz, $CDCl_3$) δ 163.9 (t, *J* = 35.3 Hz), 153.0, 136.6 (t, *J* = 9.6 Hz), 131.3, 127.2, 125.7, 117.9 (t, *J* = 25.0 Hz), 112.8 (t, *J* = 249.7 Hz), 63.0, 34.7, 31.1, 13.9.

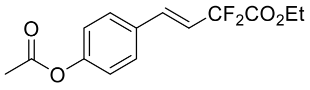


**Ethyl (*E*)-4-(4-acetoxyphenyl)-2,2-difluorobut-3-enoate (3l).** This compound is known.[4] The product (71.5 mg, 84% yield) was purified with silica gel chromatography (Petroleum ether/Ethyl acetate = 10:1) as colorless liquid. $^1$H NMR (400 MHz, $CDCl_3$) δ 7.46 (d, *J* = 8.4 Hz, 2H), 7.10 (d, *J* = 8.4 Hz, 2H), 7.05 (d, *J* = 16.0 Hz, 1H), 6.26 (dt, *J* = 16.0 Hz, 11.4 Hz, 1H), 4.34 (q, *J* = 7.2 Hz, 2H), 2.30 (s, 3H), 1.35 (t, *J* = 7.2 Hz, 3H). $^{19}$F NMR (376 MHz, $CDCl_3$) δ -103.3 (s, 2F). $^{13}$C NMR (101 MHz, $CDCl_3$) δ 169.2, 163.8 (t, *J* = 34.9 Hz), 151.5, 135.8 (t, *J* = 9.5 Hz), 131.8, 128.5, 122.1, 119.0 (t, *J* = 25.1 Hz), 112.6 (t, *J* = 249.9 Hz), 63.1, 21.1, 13.9.

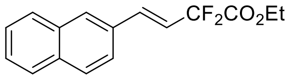


**Ethyl (*E*)-2,2-difluoro-4-(naphthalen-2-yl)but-3-enoate (3m).** This compound is known.[3] The product (49.9 mg, 60% yield) was purified with silica gel chromatography (Petroleum ether/Ethyl acetate = 50:1) as colorless liquid. $^1$H NMR (400 MHz, $CDCl_3$) δ 7.83 (d, *J* = 8.0 Hz, 4H), 7.61 (d, *J* = 8.8 Hz, 1H), 7.51 (q, *J* = 3.2 Hz, 2H), 7.27–7.21 (m, 1H), 6.44 (dt, *J* = 16.0 Hz, 11.6 Hz, 1H), 4.38 (q, *J* = 7.2 Hz, 2H), 1.39 (t, *J* = 7.2 Hz, 3H). $^{19}$F NMR (376 MHz, $CDCl_3$) δ -110.3 (d, *J* = 11.3 Hz, 2F). $^{13}$C NMR (101 MHz, $CDCl_3$) δ 163.9 (t, *J* = 34.9 Hz), 136.9 (t, *J* = 9.5 Hz), 133.8, 133.2, 131.5, 128.8 (d, *J* = 1.1 Hz), 128.6, 128.3, 127.7, 126.9, 126.6, 123.2, 118.9 (t, *J* = 25.1 Hz), 112.8 (t, *J* = 249.8 Hz), 63.1, 13.9.

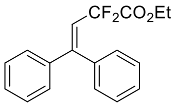


**Ethyl 2,2-difluoro-4,4-diphenylbut-3-enoate (3n).** This compound is known.[3] The product (70.6 mg, 78% yield) was purified with silica gel chromatography (Petroleum ether/Ethyl acetate = 50:1) as colorless liquid. $^1$H NMR (400 MHz, $CDCl_3$) δ 7.40–7.35 (m, 3H), 7.35–7.29 (m, 3H), 7.29–7.24 (m, 2H), 7.23–7.18 (m, 2H), 6.28 (t, *J* = 11.8 Hz, 1H), 3.91 (q, *J* = 7.2 Hz, 2H), 1.17 (t, *J* = 7.2 Hz, 3H). $^{19}$F NMR (376 MHz, $CDCl_3$) δ -98.2 (d, *J* = 11.7 Hz, 2F). $^{13}$C NMR (101 MHz, $CDCl_3$) δ 163.4 (t, *J* = 34.1 Hz), 150.9 (t, *J* = 9.5 Hz), 140.4, 137.0, 129.8 (t, *J* = 1.9 Hz), 129.0, 128.5, 128.3, 127.9, 127.8, 119.4 (t, *J* = 28.5 Hz), 112.5 (t, *J* = 246.1 Hz), 62.7, 13.6.

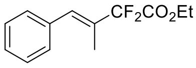


**Ethyl (*E*)-2,2-difluoro-3-methyl-4-phenylbut-3-enoate** (3o). This compound is known.[3] The product (42.5 mg, 59% yield, Z:E = 1:8.8) was purified with silica gel chromatography (Petroleum ether/Ethyl acetate = 100:1) as colorless liquid. $^1$H NMR (400 MHz, $CDCl_3$) δ 7.43–7.36 (m, 2H), 7.34–7.28 (m, 3H), 6.96 (s, 1H), 4.37 (q, *J* = 7.2 Hz, 2H), 2.00 (s, 3H), 1.37 (t, *J* = 7.2 Hz, 3H). $^{19}$F NMR (376 MHz, $CDCl_3$) δ

-106.7 (s, 2F). $^{13}$C NMR (101 MHz, $CDCl_3$) δ 164.0 (t, *J* = 35.4 Hz), 135.1, 130.9 (t, *J* = 9.3 Hz), 129.1, 128.3, 127.9, 114.3 (t, *J* = 252.6 Hz), 63.0, 13.9, 12.5 (t, *J* = 2.8 Hz).

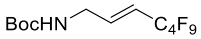


**Tert-butyl (*E*)-(4,4,5,5,6,6,7,7,7-nonafluorohept-2-en-1-yl)carbamate (3p).** The product (66.4 mg, 59% yield) was purified with silica gel chromatography (Petroleum ether/Ethyl acetate = 50:1) as colorless liquid. $^1$H NMR (400 MHz, $CDCl_3$) δ 6.45–6.36 (m, 1H), 5.80–5.69 (m, 1H), 4.80 (s, 1H), 3.91 (s, 2H), 1.44 (s, 9H). $^{19}$F NMR (376 MHz, $CDCl_3$) δ -88.5 (m, 3F), -119.1 (m, 2F), -131.7 (m, 2F), -133.1 (m, 2F). $^{13}$C NMR (101 MHz, $CDCl_3$) δ 155.5, 139.6 (t, *J* = 8.1 Hz), 120.2–107.0 (m), 117.2 (t, *J* = 23.4 Hz), 80.1, 41.1, 28.2. MS (ESI): m/z (%) 398 (M+ $Na^+$), 276 (100). HRMS (ESI): Calculated for $C_{12}H_{14}O_2NF_9Na$ (M+$Na^+$): 398.0773; Found: 398.0770.

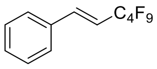


**(*E*)-(3,3,4,4,5,5,6,6,6-nonafluorohex-1-en-1-yl)benzene (3q).** This compound is known.[5] The product (60.6 mg, 63% yield) was purified with silica gel chromatography (Petroleum ether) as colorless liquid. $^1$H NMR (400 MHz, $CDCl_3$) δ 7.51–7.46 (m, 2H), 7.43–7.38 (m, 3H), 7.22–7.15 (m, 1H), 6.26–6.15 (m, 1H). $^{19}$F NMR (376 MHz, $CDCl_3$) δ -81.2 (m, 3F), -111.5 (m, 2F), -124.3 (m, 2F), -125.9(m, 2F). $^{13}$C NMR (101 MHz, $CDCl_3$) δ 139.8 (t, *J* = 9.6 Hz), 133.5, 130.2, 129.0, 127.6, 120.0–112.0 (m), 114.2 (t, *J* = 23.0 Hz).




**Tert-butyl (*E*)-(4,4,5,5,6,6,7,7,8,8,9,9,9-tridecafluoronon-2-en-1-yl)carbamate (3r).** The product (102.6 mg, 72% yield) was purified with silica gel chromatography (Petroleum ether/Ethyl acetate = 15:1) as colorless liquid. $^1$H NMR (400 MHz, $CDCl_3$) δ 6.40 (s, 1H), 5.85–5.65 (m, 1H), 5.10–4.85 (m, 1H), 3.89 (s, 2H), 1.52–1.36 (m, 9H). $^{19}$F NMR (376 MHz, $CDCl_3$) δ -81.0 (m, 3F), -111.7 (m, 2F), -121.8 (s, 2F), -123.1 (s,

2F), -123.6 (s, 2F), -126.4 (m, 2F). $^{13}$C NMR (101 MHz, $CDCl_3$) δ 155.5, 139.6 (t, $J$ = 8.8 Hz), 120.2–110.0 (m), 117.3 (t, $J$ = 22.4 Hz), 80.1, 41.1, 28.2. MS (ESI): m/z (%) 398 (M+ $Na^+$), 276 (100). HRMS (ESI): Calculated for $C_{14}H_{14}O_2NF_{13}Na$ (M+$Na^+$): 498.0709; Found: 498.0707.

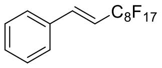


**(*E*)-(3,3,4,4,5,5,6,6,7,7,8,8,9,9,10,10,10-heptadecafluorodec-1-en-1-yl)benzene (3s).** This compound is known.[5] The product (90.3 mg, 58% yield) was purified with silica gel chromatography (Petroleum ether) as colorless liquid. $^1$H NMR (400 MHz, $CDCl_3$) δ 7.52–7.42 (m, 2H), 7.45–7.35 (m, 3H), 7.20–7.10 (m, 1H), 6.26–6.12 (m, 1H). $^{19}$F NMR (376 MHz, $CDCl_3$) δ -81.1 (m, 3F), -111.3 (m, 2F), -121.6 (s, 2F), -122.1 (s, 4F), -122.9 (s, 2F), -123.4 (s, 2F). $^{13}$C NMR (101 MHz, $CDCl_3$) δ 139.7 (t, $J$ = 9.2 Hz), 133.6, 130.2, 129.0, 127.6, 120.0–110.0 (m), 114.4 (t, $J$ = 23.5 Hz).

## 3. Mechanism study.

### 3.1 Addition of radical and SET inhibitors:

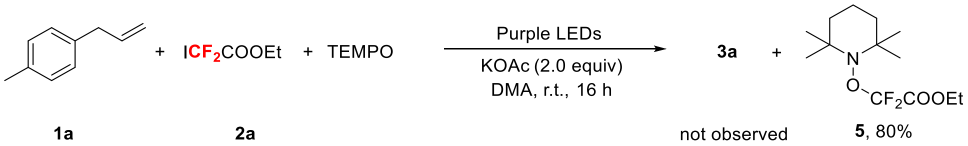


When the radical scavenger TEMPO (2,2,6,6-tetromethyl-1-piperidinyloxy, 1.0 equiv.) was added under standard conditions, 80% product (4) was observed.

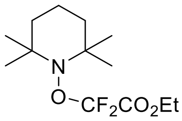


**Ethyl 2,2-difluoro-2-((2,2,6,6-tetramethylpiperidin-1-yl)oxy)acetate (5).** This compound is known.[4] The product (66.9 mg, 80% yield) was purified with silica gel chromatography (Petroleum ether/Ethyl acetate = 50:1) as colorless liquid. $^1$H NMR (400 MHz, $CDCl_3$) δ 4.35 (q, $J$ = 7.2 Hz, 2H), 1.61–1.52 (m, 5H), 1.37 (t, $J$ = 7.2 Hz,

4H), 1.19 (s, 6H), 1.16 (s, 6H). $^{19}$F NMR (376 MHz, $CDCl_3$) δ -73.5 (t, *J* = 2.3 Hz, 2F). $^{13}$C NMR (101 MHz, $CDCl_3$) δ 160.7, 115.4, 63.0, 61.4, 40.1, 33.4 (t, *J* = 4.4 Hz), 20.7, 16.9, 13.9.

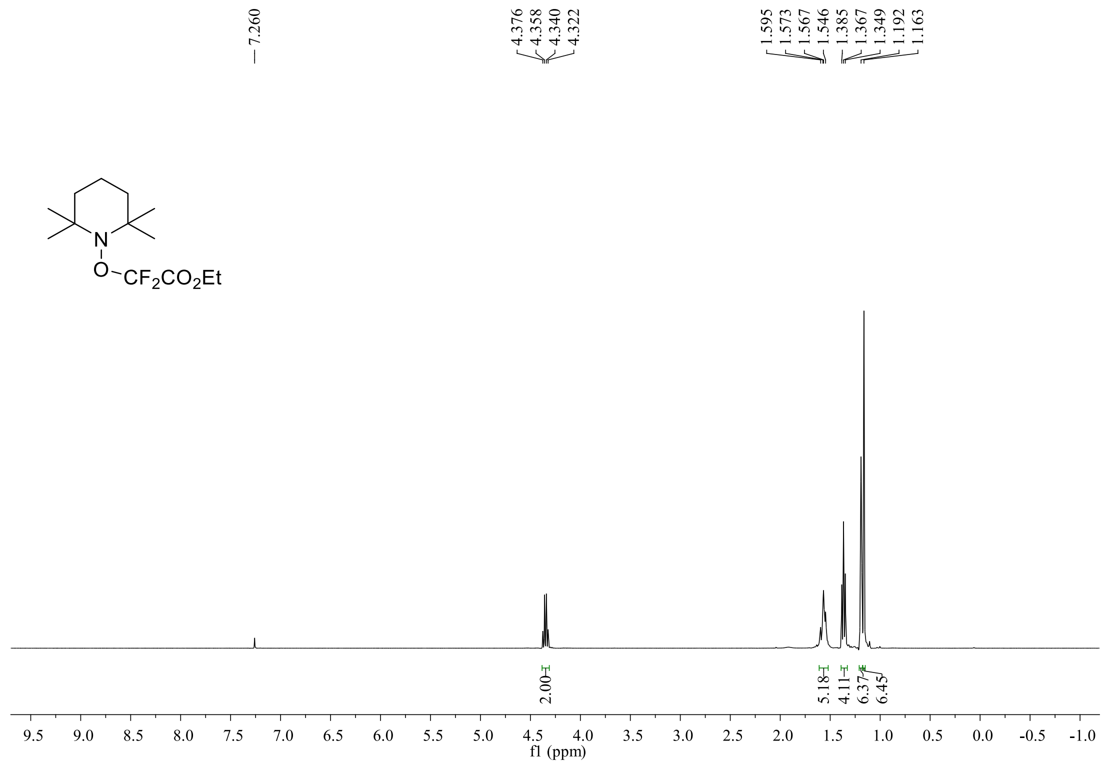

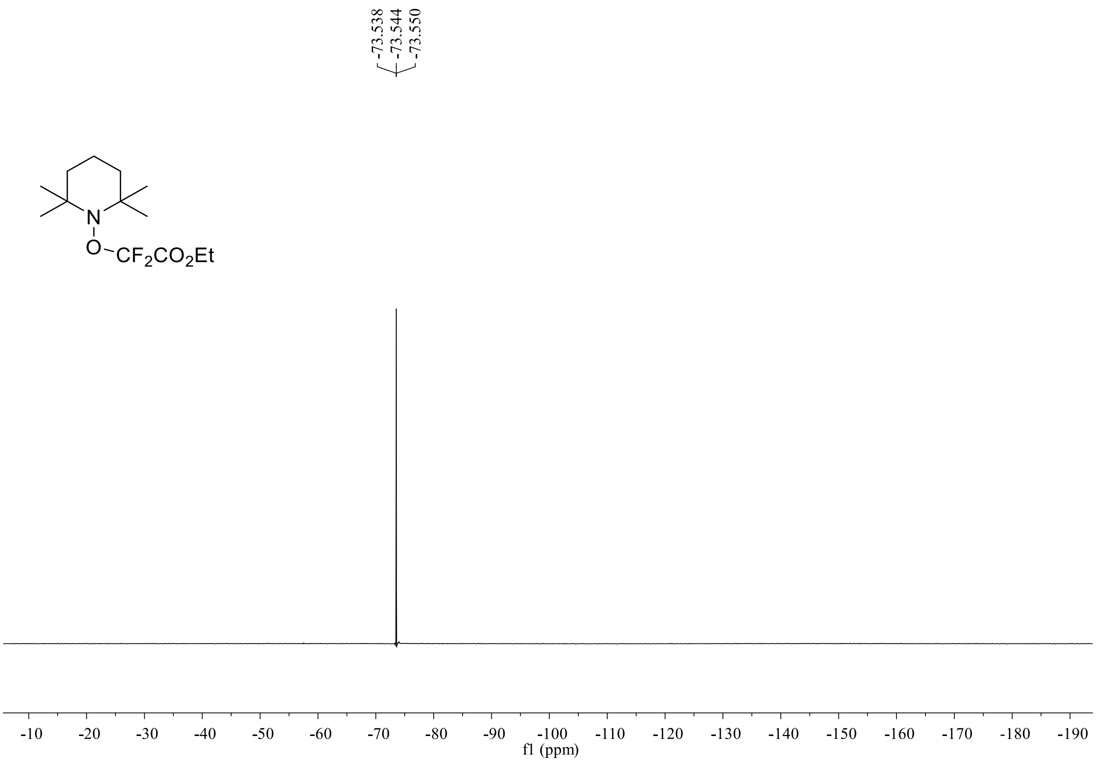
-73.538
-73.544
-73.550
N
O
CF2CO2Et
-10 -20 -30 -40 -50 -60 -70 -80 -90 -100 -110 -120 -130 -140 -150 -160 -170 -180 -190
f1 (ppm)

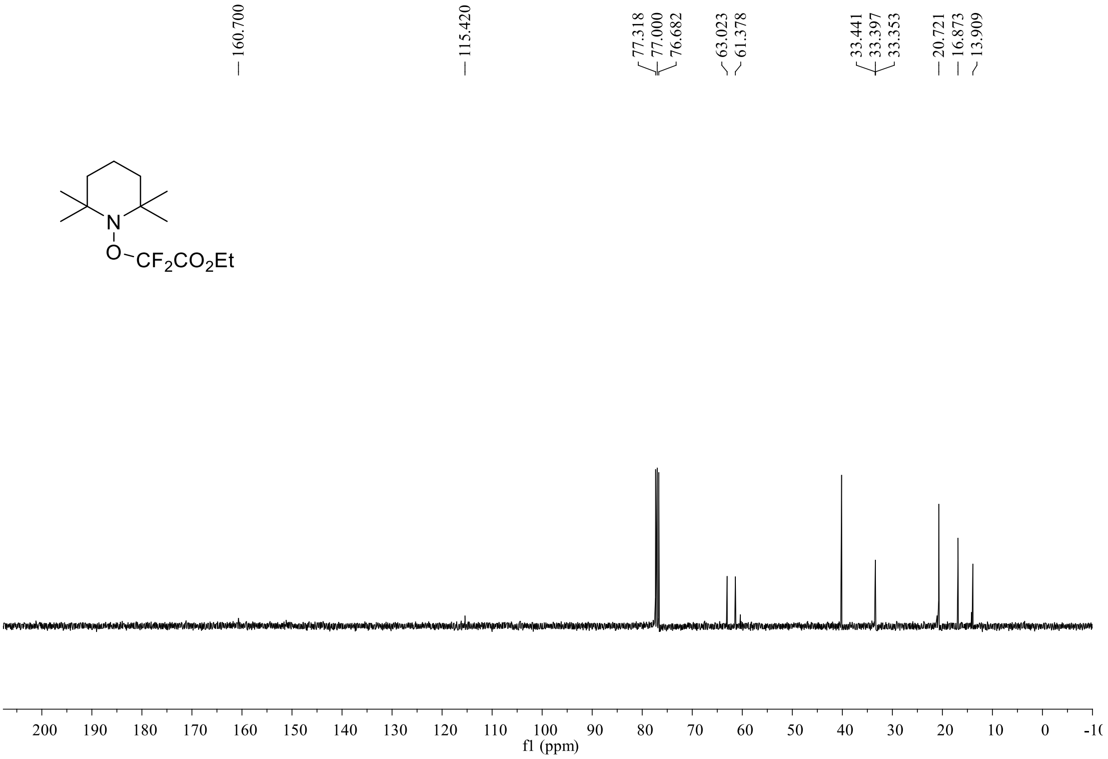
160.700
115.420
77.318
77.000
76.682
63.023
61.378
33.441
33.397
33.353
20.721
16.873
13.909
N
O
CF2CO2Et
200 190 180 170 160 150 140 130 120 110 100 90 80 70 60 50 40 30 20 10 0
f1 (ppm)

## 3.2 Trapping of intermediates:

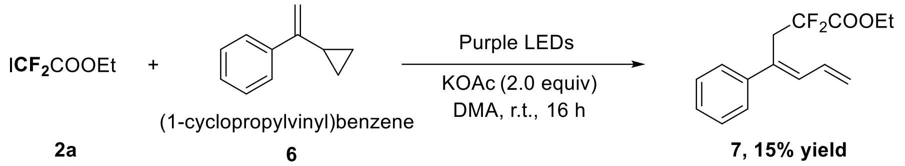


**Typical procedure:** To a 25 mL of Schlenk tube equipped with a Teflon septum were added KOAc (0.6 mmol, 2.0 equiv.) under Ar, followed by DMA (2.0 mL). $ICF_2COOEt$ **(2a)** (0.45 mmol, 1.5 equiv.) and (1-cyclopropylvinyl)benzene **(6)** (0.45 mmol, 1.5 equiv.) were added subsequently. After stirring under purple light for 16 hours, the mixture was purified with silica gel chromatography to provide product **(7)**. Ethyl(*E*)-2,2-difluoro-4-phenylhepta-4,6-dienoate**(7)**. The product (11.98 mg, 15% yield) was purified with silica gel chromatography (Petroleum ether/Ethyl acetate = 50:1) as colorless liquid. $^1$H NMR (400 MHz, $CDCl_3$) δ 7.39–7.29 (m, 5H), 6.76–6.66 (m, 1H), 6.56–6.51 (m, 1H), 5.45–5.38 (m, 1H), 5.35–5.31(m, 1H), 3.94 (q, $J$ = 7.2 Hz, 2H), 3.46 (t, $J$ = 15.2 Hz, 2H), 1.17 (t, $J$ = 7.2 Hz, 3H). MS (EI): m/z (%) 266, 129 (100). HRMS (EI): Calculated for $C_{15}H_{16}O_2F_2$ (M): 266.1118; Found: 266.1116.

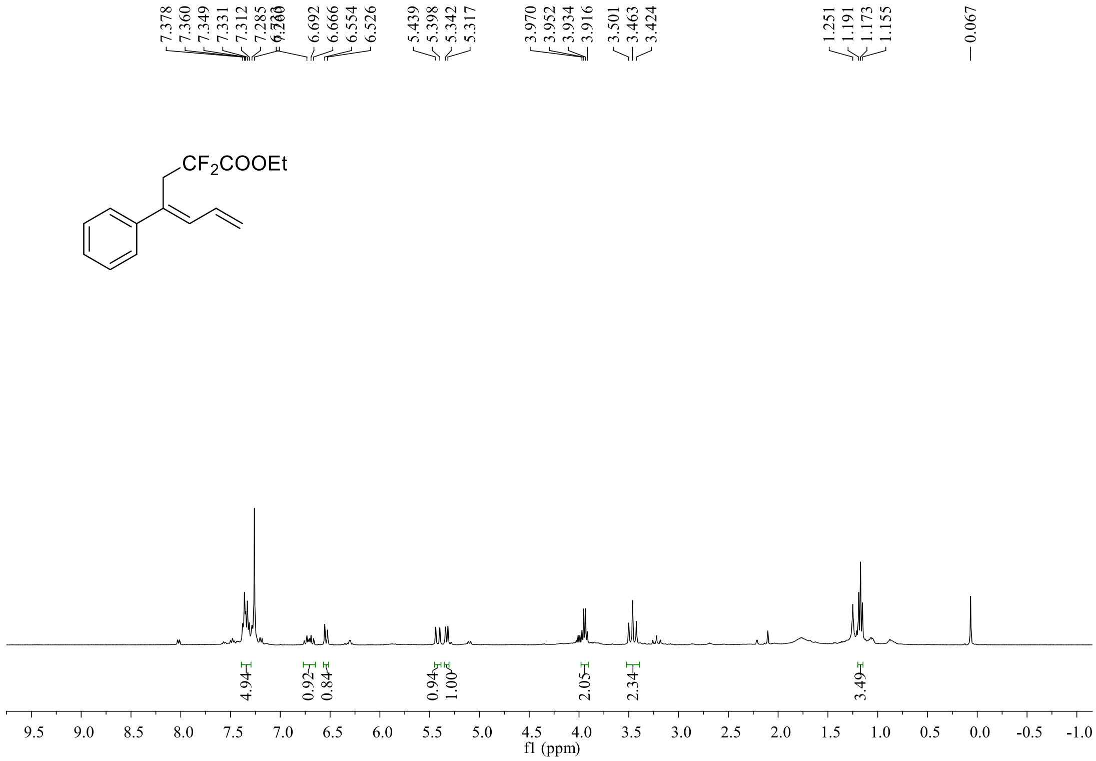

### 3.3 Control experiment.

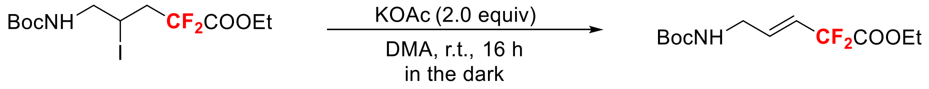


4a 3a

**Typical procedure:** To a 25 mL of Schlenk tube equipped with a Teflon septum were added KOAc (0.6 mmol, 2.0 equiv.) under Ar, followed by DMA (2 mL), Ethyl 5-((tert-butoxycarbonyl)amino)-2,2-difluoro-4-iodopentanoate (**4a**) (0.3 mmol, 1.0 equiv.) was added subsequently. After stirring for 16 hours in the dark, the mixture detected by F-NMR (yield 75% *Z/E*=1:17.5).

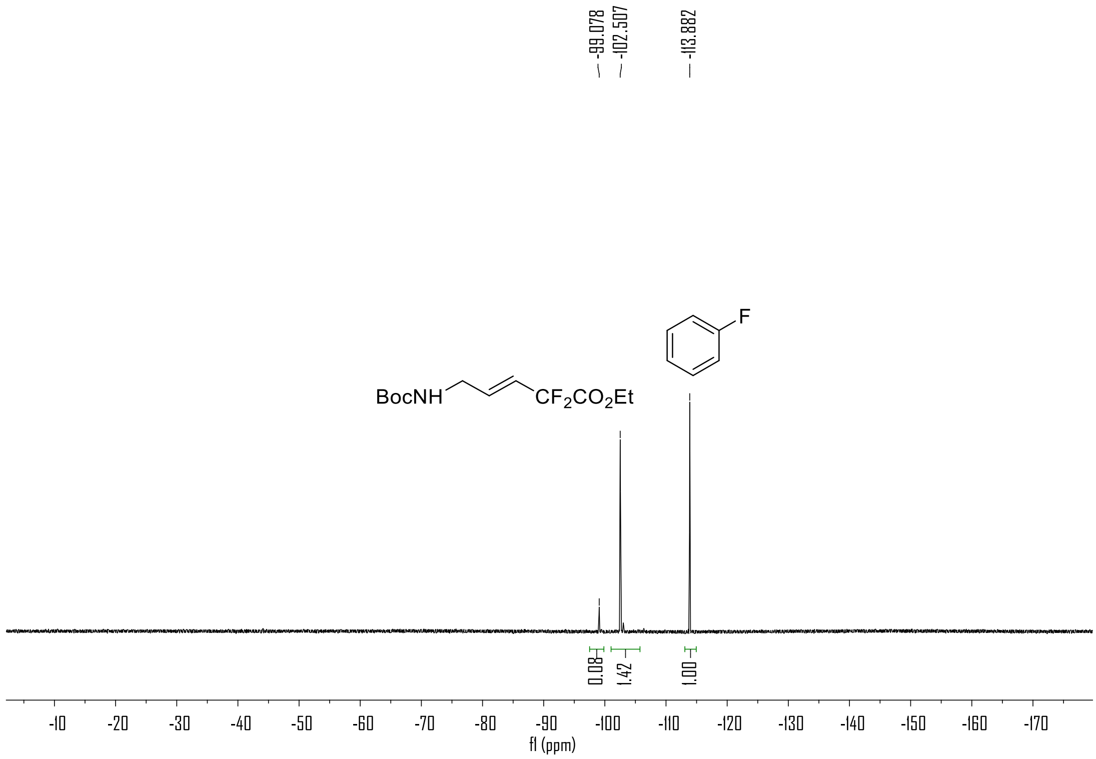


### 3.4 UV-vis spectroscopic measurement.

Solution 1: KOAc (58.9 mg, 0.6 mmol) and **2a** (60 µL, 0.4 mmol) was added in DMA (4.0 mL). The mixture was stirred for 20 minutes, filtered before use.

Solution 2: **2a** (60 µL, 0.4 mmol) was added in DMA (4.0 mL). The mixture was stirred for 20 minutes, filtered before use.

Solution 3: KOAc (58.9 mg, 0.6 mmol) was added in DMA (4.0 mL). The mixture was stirred for 20 minutes, filtered before use.

Solution 4: **2a** (60 μL, 0.4 mmol) was added in Cyclohexane (4.0 mL). The mixture was stirred for 20 minutes, filtered before use.

Performed on UV visible spectrophotometer, recorded in 1cm path quartz cuvettes using T6 Xinyue UV-visible spectrophotometer (PERSEE™), pure DMA as blank sample.

| λ (nm) \ A | 2a + KOAc +DMA $A_1$ | 2a + DMA $A_2$ | KOAc + DMA $A_3$ | 2a + cyclohexane $A_4$ |
|---|---|---|---|---|
| 330 | 3.09 | 3.092 | 0.098 | 2.197 |
| 340 | 3.142 | 3.119 | 0.041 | 1.697 |
| 350 | 3.367 | 3.333 | 0.019 | 0.975 |
| 360 | 3.341 | 3.593 | 0.012 | 0.504 |
| 370 | 2.616 | 3.621 | 0.01 | 0.272 |
| 380 | 2.459 | 3.662 | 0.009 | 0.145 |
| 390 | 1.845 | 2.601 | 0.007 | 0.082 |
| 400 | 1.034 | 2.653 | 0.005 | 0.055 |
| 410 | 0.558 | 2.54 | 0.006 | 0.042 |
| 420 | 0.324 | 2.054 | 0.006 | 0.039 |
| 430 | 0.196 | 1.55 | 0.007 | 0.046 |
| 440 | 0.132 | 1.143 | 0.007 | 0.064 |
| 450 | 0.092 | 0.804 | 0.007 | 0.097 |
| 460 | 0.065 | 0.558 | 0.007 | 0.155 |
| 470 | 0.047 | 0.361 | 0.006 | 0.222 |
| 480 | 0.033 | 0.239 | 0.006 | 0.312 |
| 490 | 0.026 | 0.164 | 0.007 | 0.425 |
| 500 | 0.021 | 0.123 | 0.007 | 0.524 |
| 510 | 0.018 | 0.103 | 0.007 | 0.601 |
| 520 | 0.016 | 0.093 | 0.006 | 0.633 |
| 530 | 0.016 | 0.087 | 0.007 | 0.619 |
| 540 | 0.016 | 0.081 | 0.007 | 0.568 |
| 550 | 0.015 | 0.074 | 0.007 | 0.484 |
| 560 | 0.015 | 0.065 | 0.006 | 0.387 |
| 570 | 0.014 | 0.056 | 0.006 | 0.297 |
| 580 | 0.014 | 0.046 | 0.007 | 0.214 |
| 590 | 0.013 | 0.039 | 0.006 | 0.154 |
| 600 | 0.013 | 0.031 | 0.007 | 0.109 |
| 610 | 0.012 | 0.026 | 0.007 | 0.079 |

| | | | | |
|---|---|---|---|---|
| 620 | 0.012 | 0.021 | 0.007 | 0.062 |
| 630 | 0.012 | 0.018 | 0.007 | 0.051 |
| 640 | 0.013 | 0.015 | 0.007 | 0.047 |
| 650 | 0.012 | 0.018 | 0.007 | 0.044 |
| 660 | 0.012 | 0.015 | 0.007 | 0.042 |

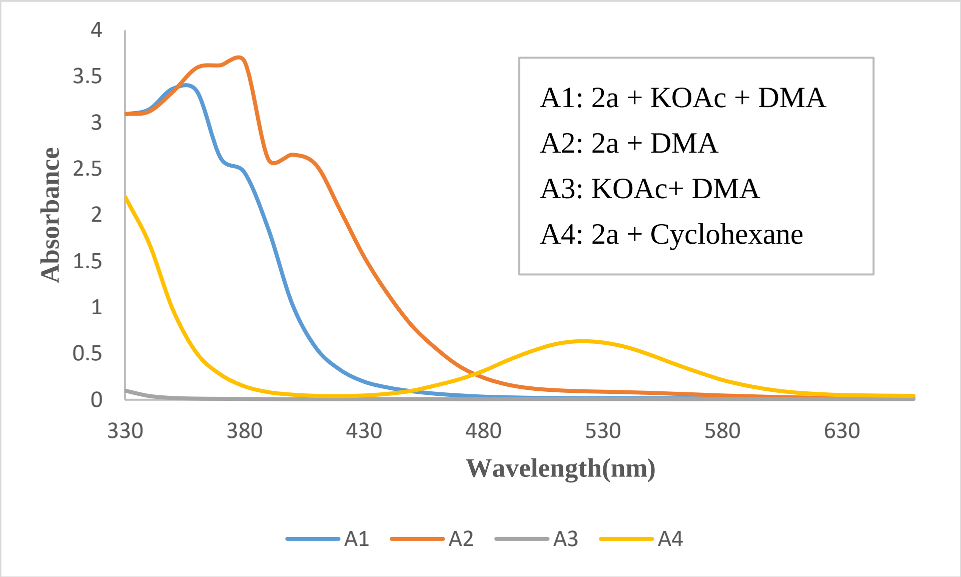


**3.5 Stoichiometry of the weak intermolecular interaction.**

The stoichiometry of the EDA complexes was calculated using the Job´s plot method (P. Job, *Ann. Chim.*, **1928**, *9*, 113.). The Job’s plot of the EDA complex between DMA and ethyl difluoroiodoacetate **(2a)** was calculated measuring the absorption of cyclohexane solutions at 420 nm with different donor/acceptor ratios with constant concentration (0.12 M) of the two components. The absorbance values were plotted against the molar fraction (%) of **2a**. The Job´s plot analysis of the EDA complex between DMA and **2a** showed a maximal absorbance at 50% molar fraction of **2a**.

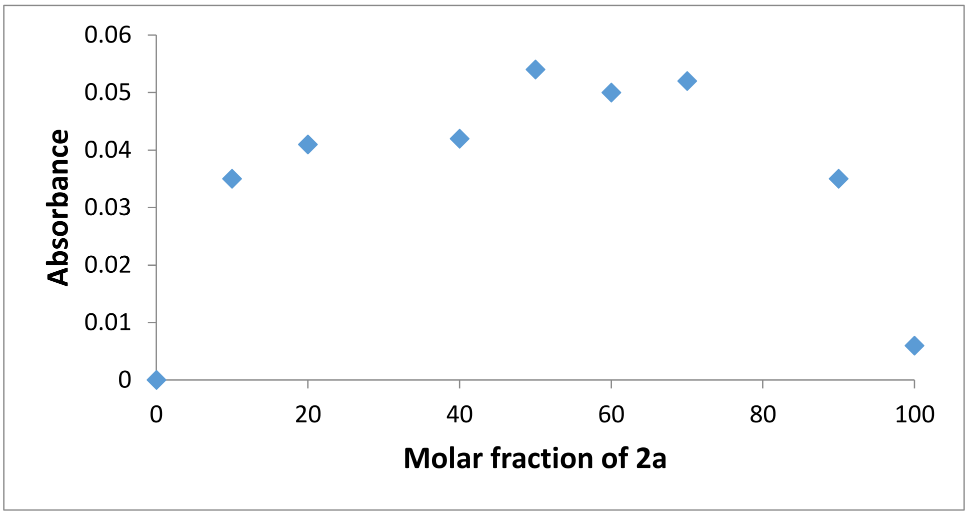


## 4. References:

**[1]** E. Zhu, X-X. Liu, A-J. Wang, T. Mao, L. Zhao, X. Zhang and C-Y. He, *Chem. Commun.*, **2019**, *55*, 12259.

**[2]** M-L. Ke, Q. Feng, K. Yang and Q-L. Song, *Org. Chem. Front.*, **2016**, *3*, 150.

**[3]** X-Y. Wang, S. Zhao, J. Liu, D-S. Zhu, M-J. Guo, X-G. Tang, G-W. Wang, *Org. Lett.*, **2017**, *19*, 4187.

**[4]** L. Zhao, Y. Huang, Z. Wang, E. Zhu, T. Mao, J. Jia, J. Gu, X.-F. Li and C.-Y. He, *Org. Lett.*, **2019**, *21*, 6705.

**[5]** D-S. Lee, E-J. Cho, *Org. Biomol. Chem.*, **2019**, *17*, 4317.

## 5. Copies of NMR spectras of 3.

**Ethyl (*E*)-5-((tert-butoxycarbonyl)amino)-2,2-difluoropent-3-enoate (3a).**

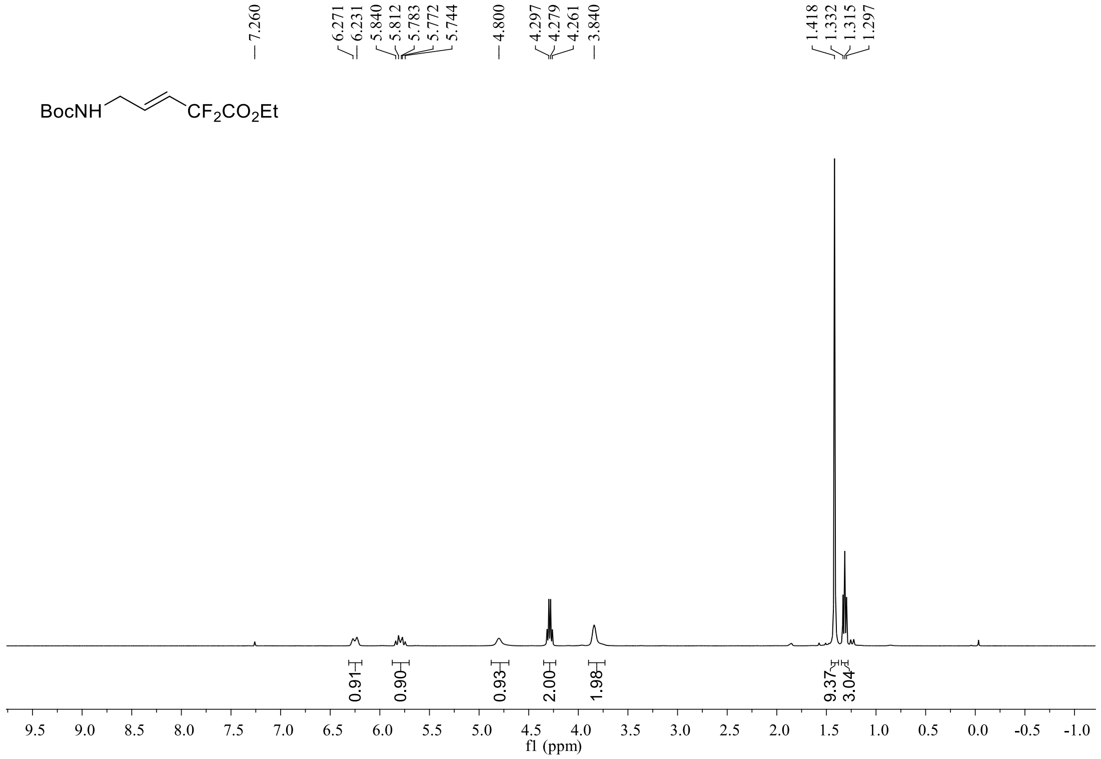

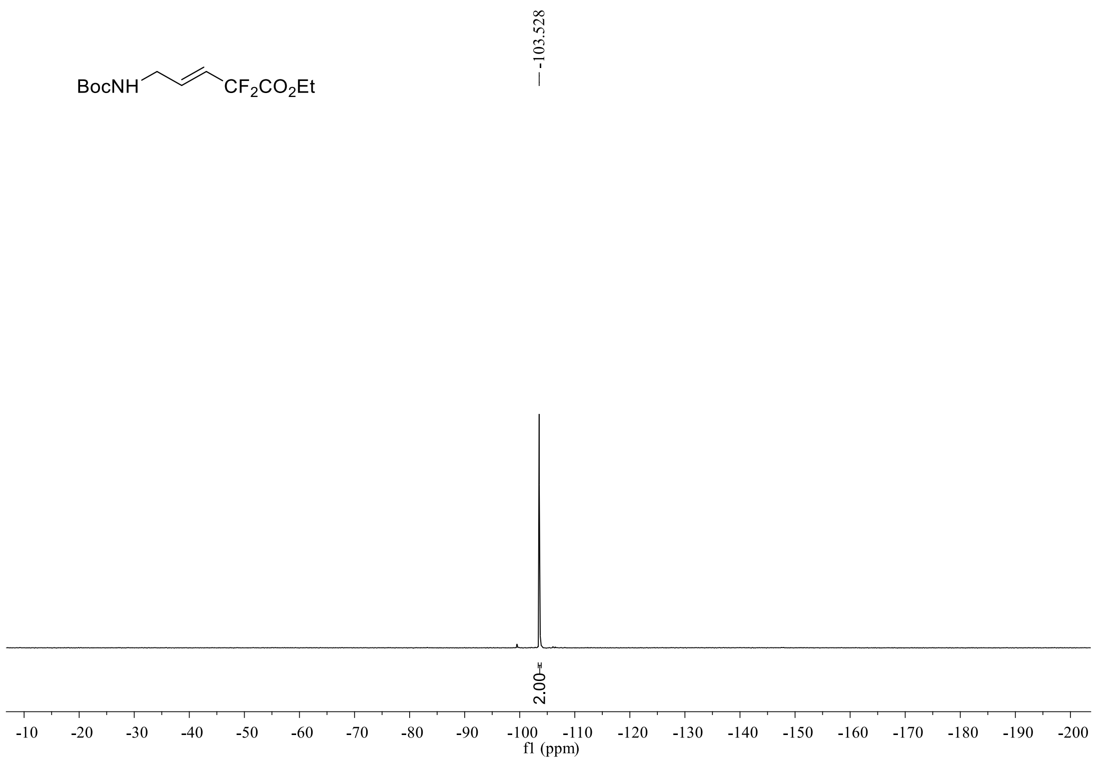

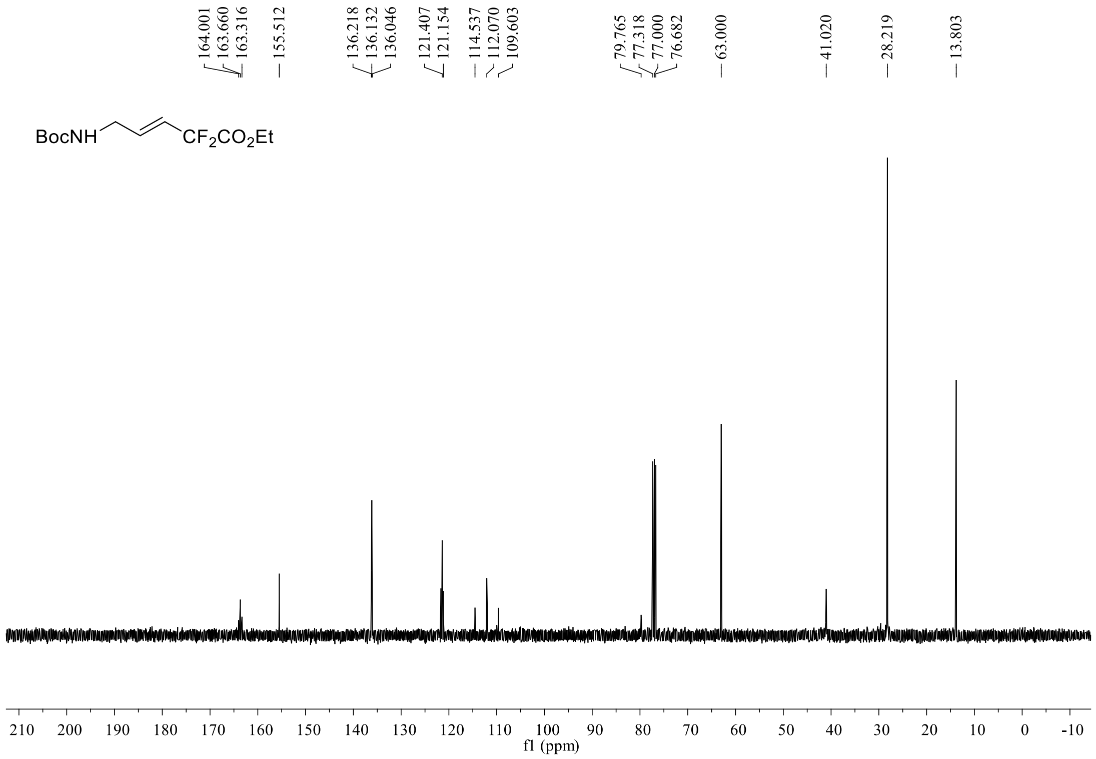

**Ethyl (*E*)-2,2-difluorodec-3-enoate (3b).**

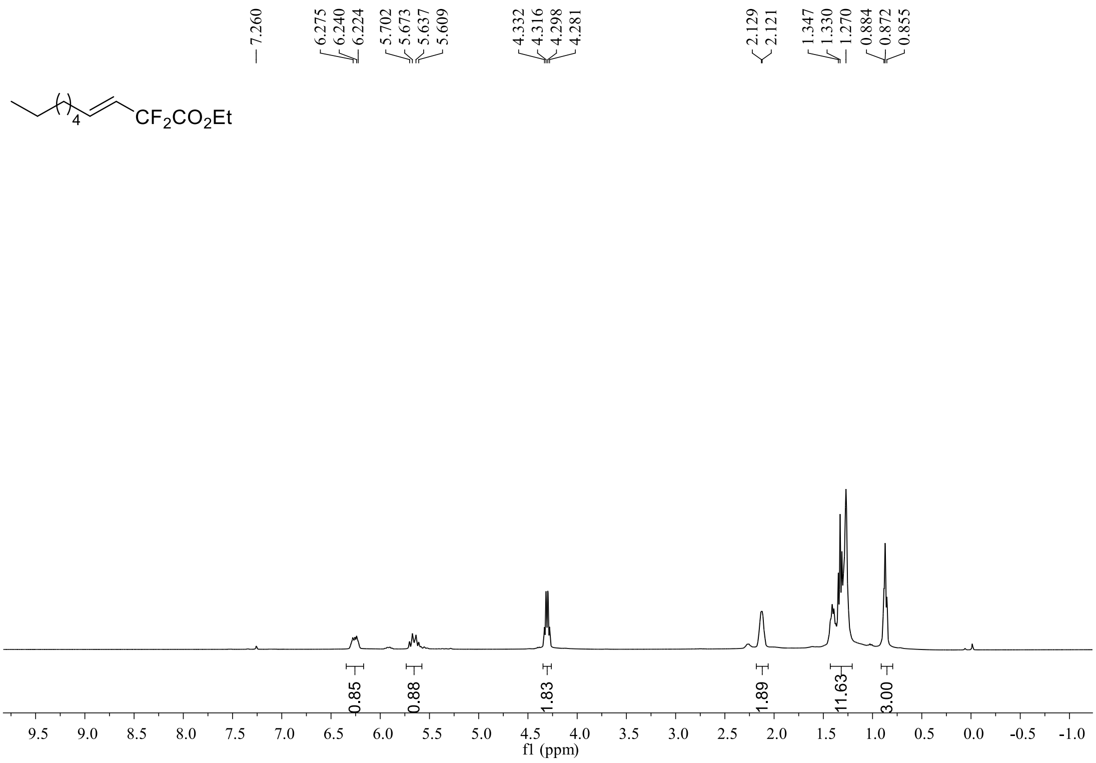

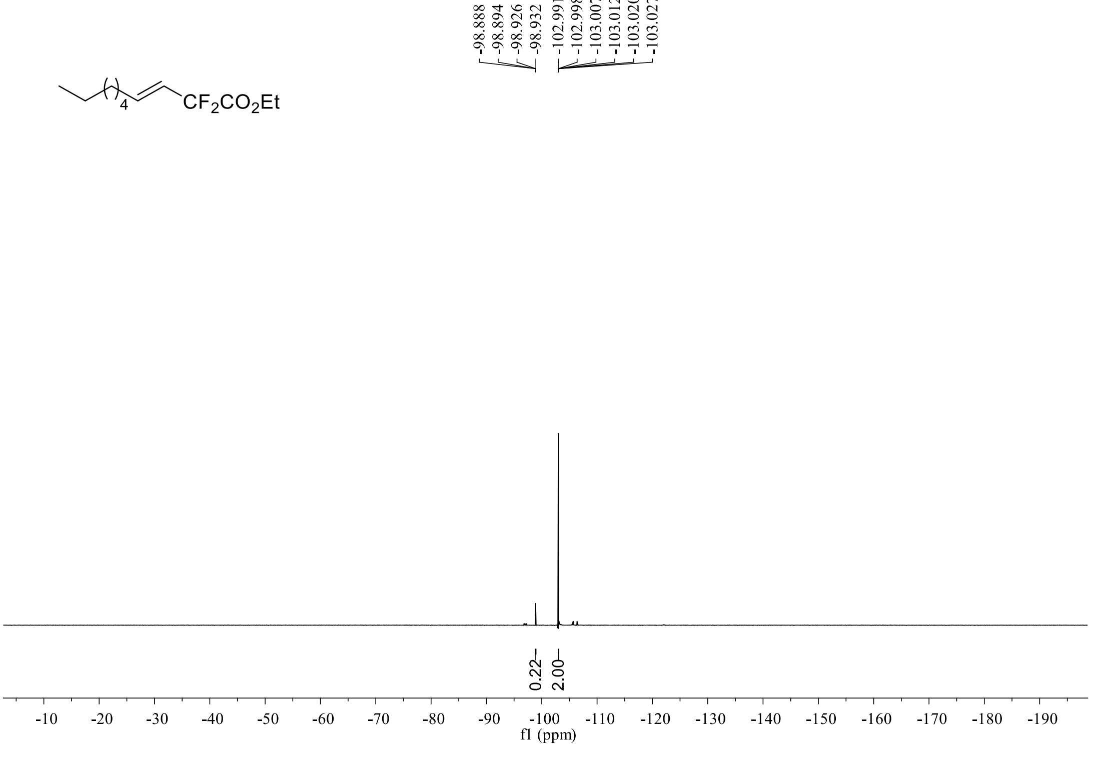

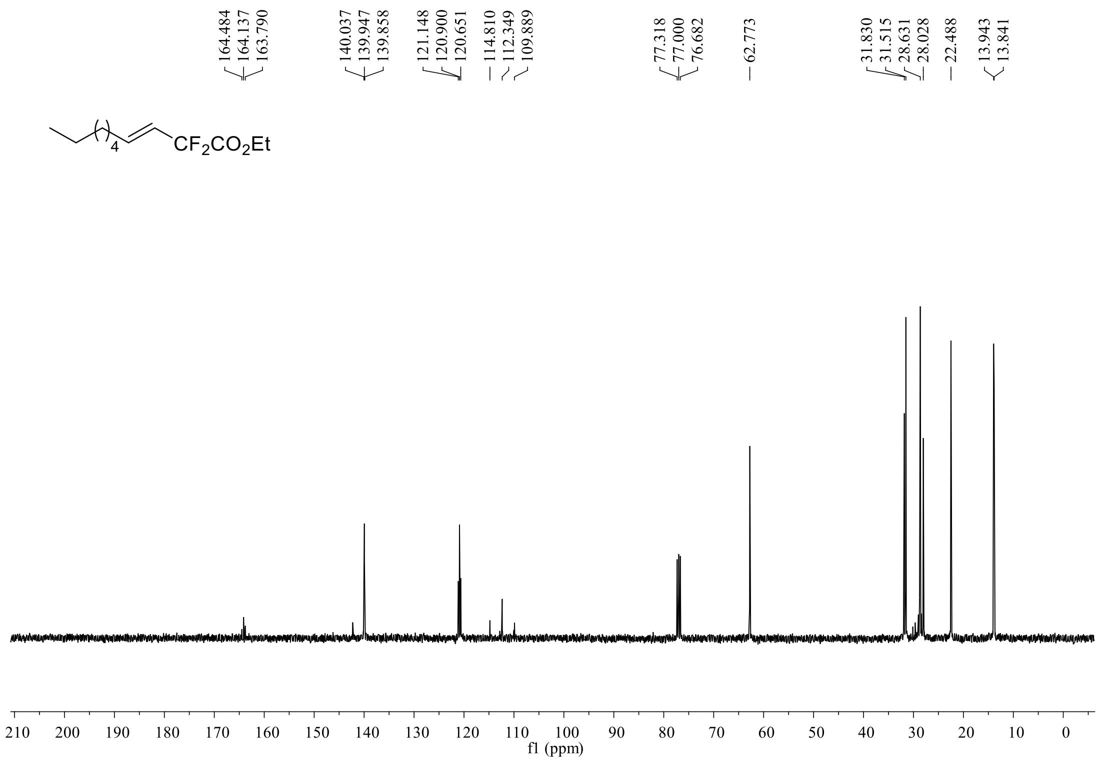


**Ethyl (*E*)-5-acetoxy-2,2-difluoropent-3-enoate (3c).**

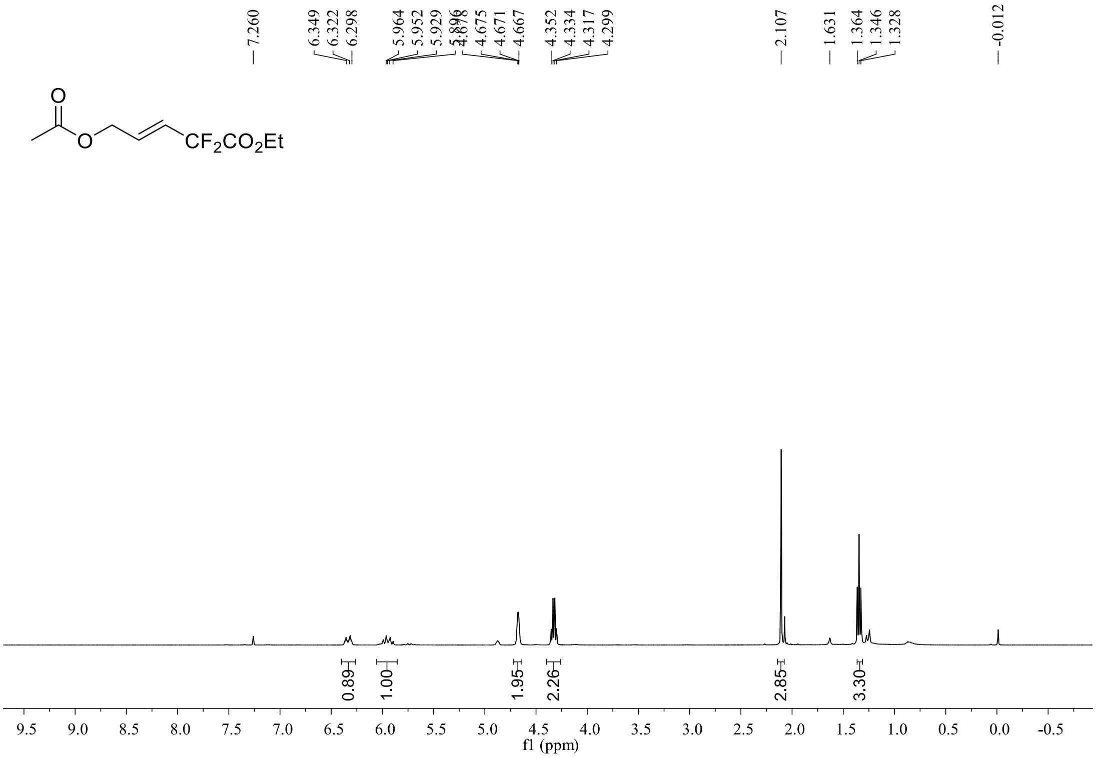
7.260
6.349
6.322
6.298
5.964
5.952
5.929
4.675
4.671
4.667
4.352
4.334
4.317
4.299
2.107
1.631
1.364
1.346
1.328
-0.012
CF2CO2Et
0.89
1.00
1.95
2.26
2.85
3.30
f1 (ppm)

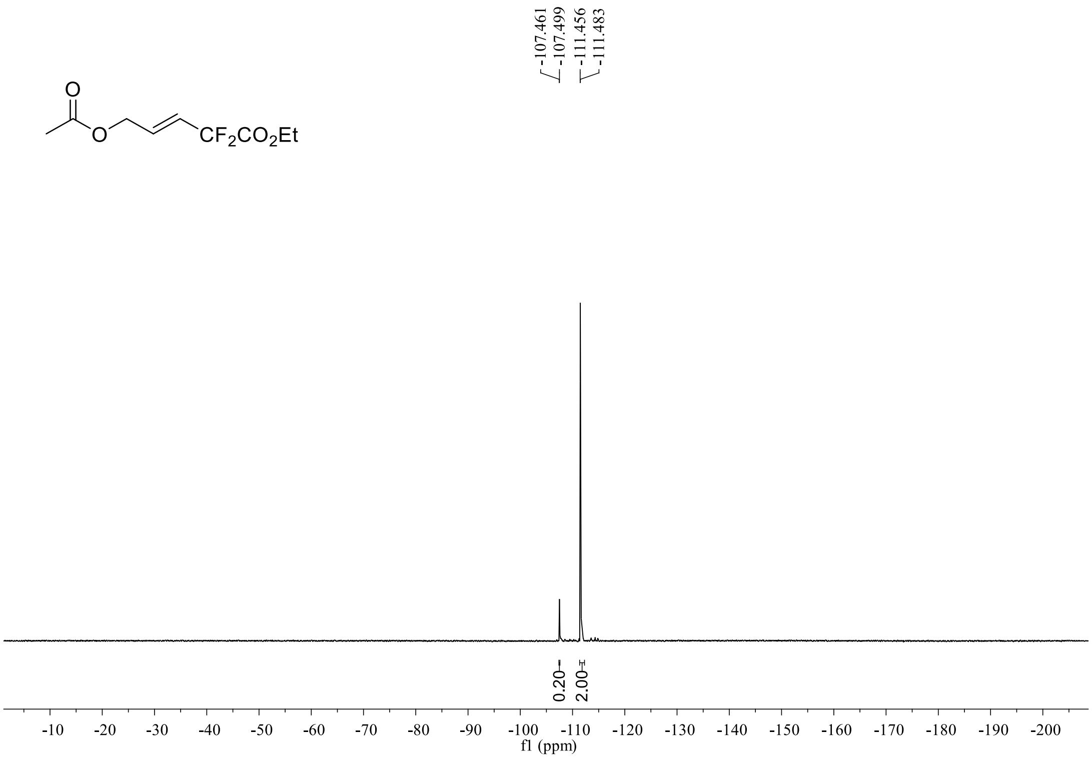
-107.461
-107.499
-111.456
-111.483
CF2CO2Et
0.20
2.00
f1 (ppm)

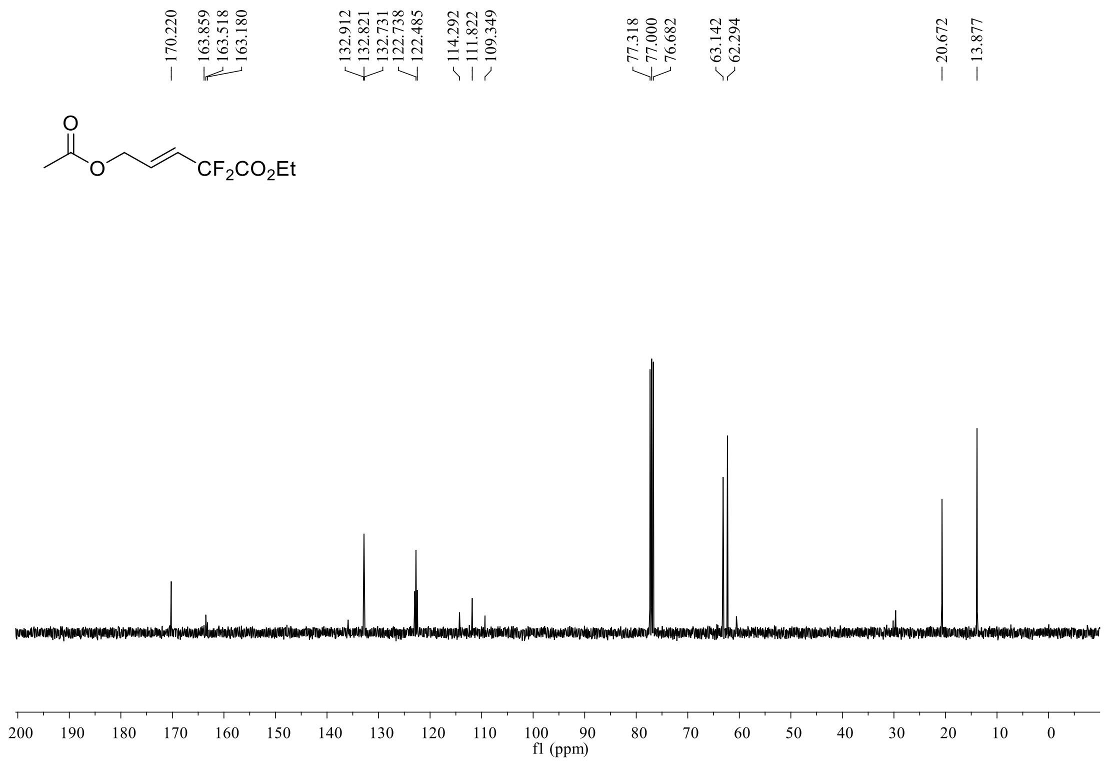


**Ethyl (*E*)-5-ethoxy-2,2-difluoropent-3-enoate (3d).**

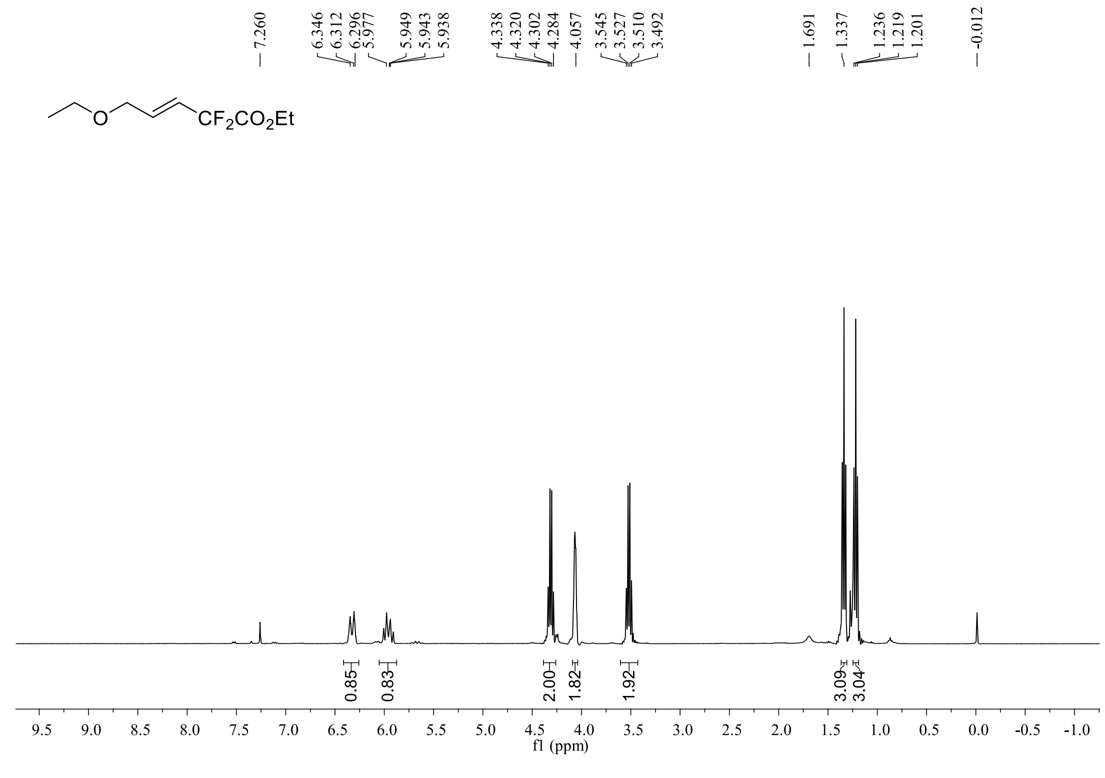

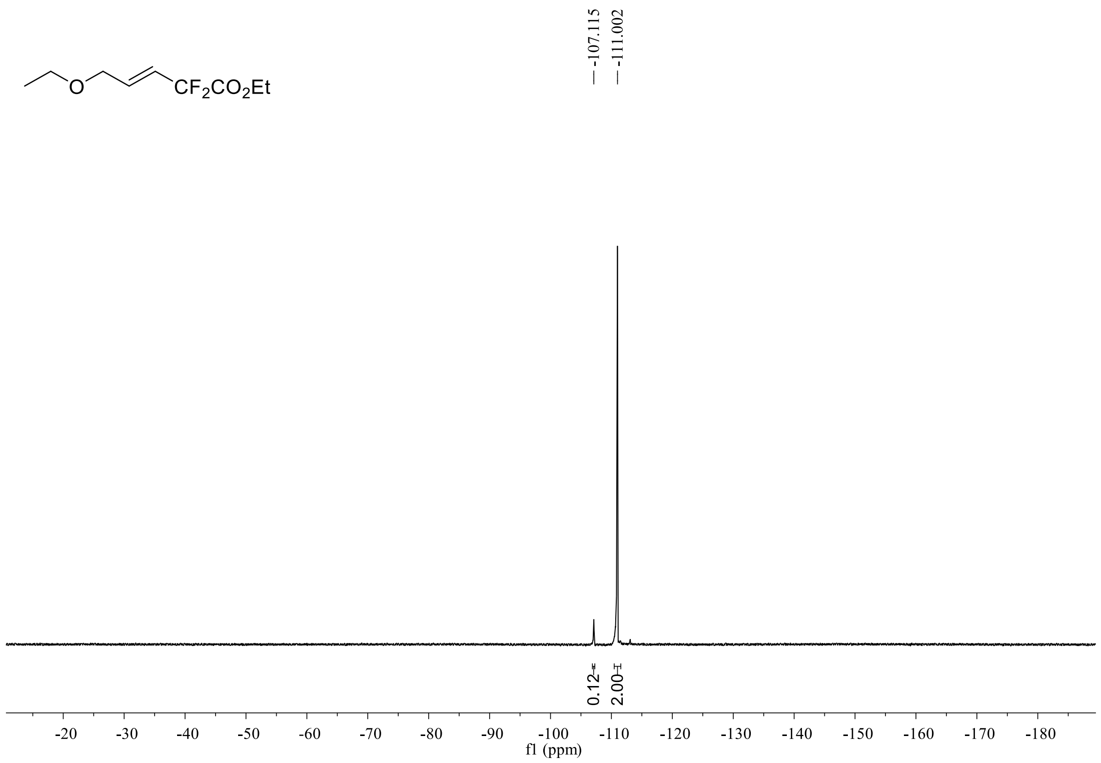
CF2CO2Et
-107.115
-111.002
0.12
2.00
f1 (ppm)

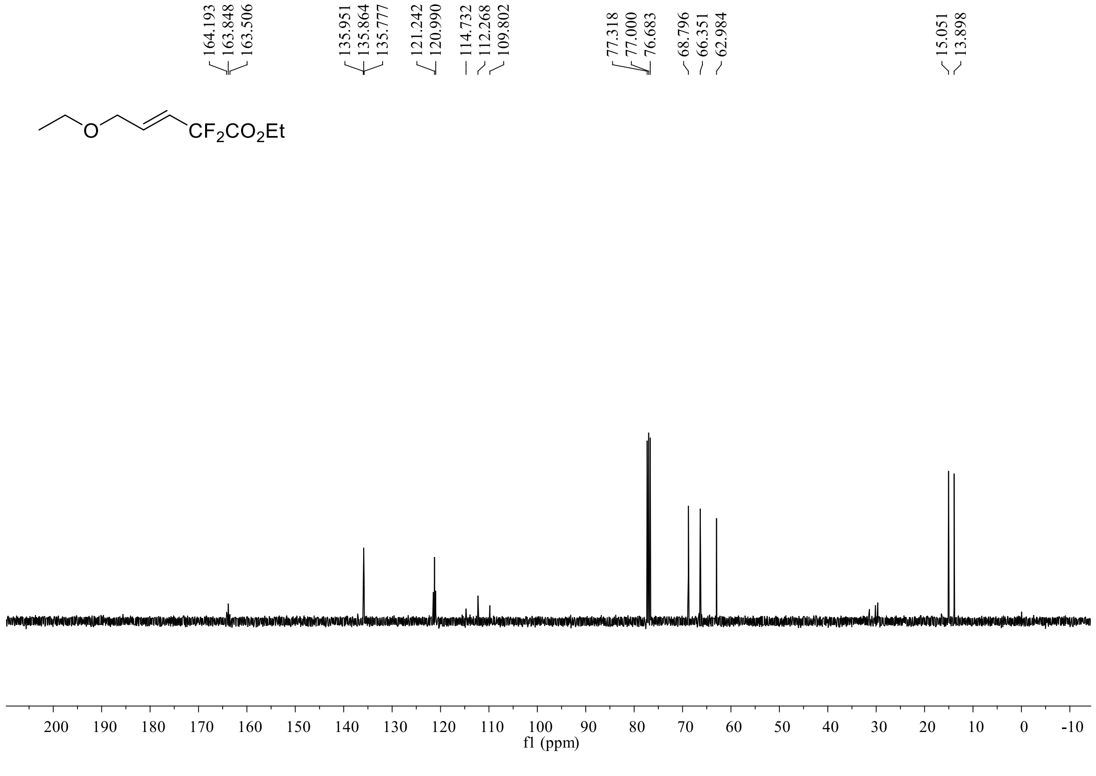
CF2CO2Et
164.193
163.848
163.506
135.951
135.864
135.777
121.242
120.990
114.732
112.268
109.802
77.318
77.000
76.683
68.796
66.351
62.984
15.051
13.898
f1 (ppm)

**Ethyl (*E*)-2,2-difluoro-5-(2-hydroxyphenyl)pent-3-enoate (3e).**

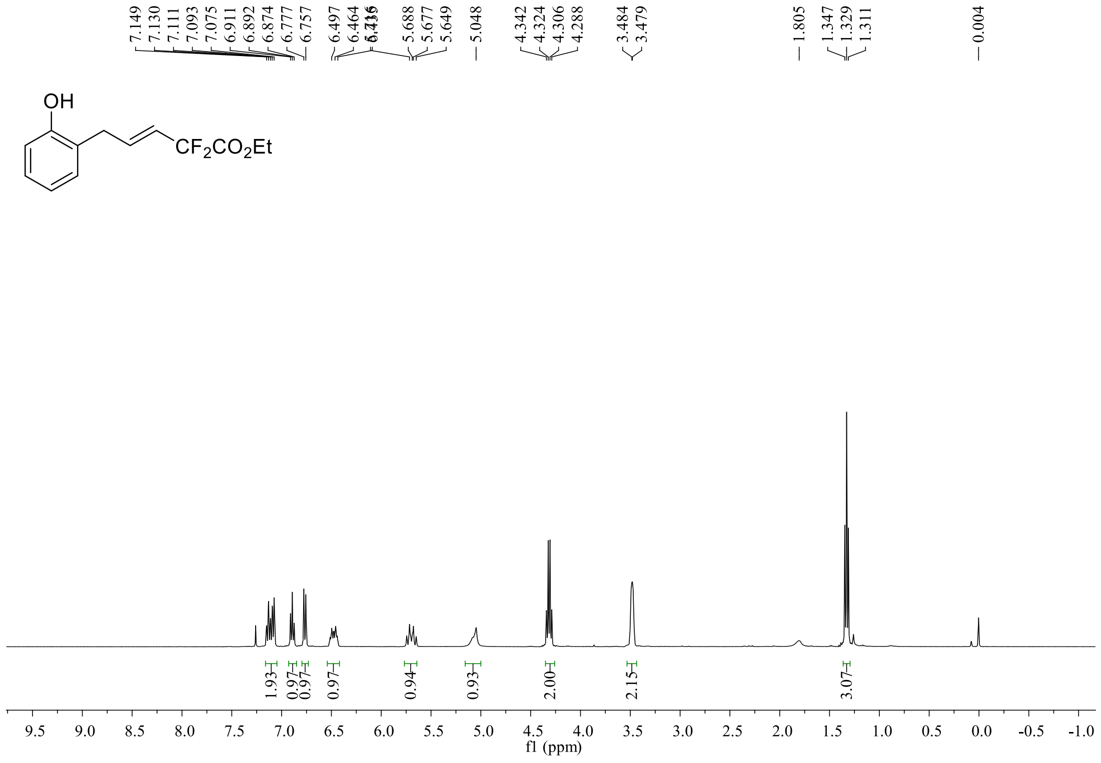


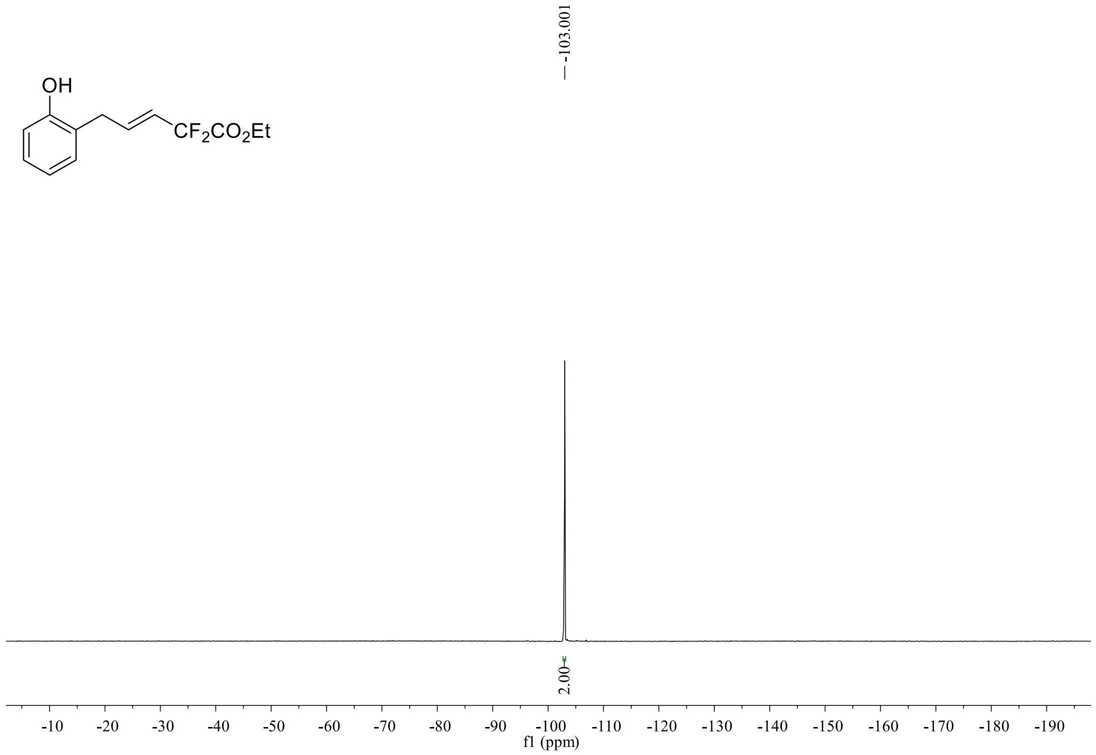

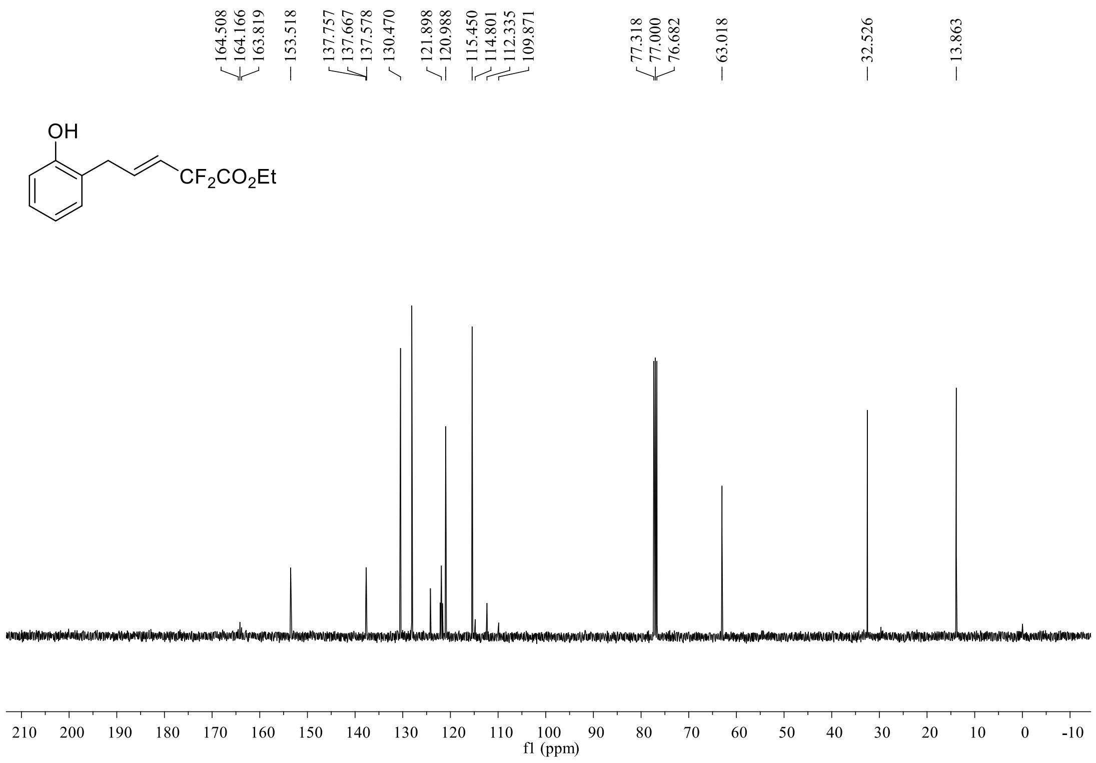


**Ethyl (*E*)-2,2-difluoro-4-phenylbut-3-enoate (3f).**

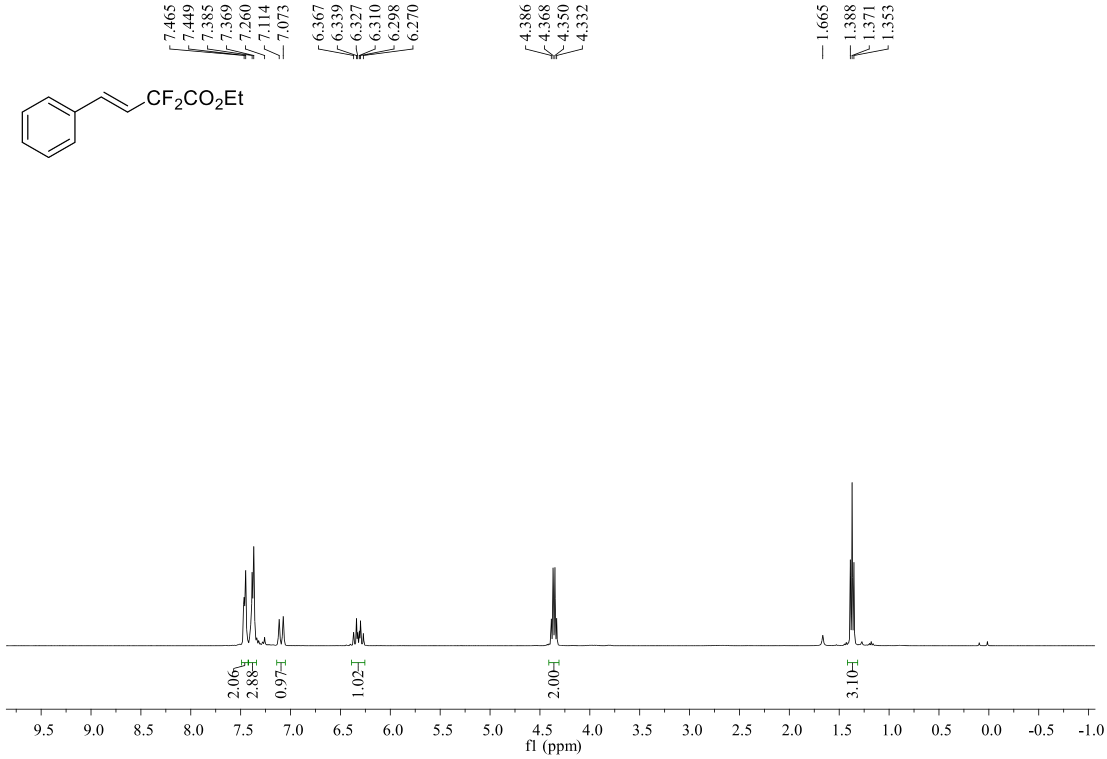

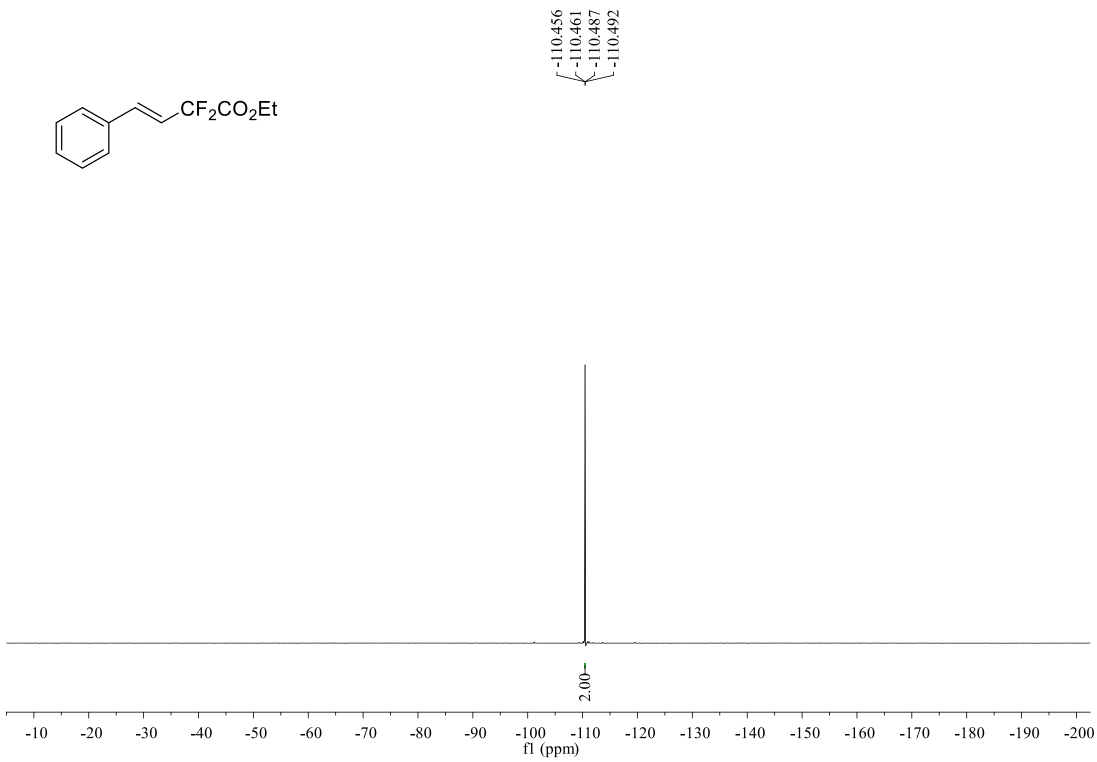
CF2CO2Et
-110.456
-110.461
-110.487
-110.492
2.00
-10 -20 -30 -40 -50 -60 -70 -80 -90 -100 -110 -120 -130 -140 -150 -160 -170 -180 -190 -200
f1 (ppm)

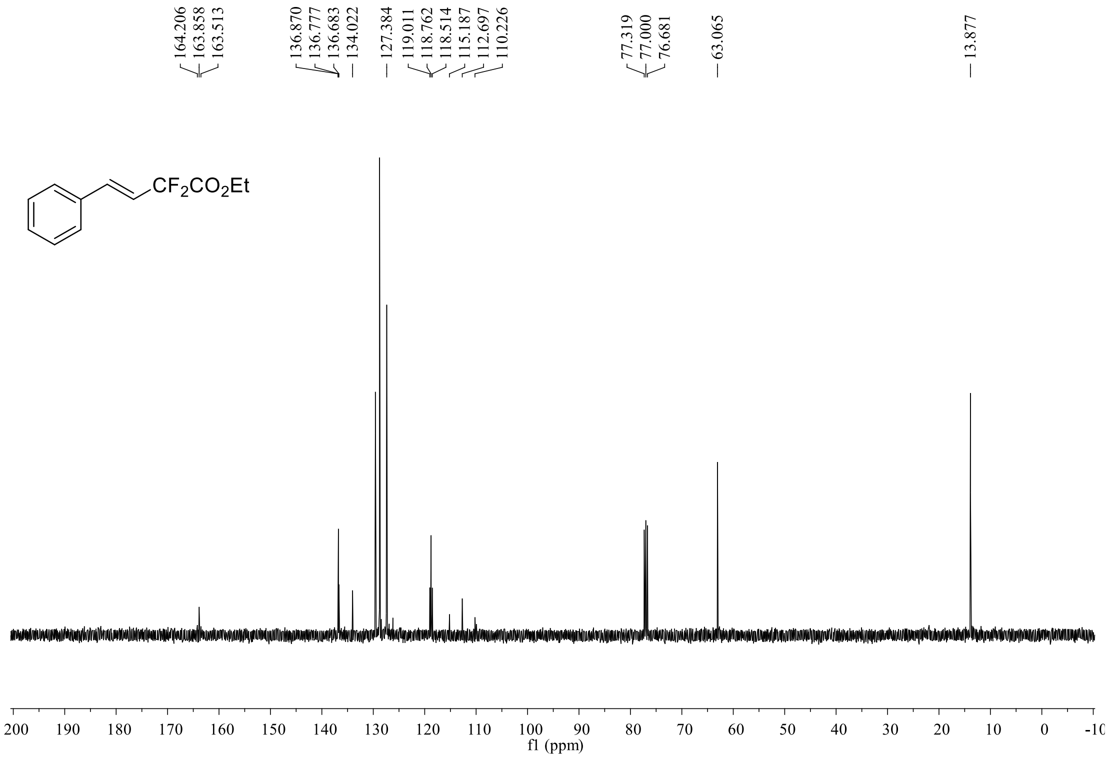
164.206
163.858
163.513
136.870
136.777
136.683
134.022
127.384
119.011
118.762
118.514
115.187
112.697
110.226
77.319
77.000
76.681
63.065
13.877
CF2CO2Et
200 190 180 170 160 150 140 130 120 110 100 90 80 70 60 50 40 30 20 10 0 -10
f1 (ppm)

**Ethyl (*E*)-4-(4-bromophenyl)-2,2-difluorobut-3-enoate (3g).**

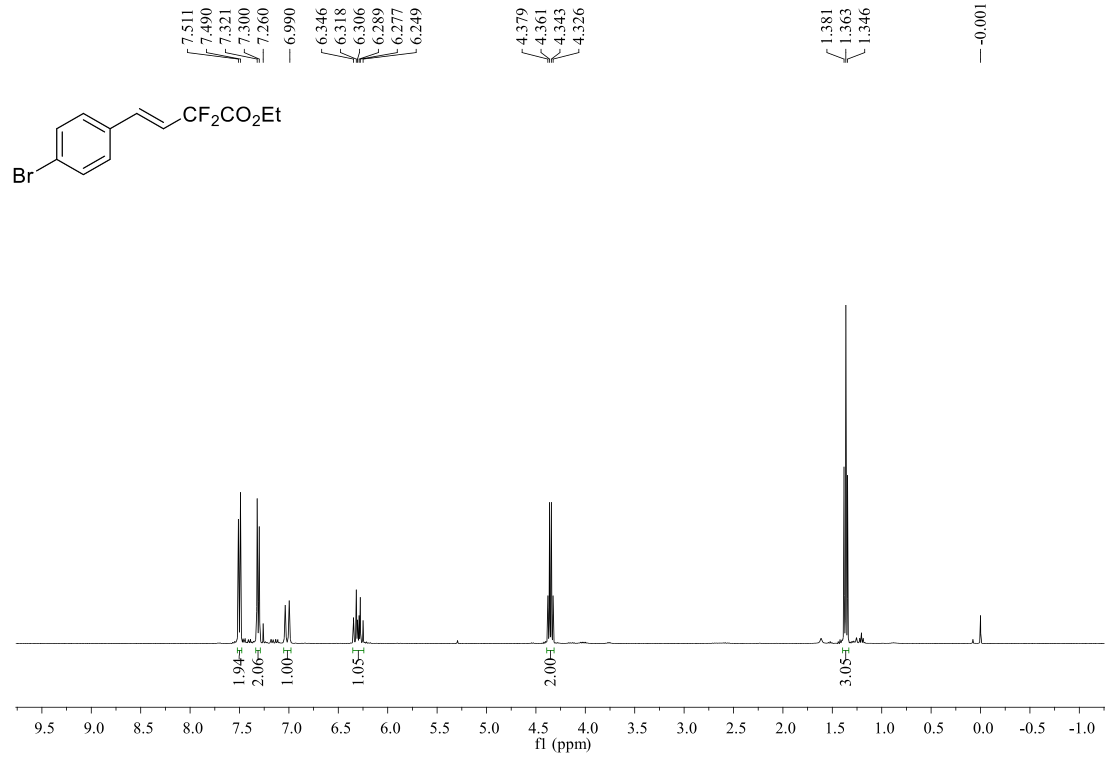


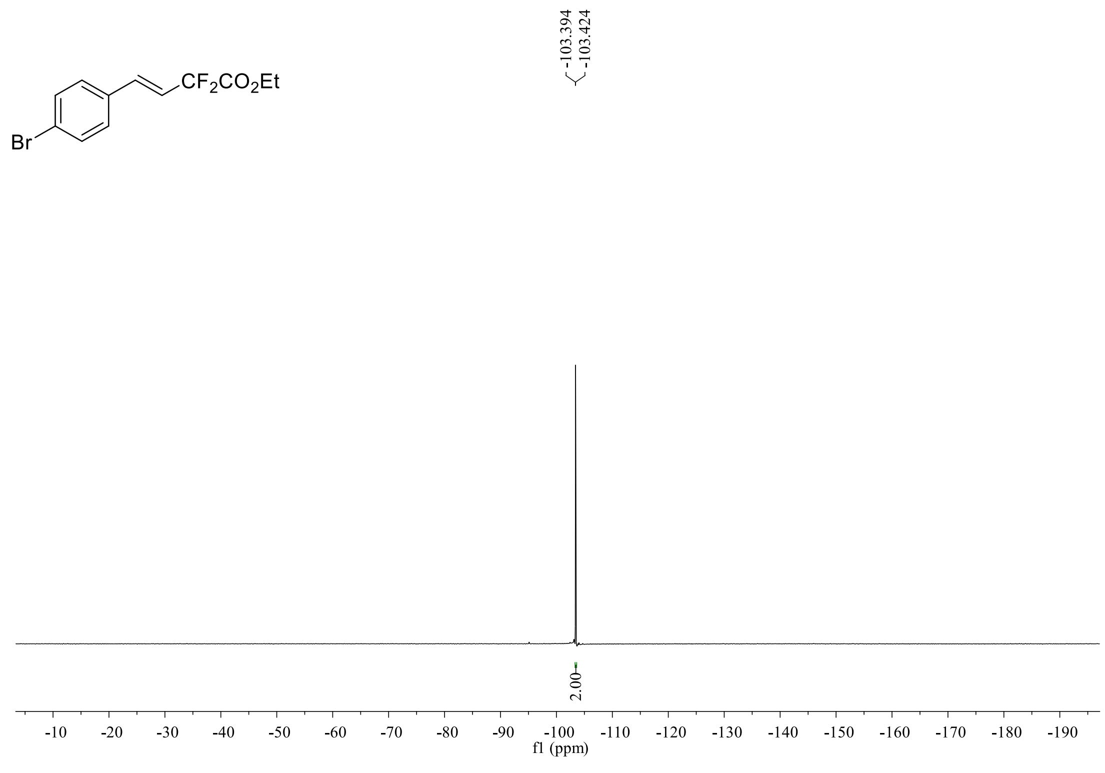

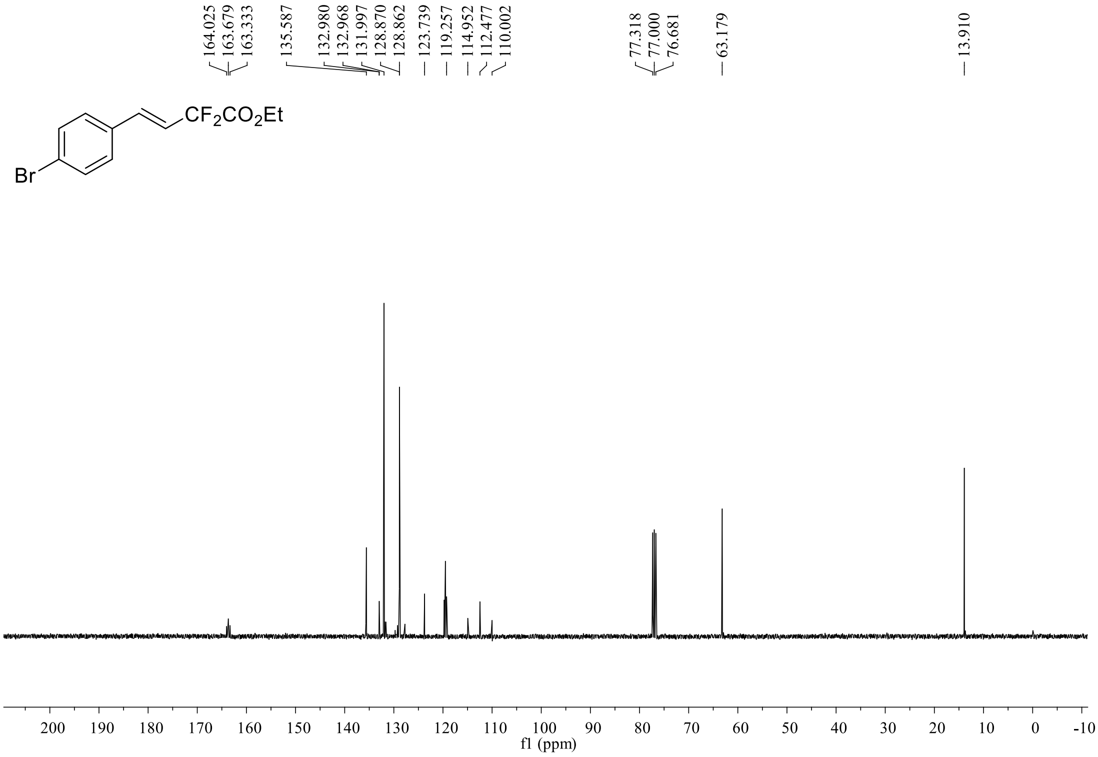


# Ethyl (*E*)-4-(4-chlorophenyl)-2,2-difluorobut-3-enoate (3h).

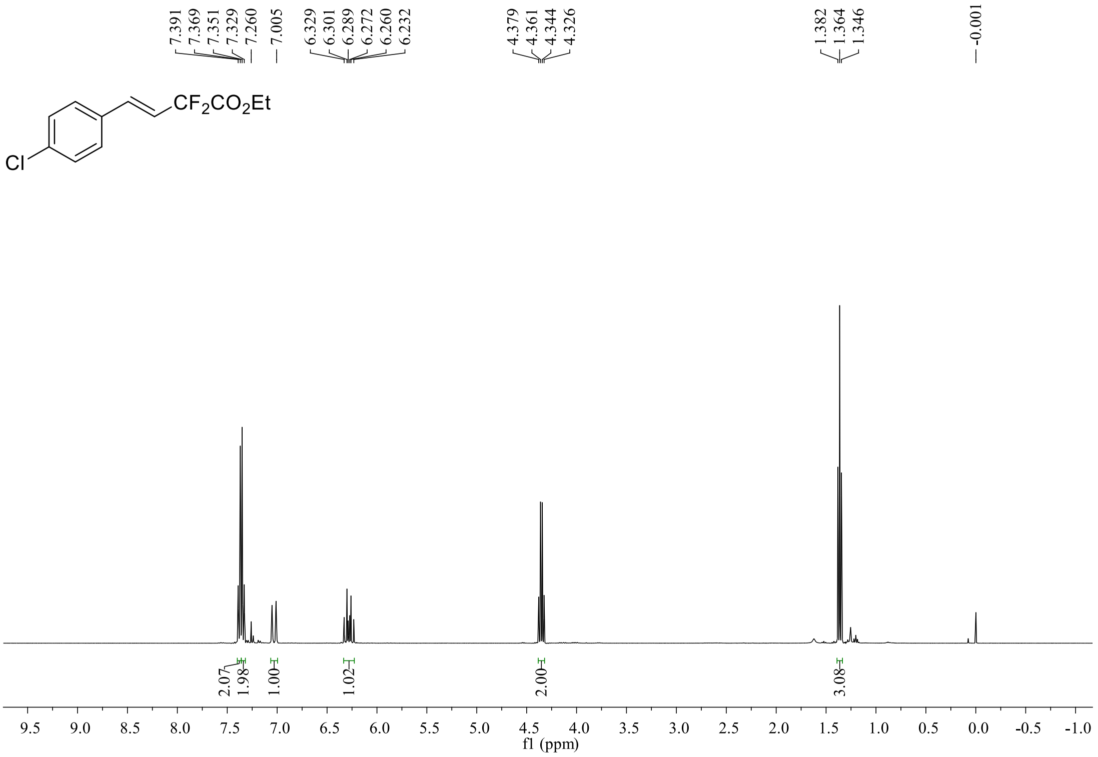

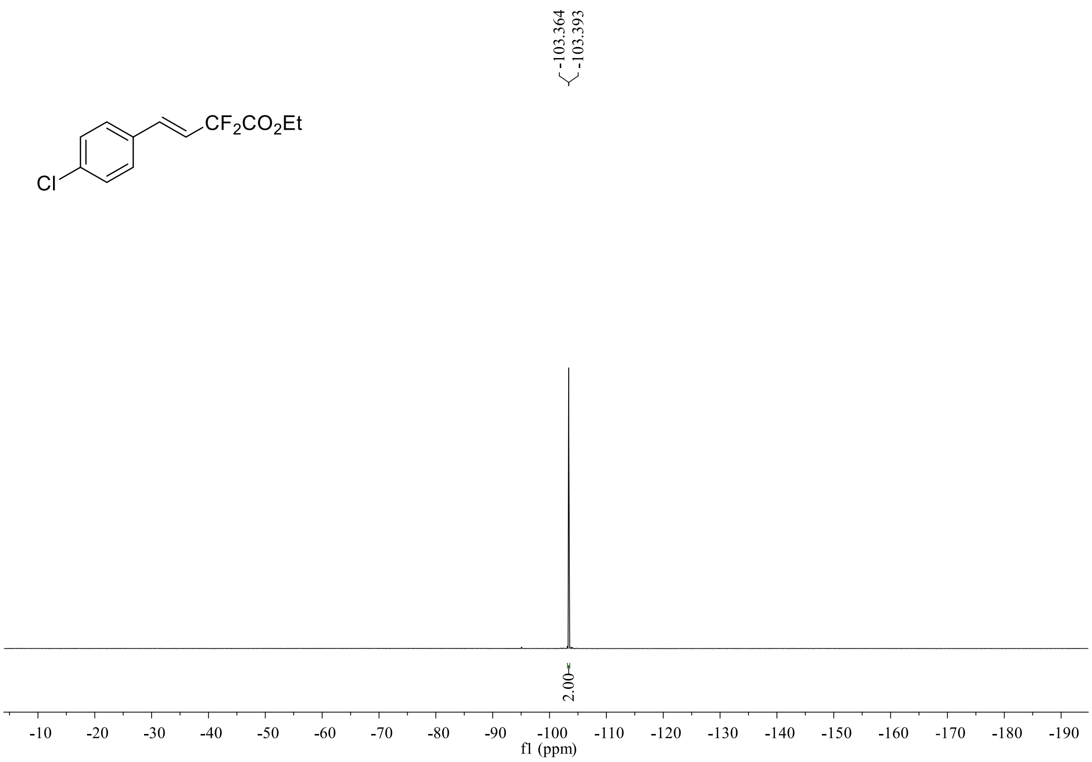
-103.364
-103.393
CF2CO2Et
Cl
2.00
-10 -20 -30 -40 -50 -60 -70 -80 -90 -100 -110 -120 -130 -140 -150 -160 -170 -180 -190
f1 (ppm)

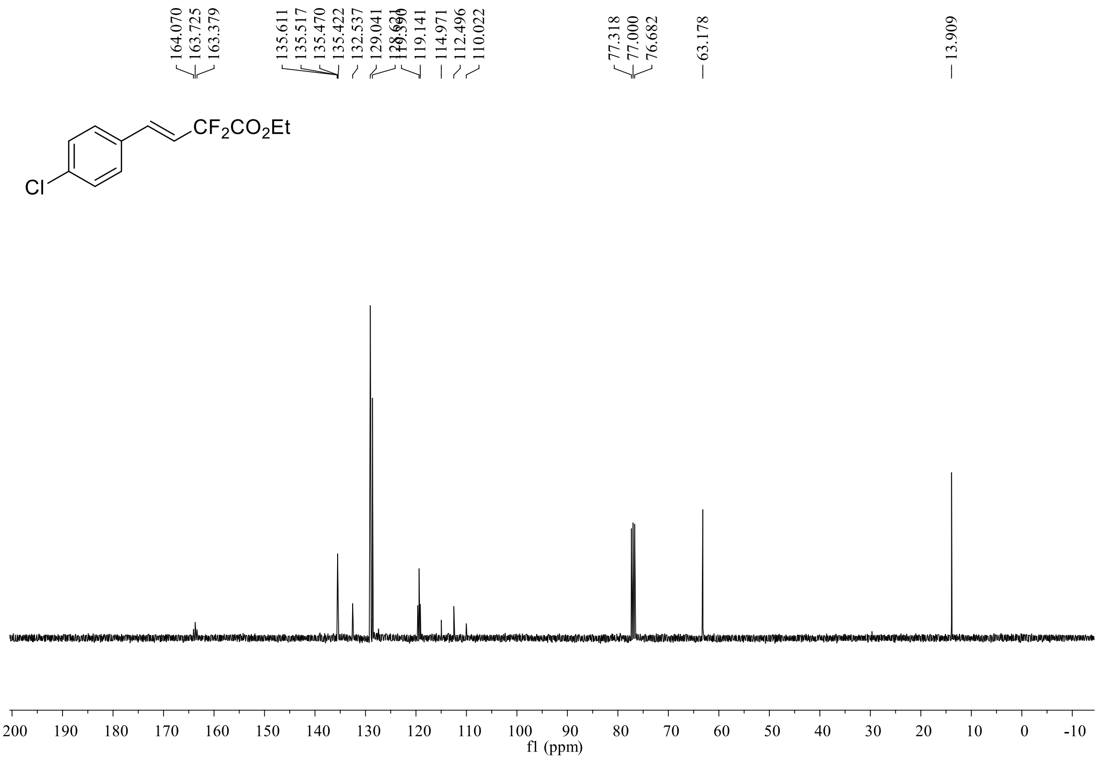
164.070
163.725
163.379
135.611
135.517
135.470
135.422
132.537
129.041
119.141
114.971
112.496
110.022
77.318
77.000
76.682
63.178
13.909
CF2CO2Et
Cl
200 190 180 170 160 150 140 130 120 110 100 90 80 70 60 50 40 30 20 10 0 -10
f1 (ppm)

**Ethyl (*E*)-2,2-difluoro-4-(4-fluorophenyl)but-3-enoate (3i).**

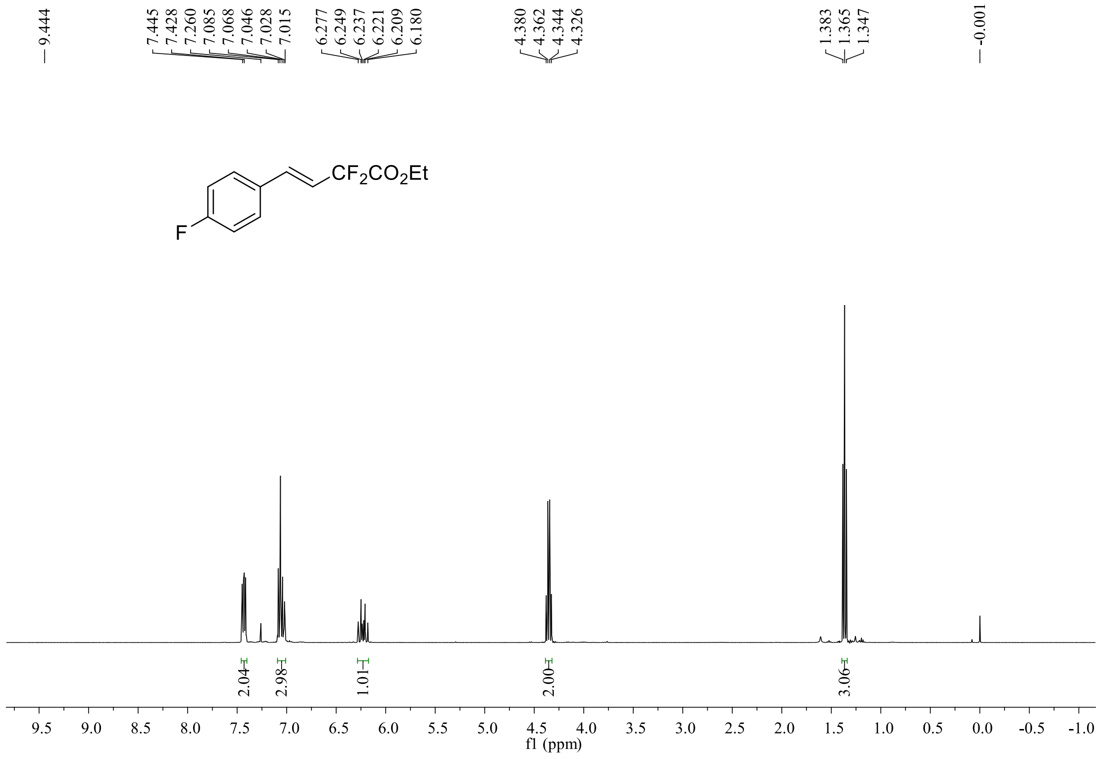


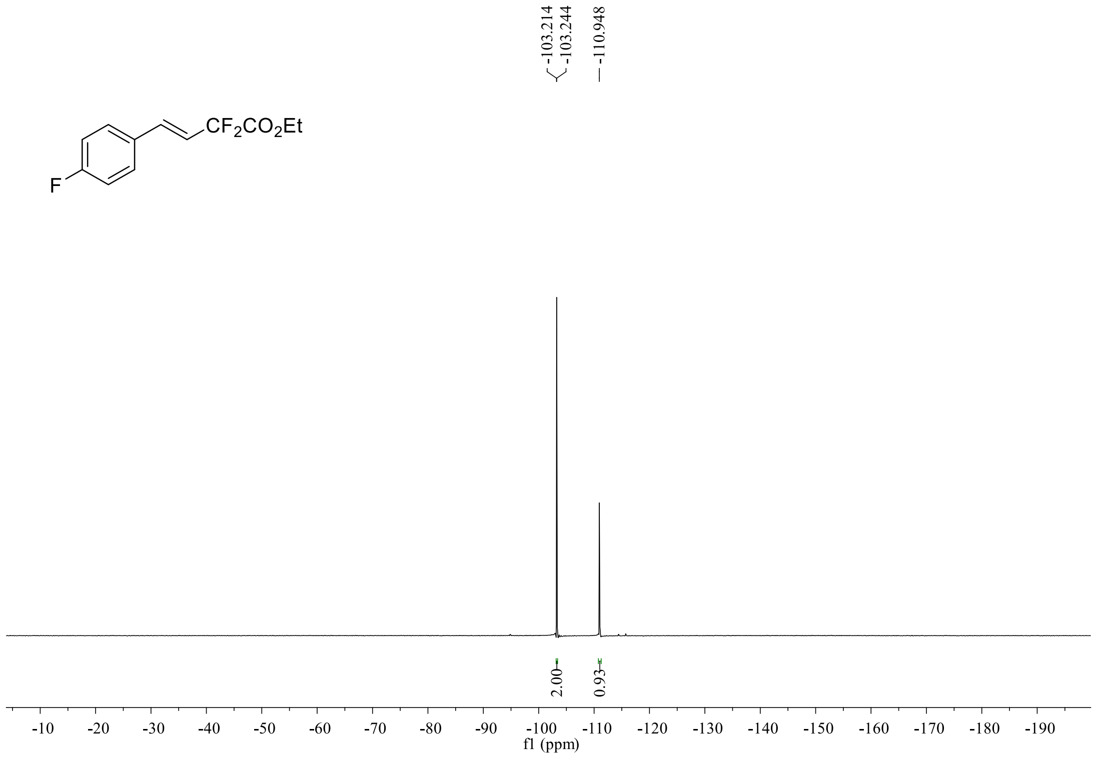

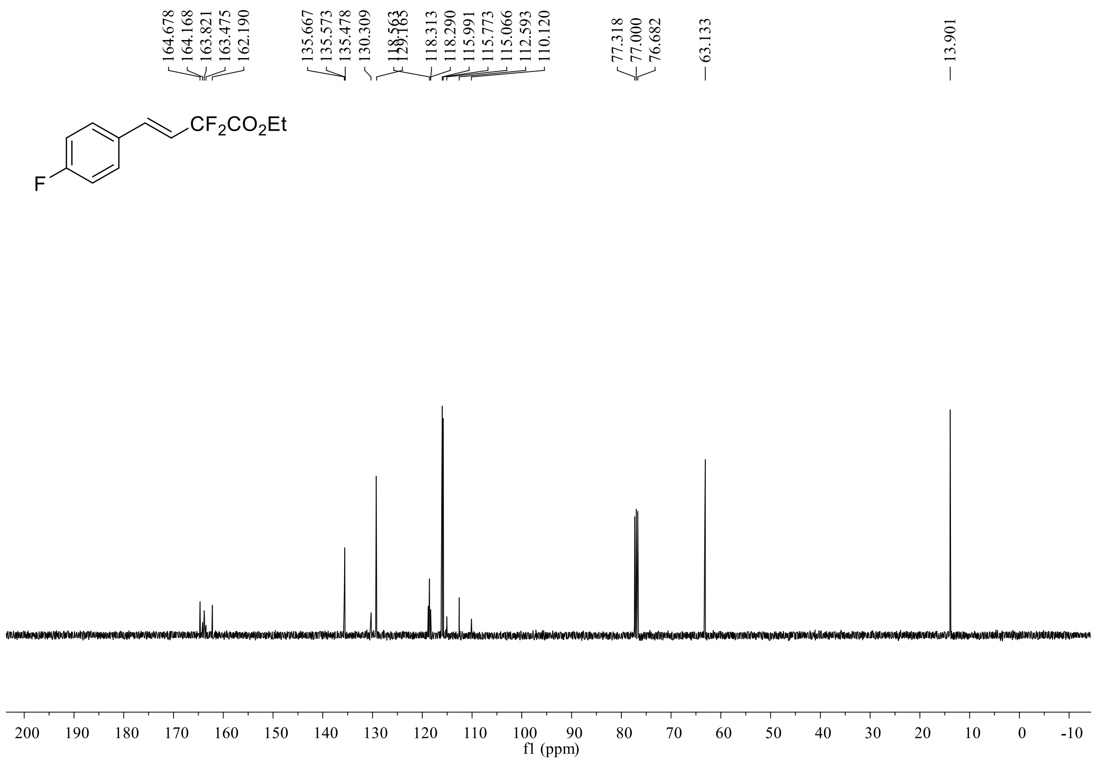


**Ethyl (*E*)-2,2-difluoro-4-(p-tolyl)but-3-enoate (3j).**

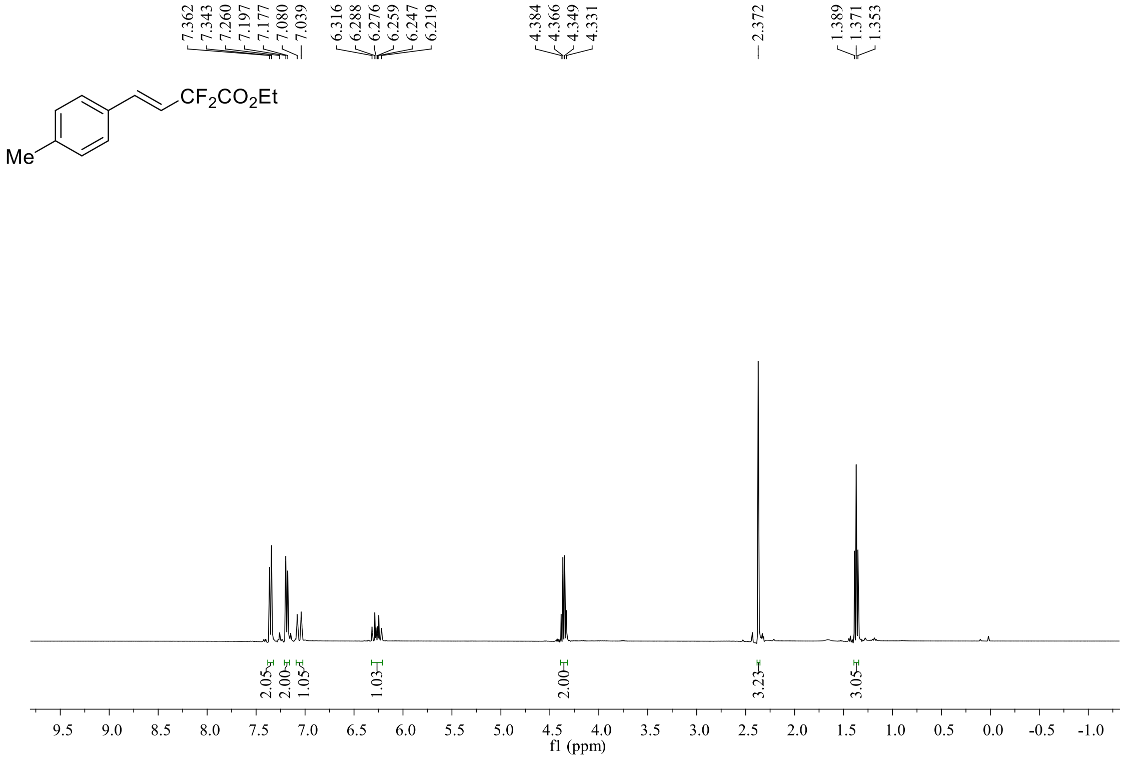

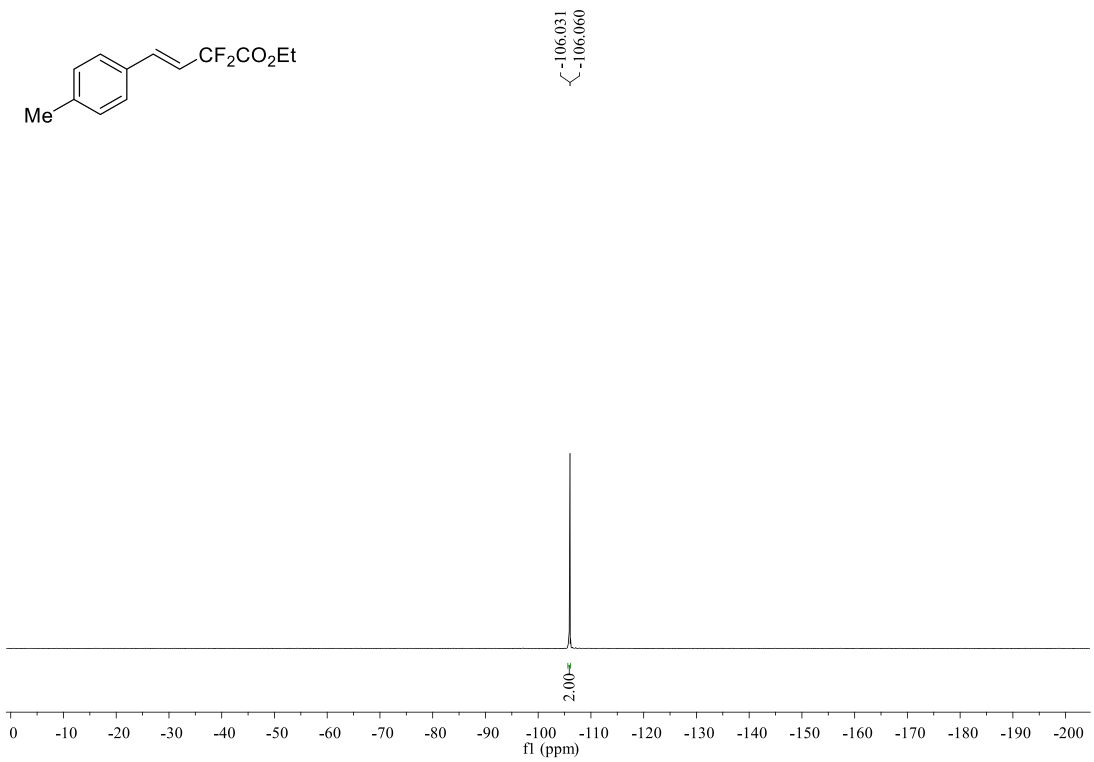
CF2CO2Et
Me
-106.031
-106.060
2.00
f1 (ppm)

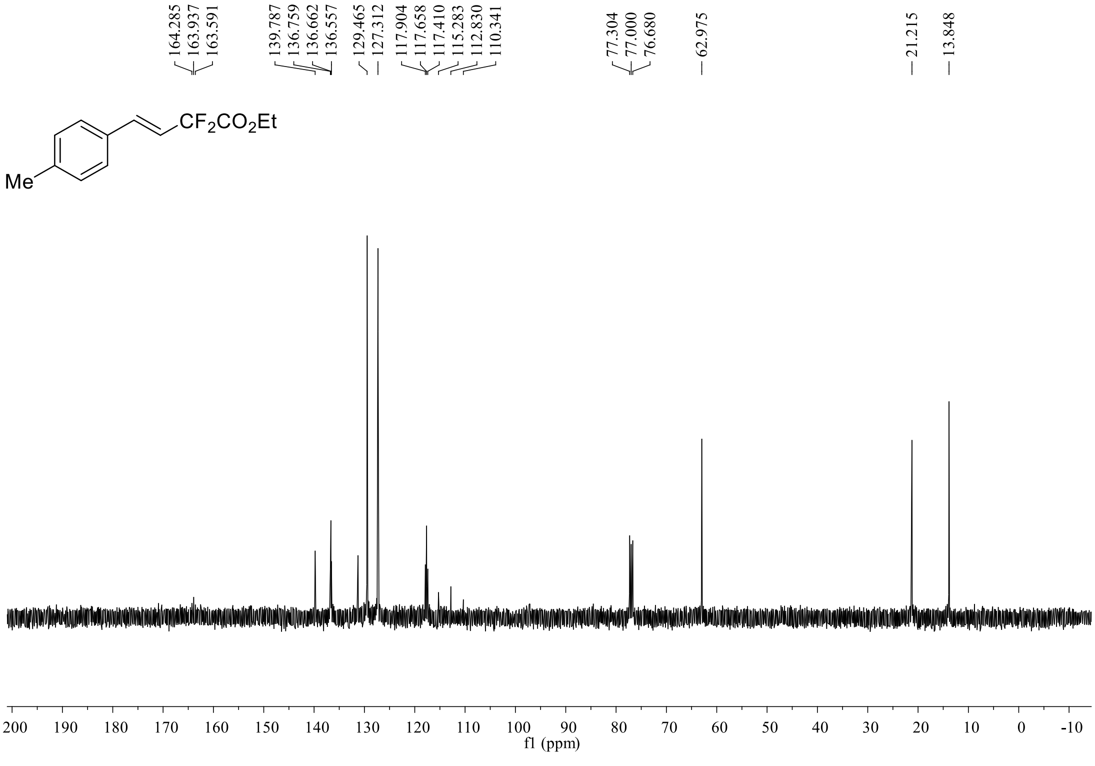
164.285
163.937
163.591
139.787
136.759
136.662
136.557
129.465
127.312
117.904
117.658
117.410
115.283
112.830
110.341
77.304
77.000
76.680
62.975
21.215
13.848
CF2CO2Et
Me
f1 (ppm)

**Ethyl (*E*)-4-(4-(tert-butyl)phenyl)-2,2-difluorobut-3-enoate (3k).**

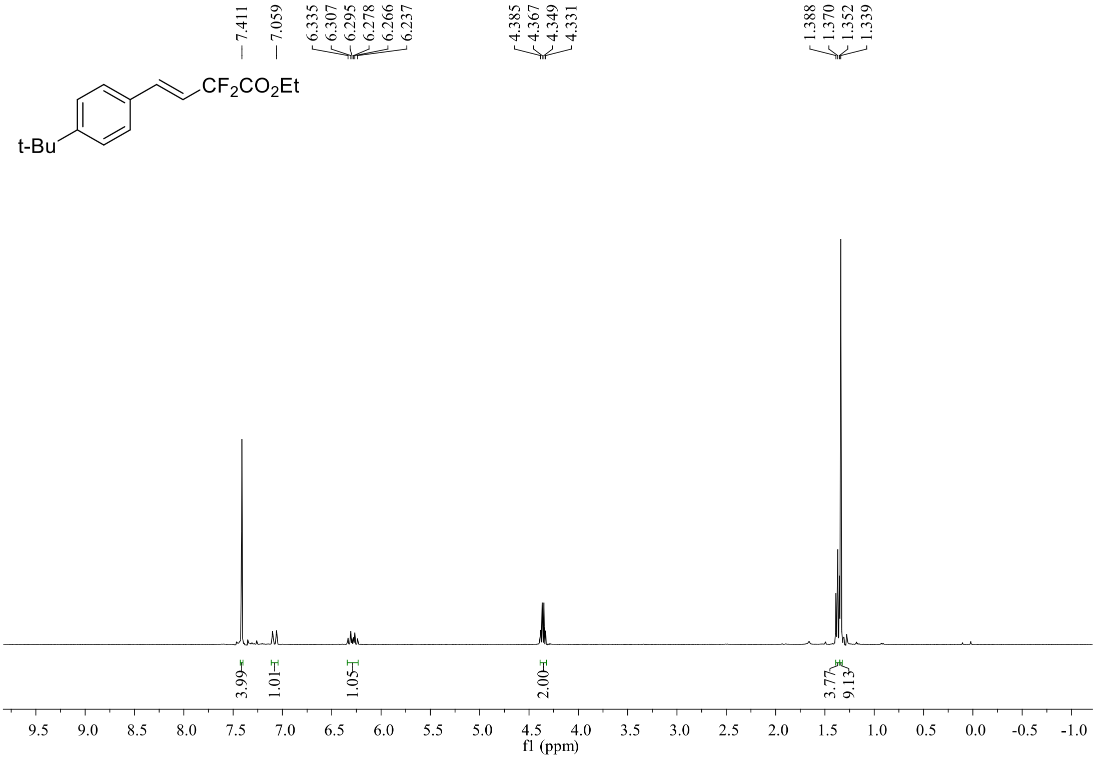


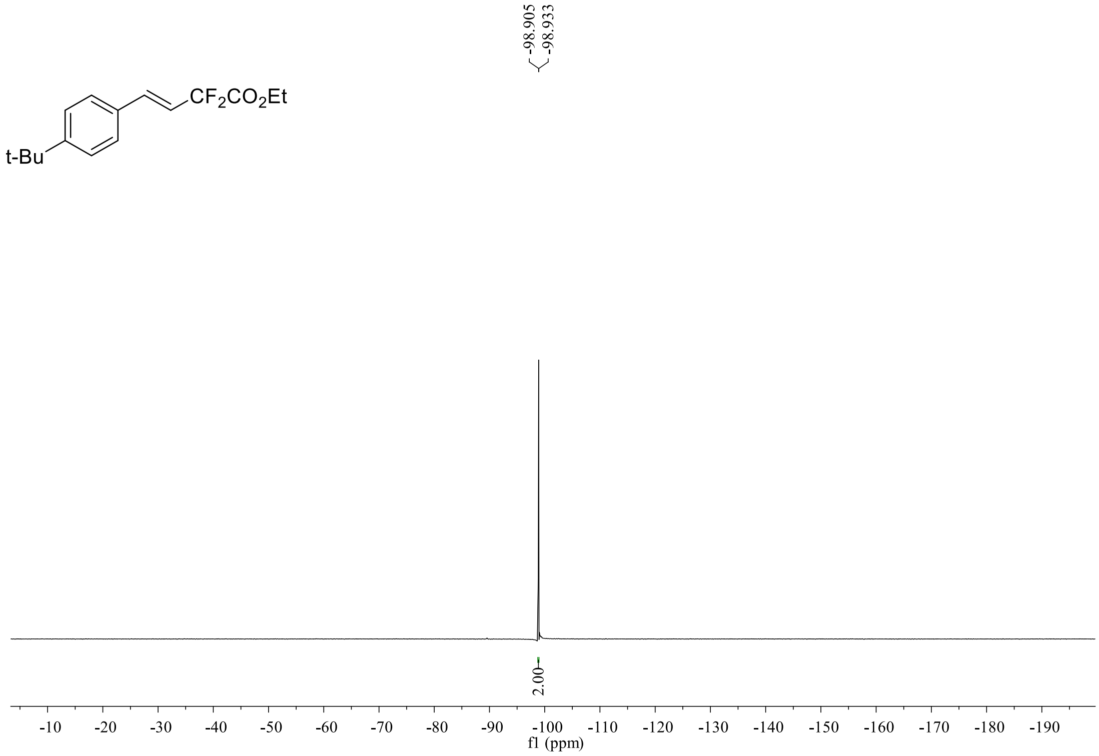

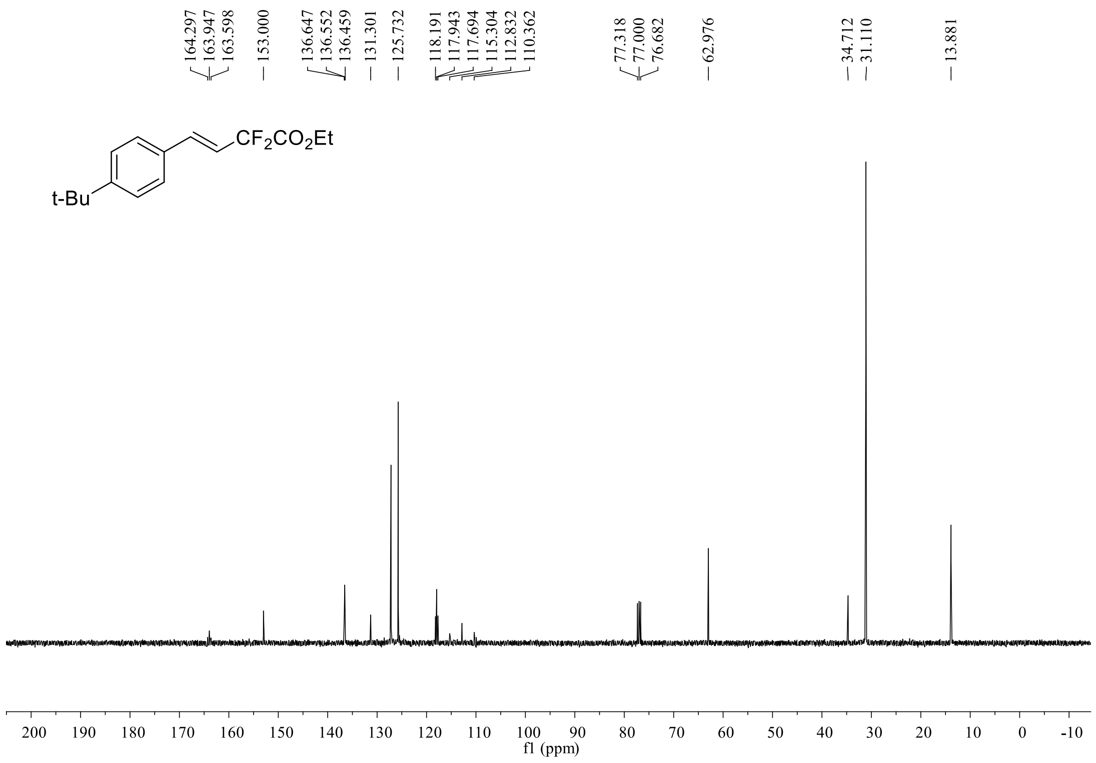


**Ethyl (*E*)-4-(4-acetoxyphenyl)-2,2-difluorobut-3-enoate (3l).**

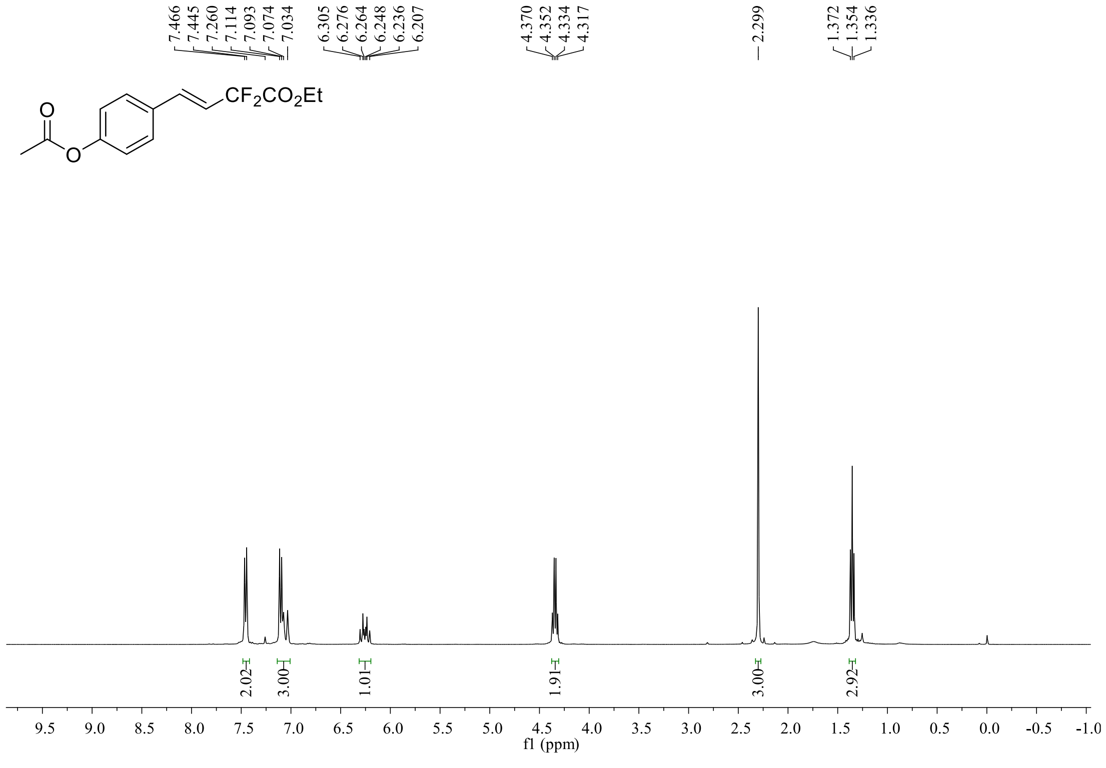

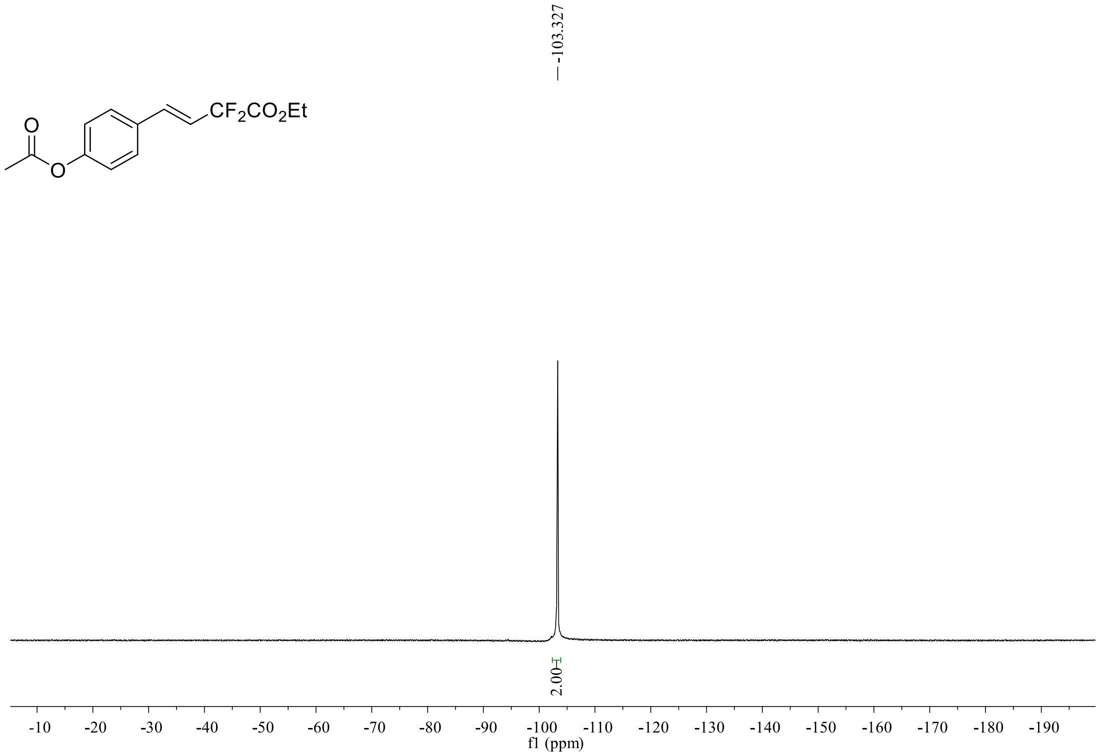
CF2CO2Et
-103.327
2.00
-10 -20 -30 -40 -50 -60 -70 -80 -90 -100 -110 -120 -130 -140 -150 -160 -170 -180 -190
f1 (ppm)

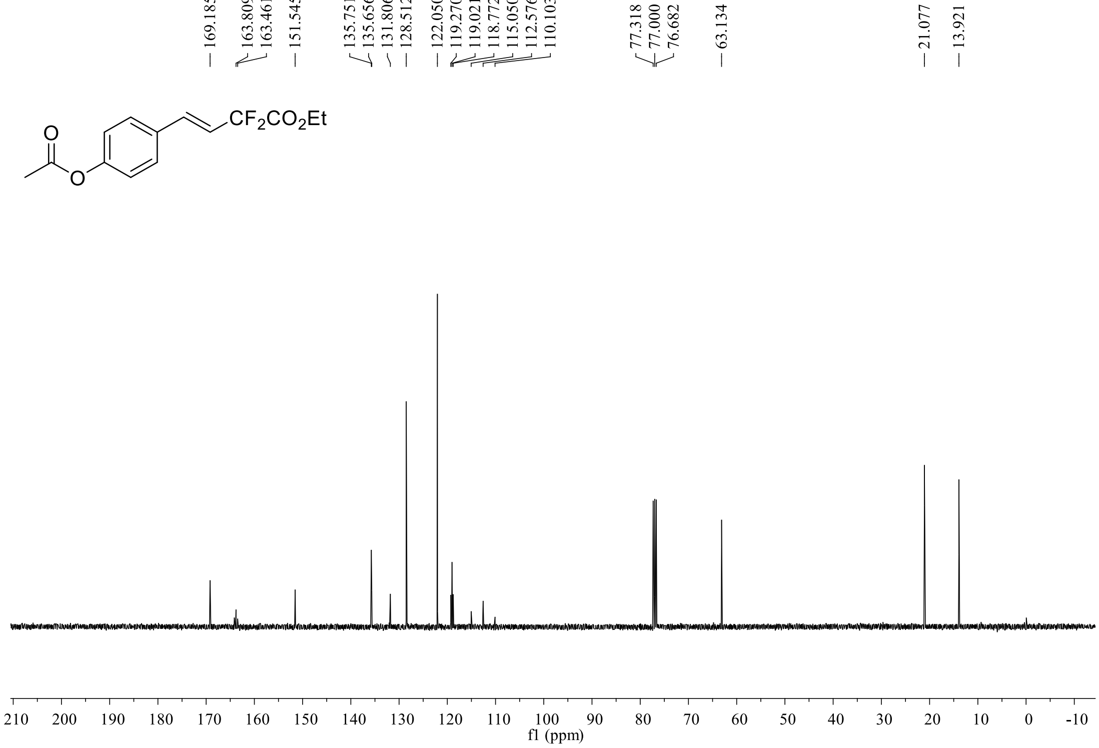
CF2CO2Et
169.185
163.809
163.461
151.545
135.751
135.656
131.806
128.512
122.050
119.270
119.021
118.772
115.050
112.576
110.103
77.318
77.000
76.682
63.134
21.077
13.921
210 200 190 180 170 160 150 140 130 120 110 100 90 80 70 60 50 40 30 20 10 0 -10
f1 (ppm)

**Ethyl (*E*)-2,2-difluoro-4-(naphthalen-2-yl)but-3-enoate (3m).**

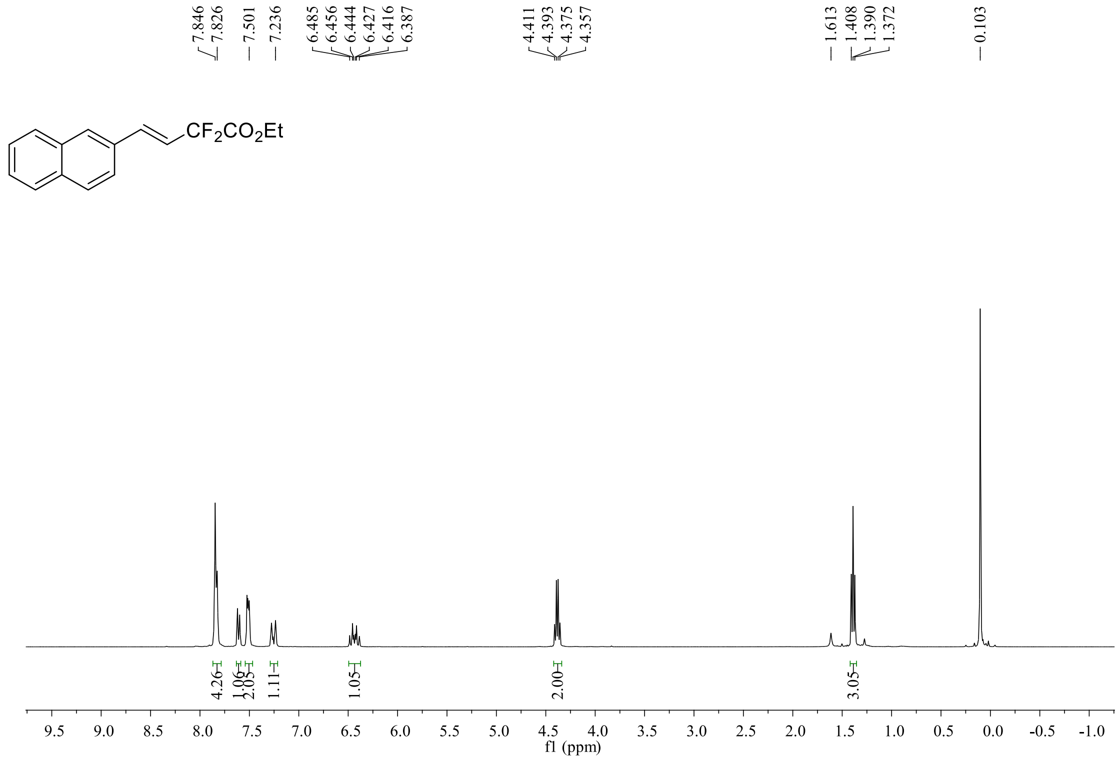


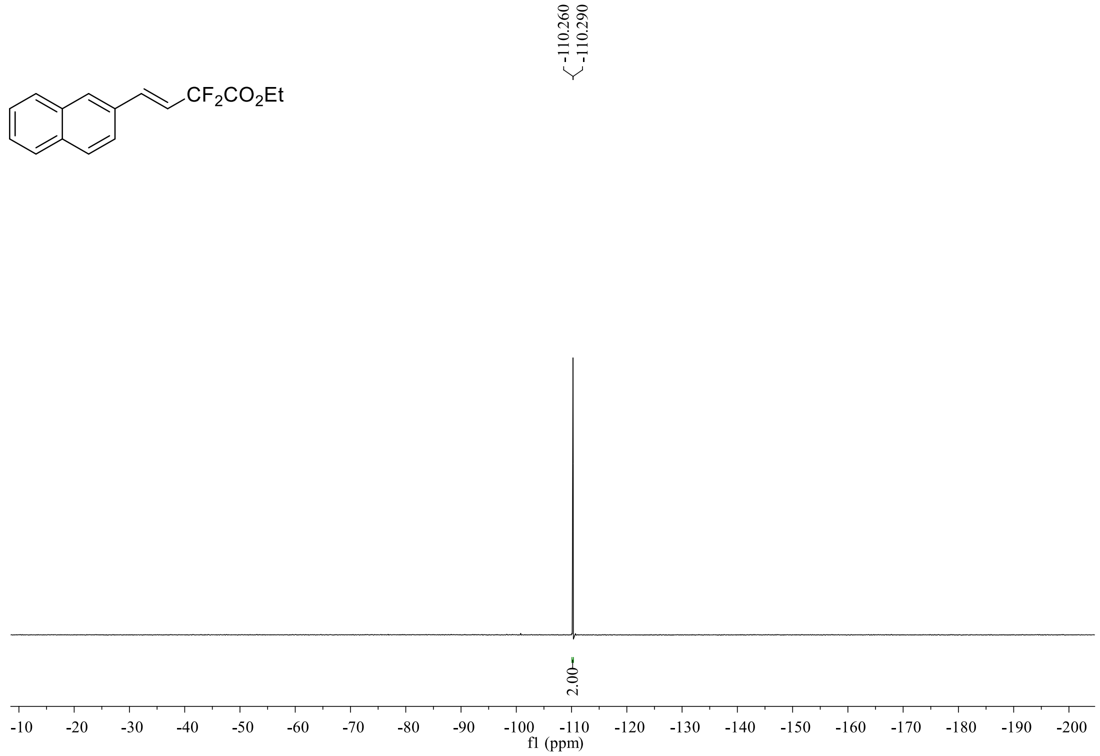

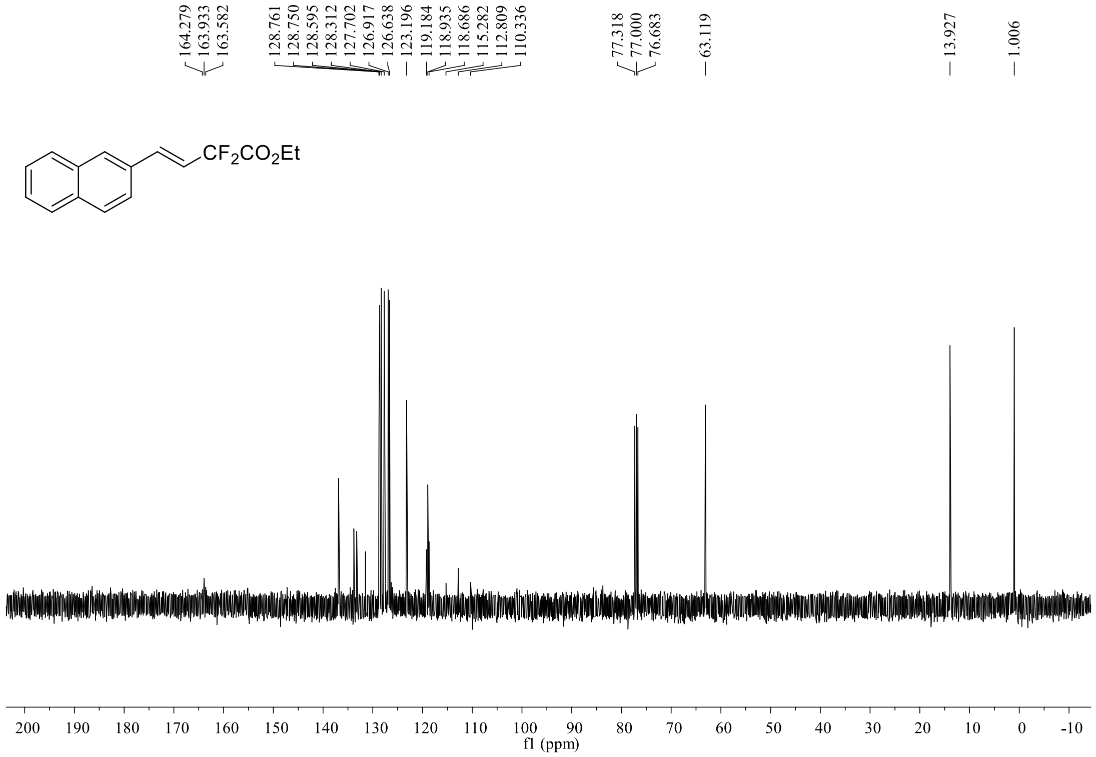


**Ethyl 2,2-difluoro-4,4-diphenylbut-3-enoate (3n).**

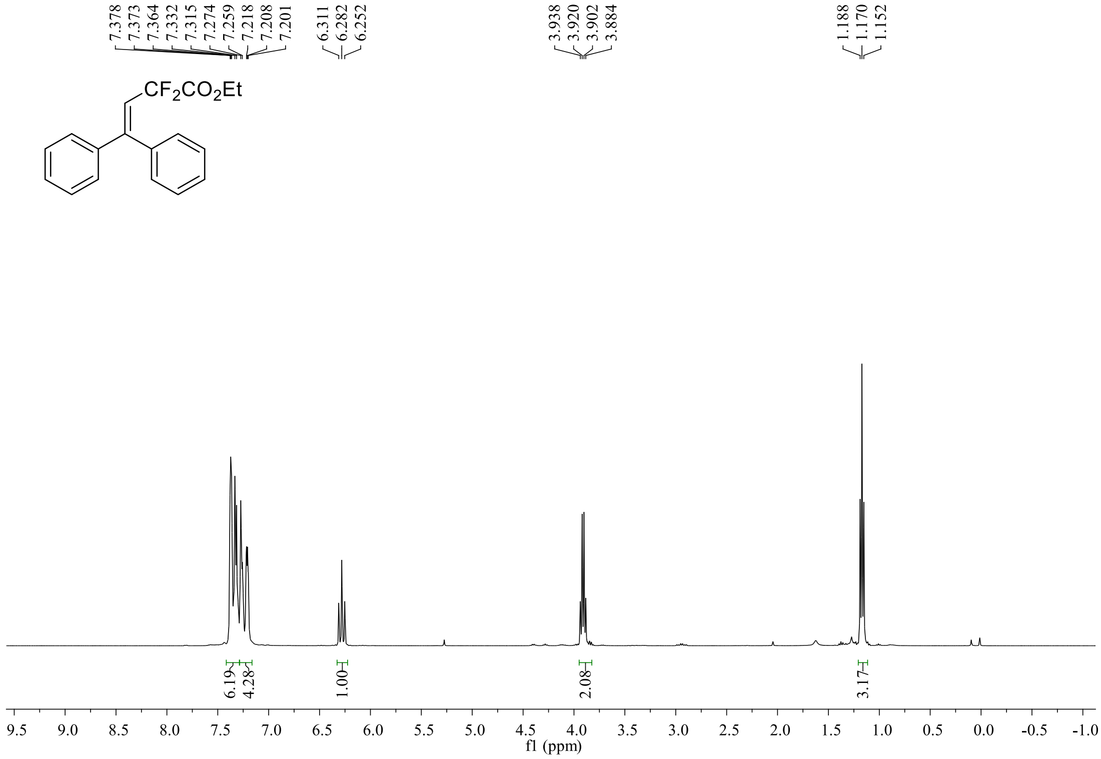

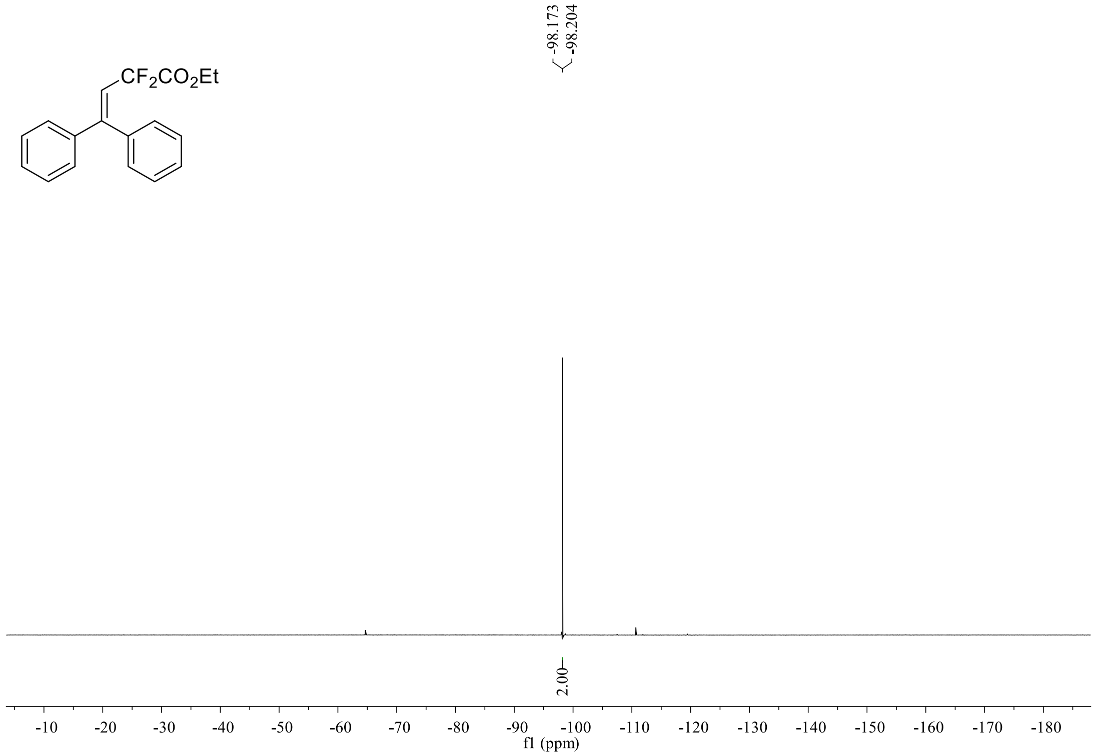
CF2CO2Et
-98.173
-98.204
2.00
f1 (ppm)

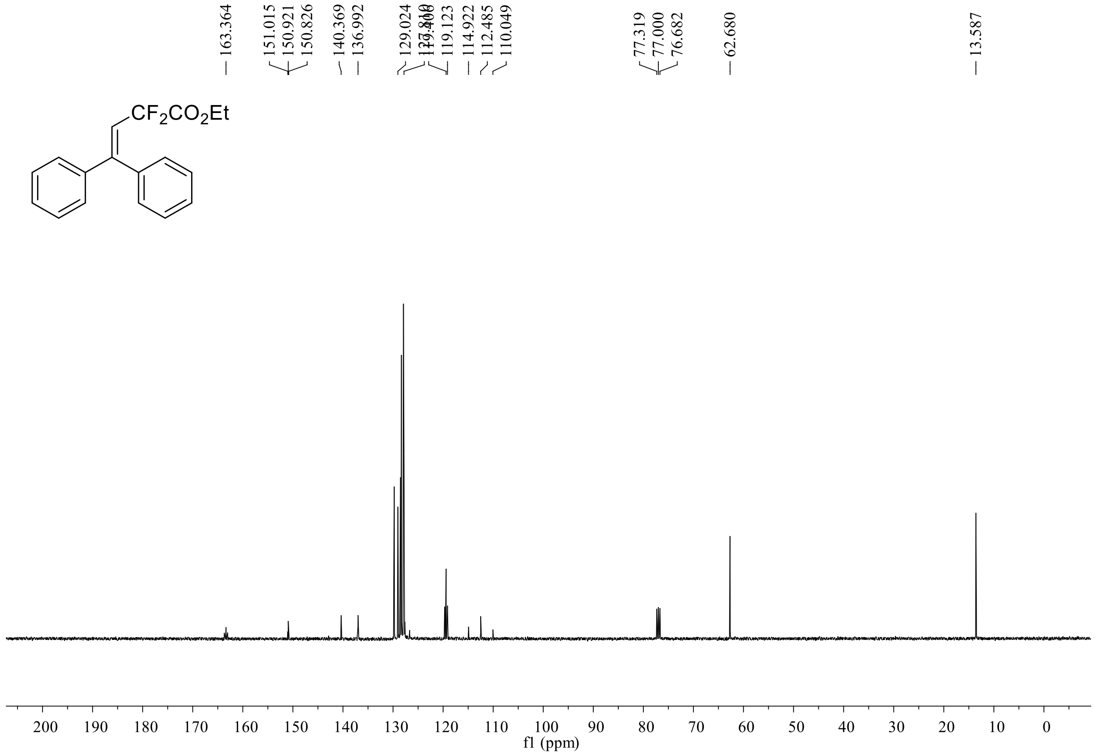
CF2CO2Et
163.364
151.015
150.921
150.826
140.369
136.992
129.024
119.123
114.922
112.485
110.049
77.319
77.000
76.682
62.680
13.587
f1 (ppm)

**Ethyl (*E*)-2,2-difluoro-3-methyl-4-phenylbut-3-enoate (3o).**

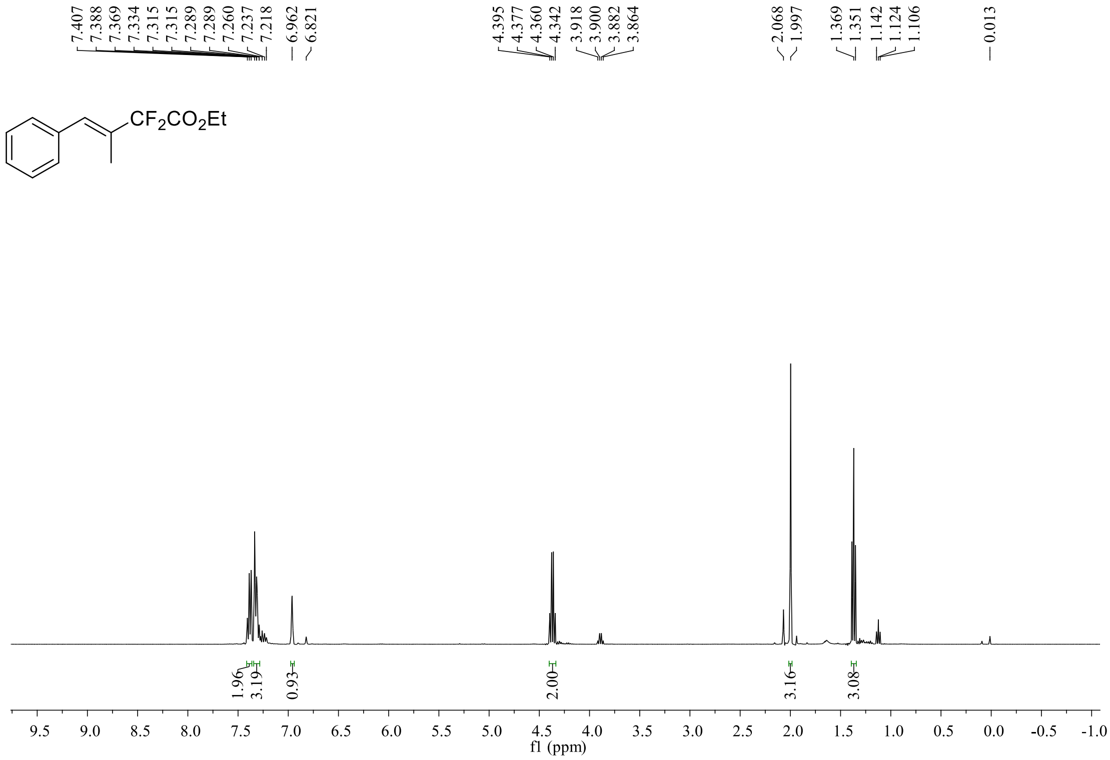

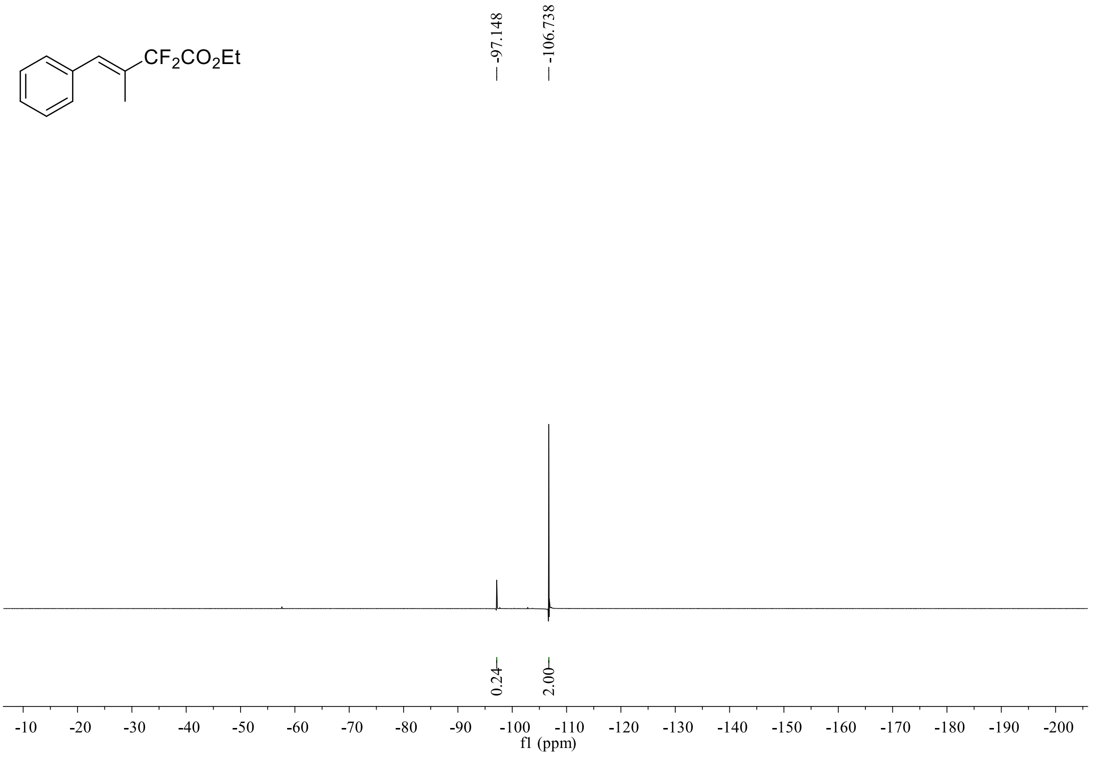

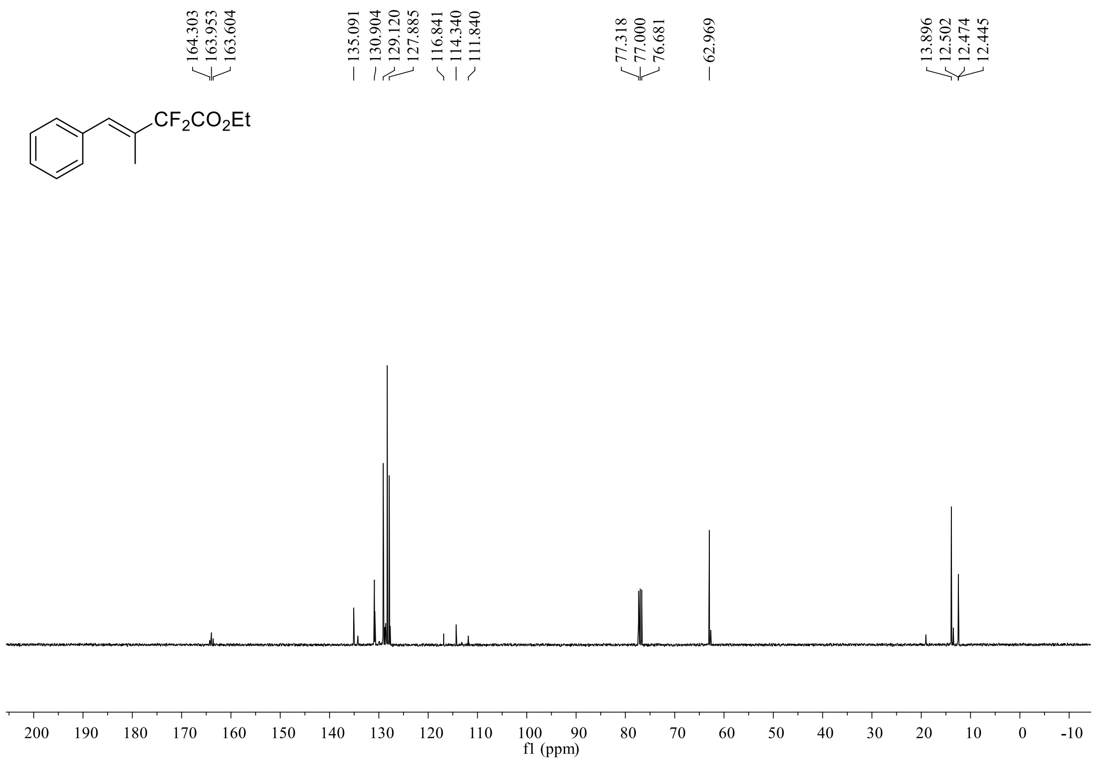


**Tert-butyl (*E*)-(4,4,5,5,6,6,7,7,7-nonafluorohept-2-en-1-yl)carbamate (3p).**

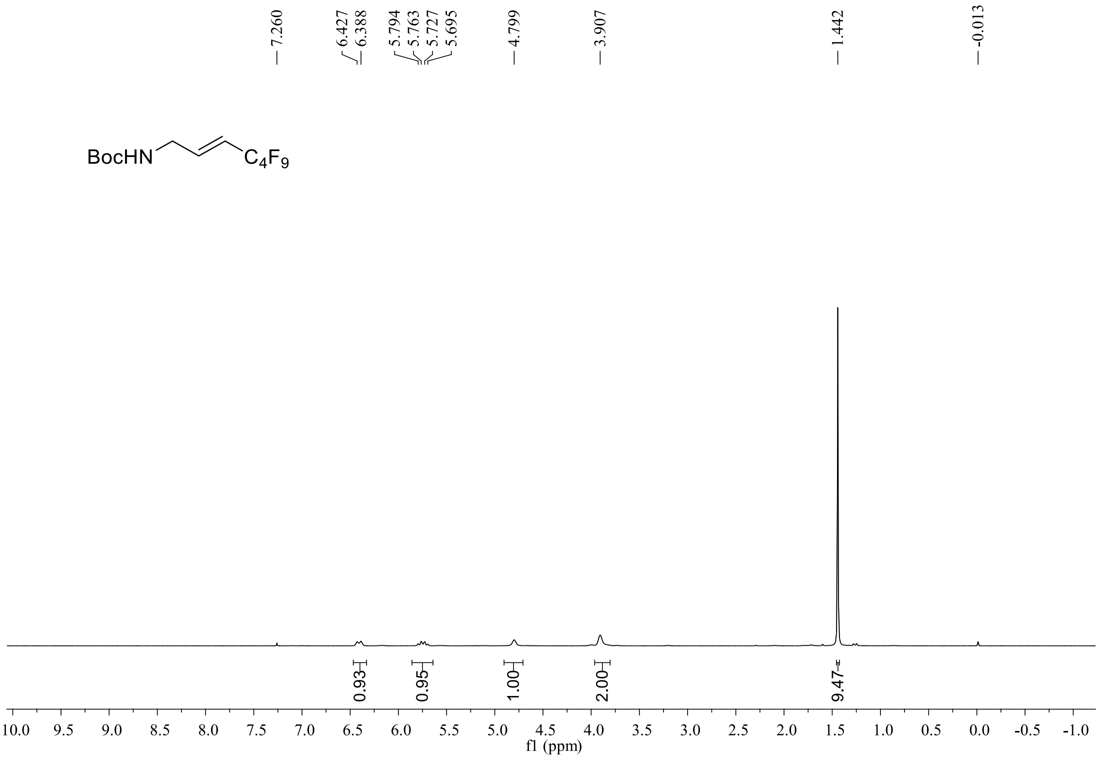

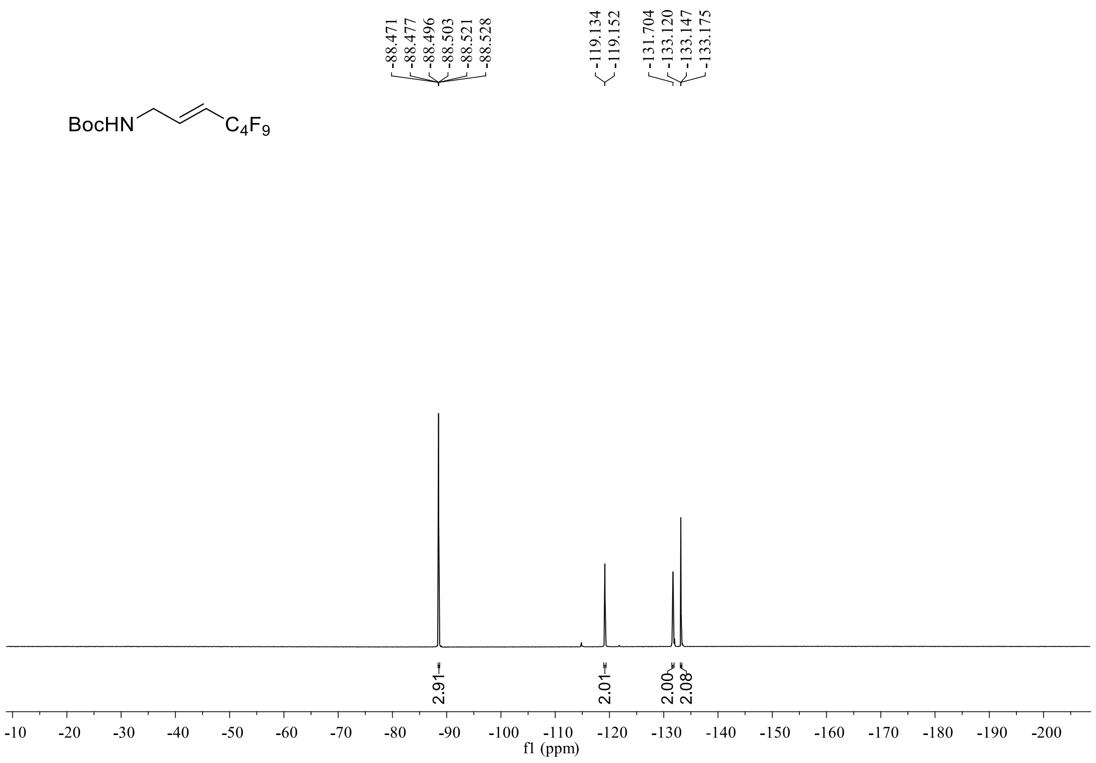
-88.471
-88.477
-88.496
-88.503
-88.521
-88.528
-119.134
-119.152
-131.704
-133.120
-133.147
-133.175
BocHN C4F9
2.91
2.01
2.00
2.08
-10 -20 -30 -40 -50 -60 -70 -80 -90 -100 -110 -120 -130 -140 -150 -160 -170 -180 -190 -200
f1 (ppm)

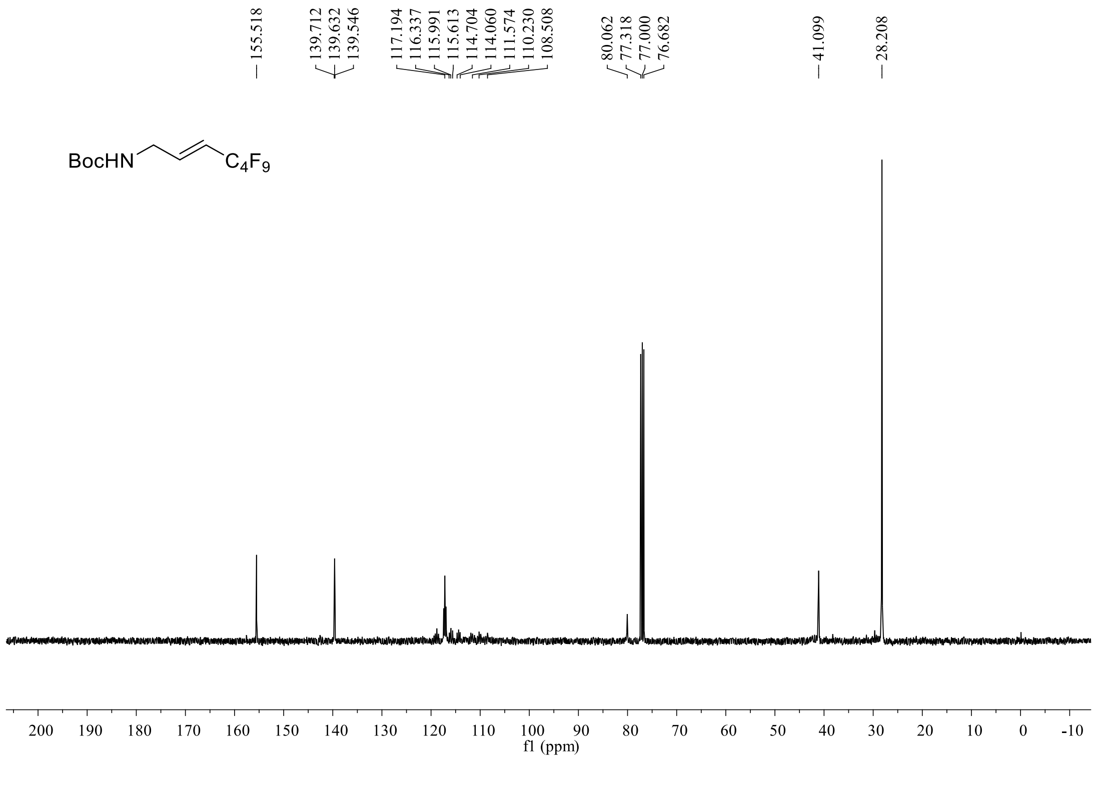
155.518
139.712
139.632
139.546
117.194
116.337
115.991
115.613
114.704
114.060
111.574
110.230
108.508
80.062
77.318
77.000
76.682
41.099
28.208
BocHN C4F9
200 190 180 170 160 150 140 130 120 110 100 90 80 70 60 50 40 30 20 10 0 -10
f1 (ppm)

## (*E*)-(3,3,4,4,5,5,6,6,6-nonafluorohex-1-en-1-yl)benzene (3q).

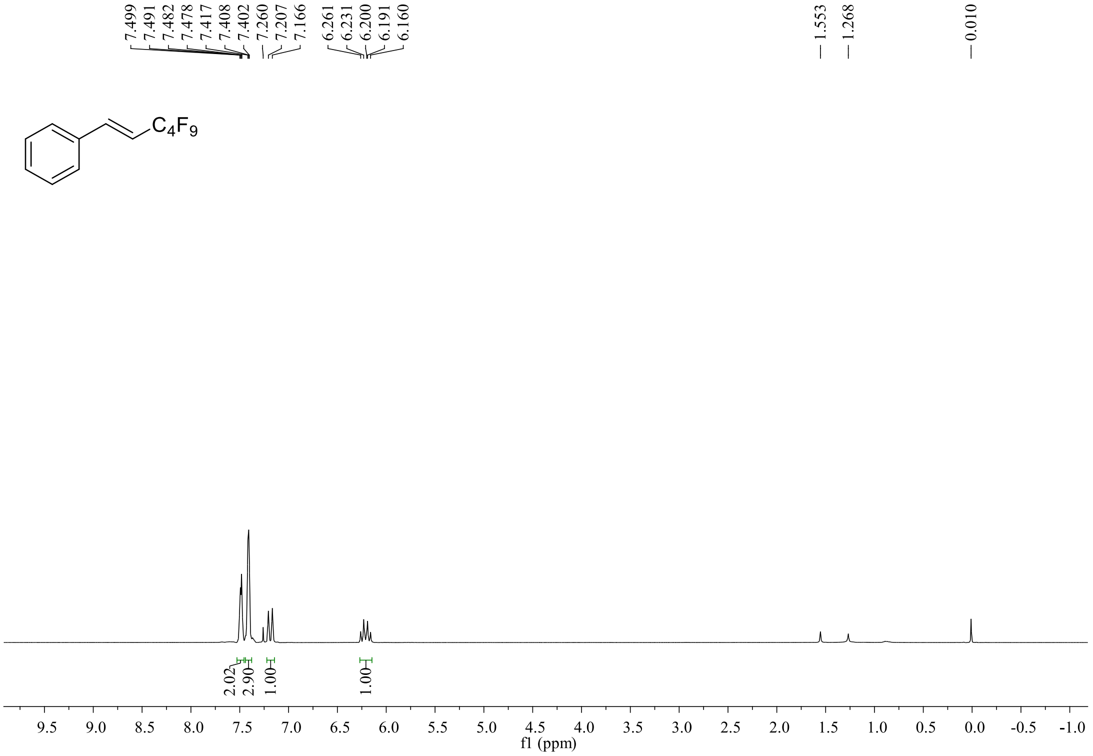


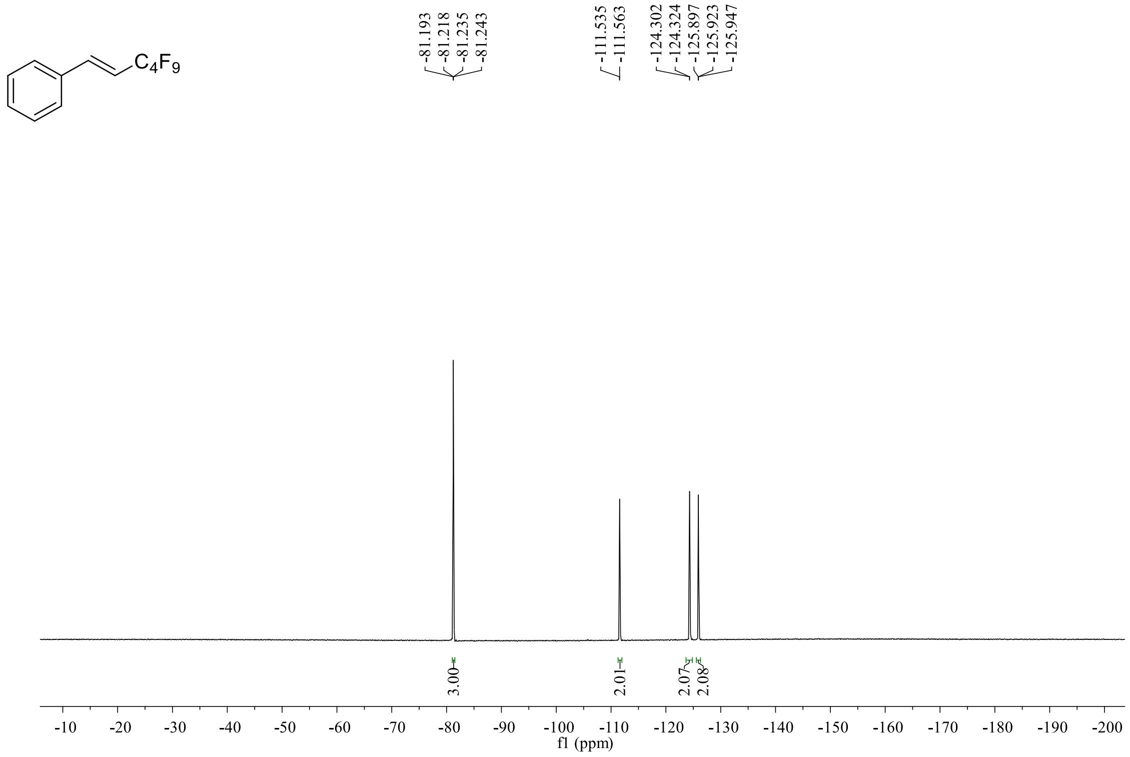

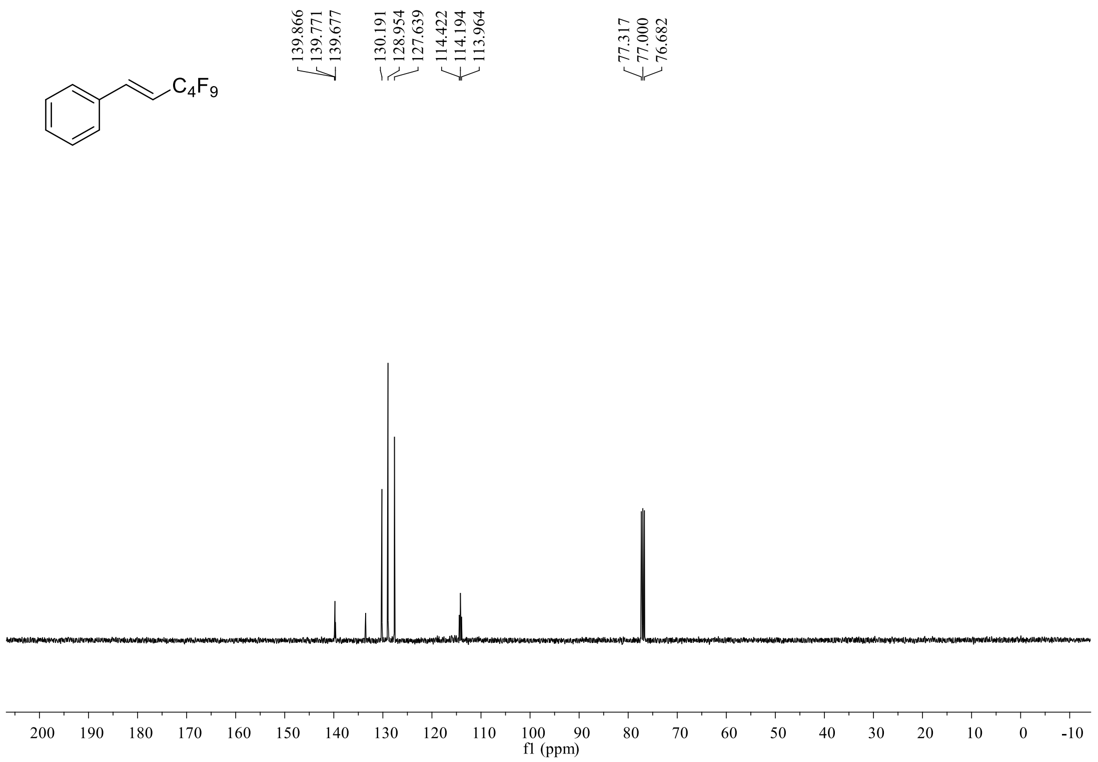


**Tert-butyl(*E*)-(4,4,5,5,6,6,7,7,8,8,9,9,9-tridecafluoronon-2-en-1-yl)carbamate (3r).**

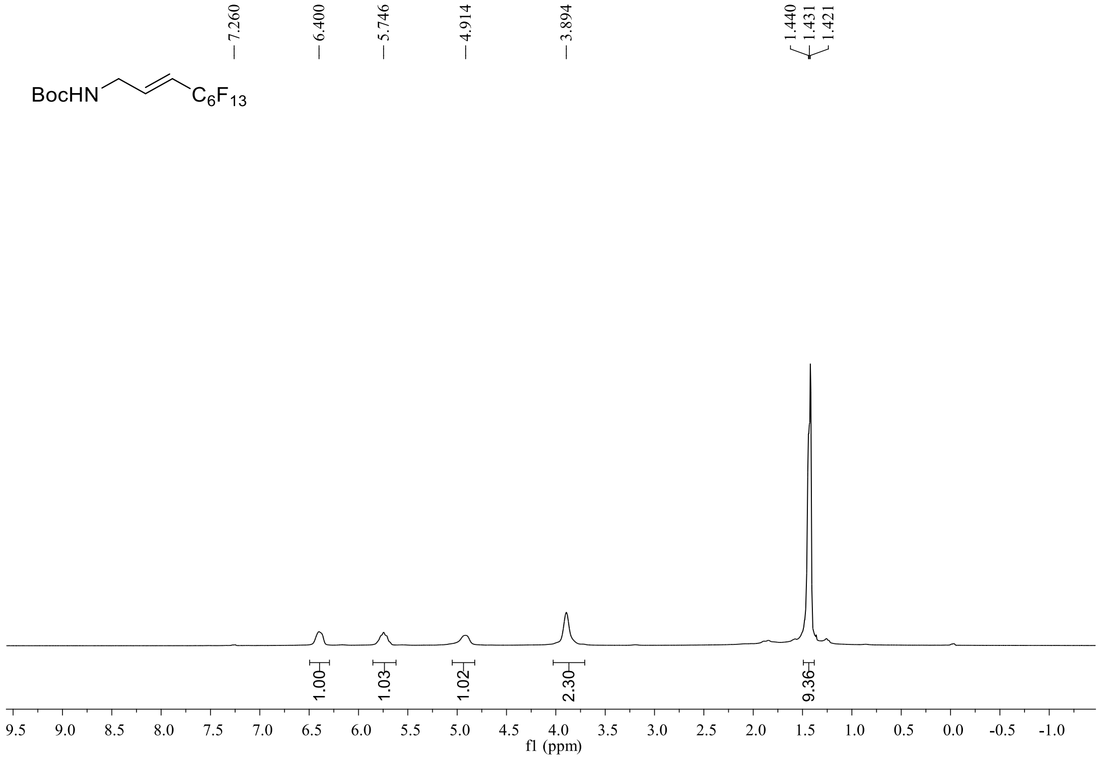

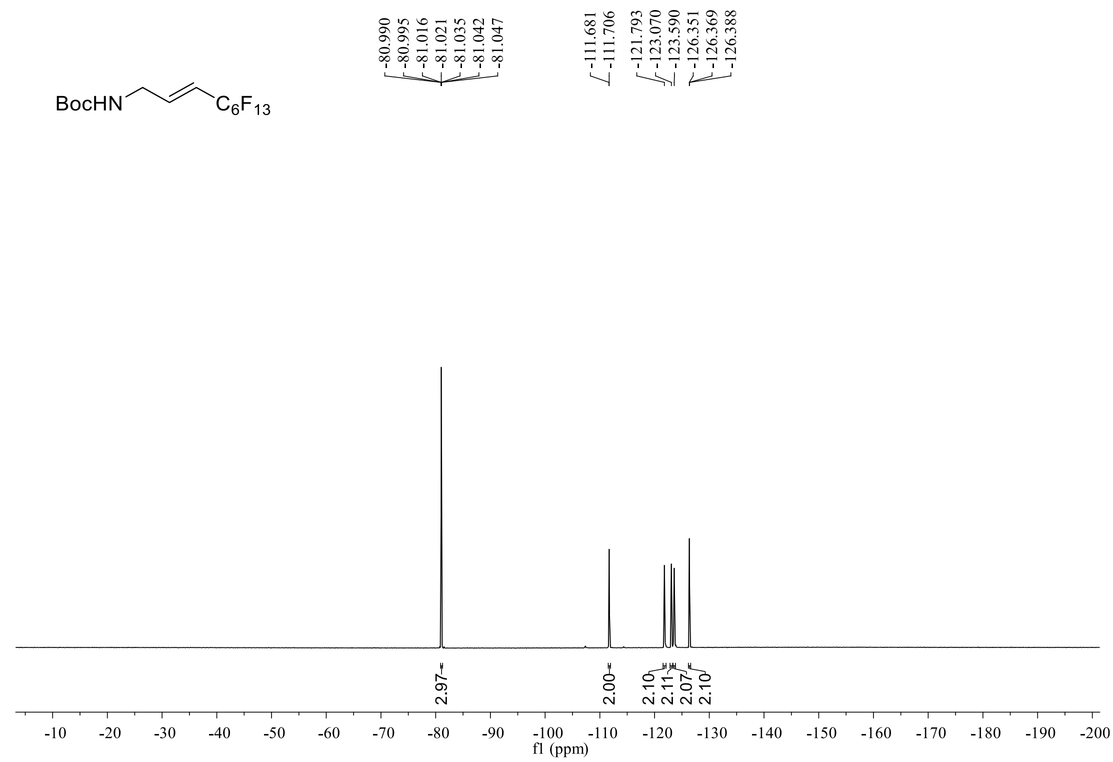
BocHN
C6F13
-80.990
-80.995
-81.016
-81.021
-81.035
-81.042
-81.047
-111.681
-111.706
-121.793
-123.070
-123.590
-126.351
-126.369
-126.388
2.97
2.00
2.10
2.11
2.07
2.10
f1 (ppm)

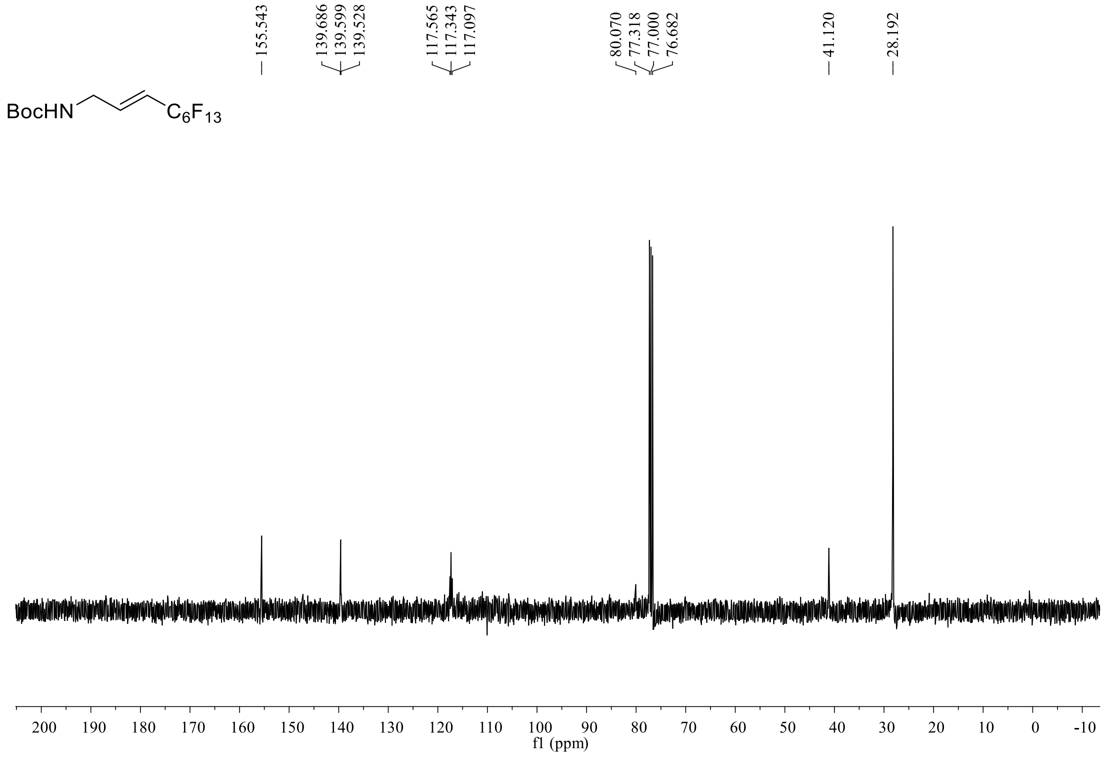
BocHN
C6F13
155.543
139.686
139.599
139.528
117.565
117.343
117.097
80.070
77.318
77.000
76.682
41.120
28.192
f1 (ppm)

**(*E*)-(3,3,4,4,5,5,6,6,7,7,8,8,9,9,10,10,10-heptadecafluorodec-1-en-1-yl)benzene(3s).**

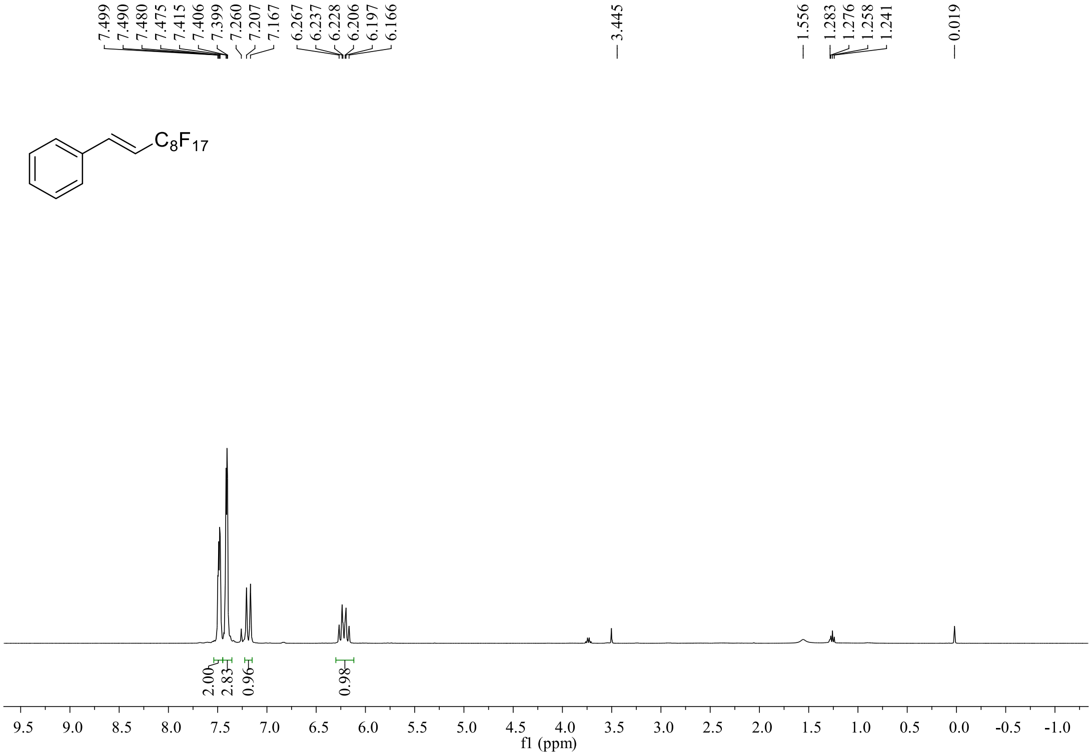


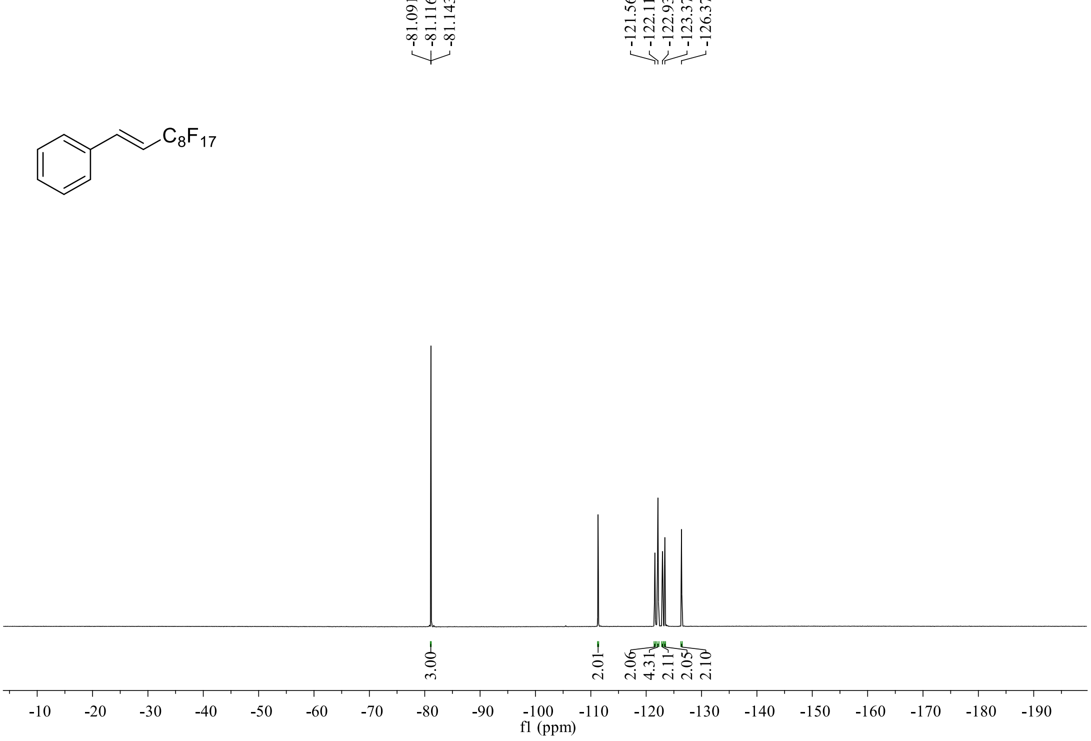

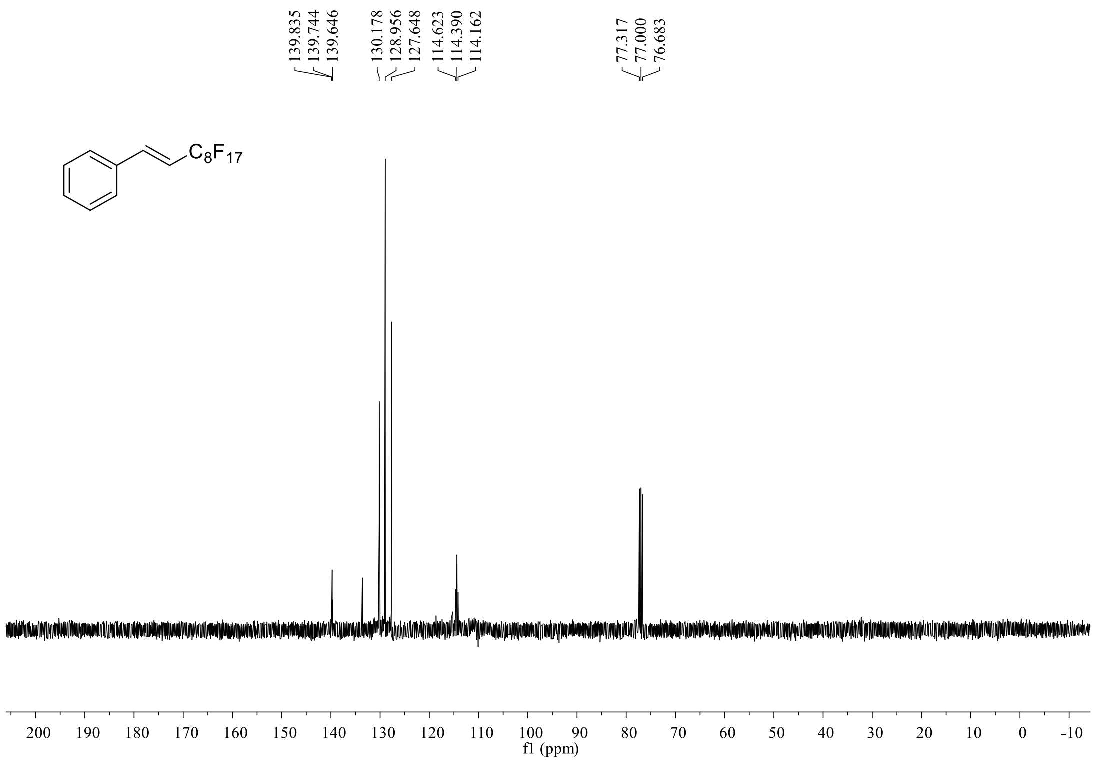
139.835
139.744
139.646
130.178
128.956
127.648
114.623
114.390
114.162
77.317
77.000
76.683
C8F17
200 190 180 170 160 150 140 130 120 110 100 90 80 70 60 50 40 30 20 10 0 -10
f1 (ppm)
